# Intestinal barrier dysfunction assessed by confocal laser endomicroscopy correlates with cirrhosis severity and clinical outcomes^☆^

**DOI:** 10.1016/j.jhepr.2026.102037

**Published:** 2026-09-18

**Authors:** Benedikt Simbrunner, Marcel Sorribas, Jessica Schaerer, Marco Felber, Jordi Codinach, Susana Gomes Rodrigues, Mattias Mandorfer, Annalisa Berzigotti, Michael Trauner, Thomas Reiberger, Johann Hammer, Reiner Wiest

**Affiliations:** 1 Division of Gastroenterology and Hepatology, Department of Medicine III, Medical University of Vienna, Vienna, Austria; 2 Vienna Hepatic Hemodynamic Lab, Division of Gastroenterology and Hepatology, Department of Medicine III, Medical University of Vienna, Vienna, Austria; 3 Clinical Research Group MOTION, Medical University of Vienna, Vienna, Austria; 4 Christian Doppler Laboratory for Portal Hypertension and Liver Fibrosis, Medical University of Vienna, Vienna, Austria; 5 CeMM Research Center for Molecular Medicine of the Austrian Academy of Sciences, Vienna, Austria; 6 Department of Disease Biology, Molecular Cell Biology Group, Almirall, Sant Feliu de Llobregat, Spain; 7 Department of Agri-Food Engineering and Biotechnology, Universitat Politècnica de Catalunya (UPC), Castelldefels, Sant Feliu de Llobregat, Spain; 8 Department of Visceral Surgery and Medicine, Inselspital, Bern University Hospital, University of Bern, Bern, Switzerland; 9 Department for BioMedical Research, Visceral Surgery and Medicine, University of Bern, Bern, Switzerland; 10 Department of Bioengineering, School of Engineering, Universitat Ramon Llull - Institut Químic de Sarrià (URL-IQS), Barcelona, Spain

**Keywords:** Gut barrier, Cirrhosis, Gut–liver axis, Endoscopy, Confocal laser microendoscopy

## Abstract

**Background & Aims:**

Intestinal barrier dysfunction drives complications in advanced chronic liver disease (ACLD). Confocal laser endomicroscopy (CLE) enables direct visualization of transepithelial fluorescein leakage during endoscopy. This study evaluated the link between CLE-assessed intestinal permeability and disease severity in ACLD.

**Methods:**

CLE was performed in two independent cohorts after intravenous fluorescein administration in the small intestine: (1) Bern cohort (n = 4 healthy controls, n = 16 ACLD) and (2) Vienna cohort (n = 48 ACLD). Image analysis included established CLE scores, and novel quantitative assessments measuring fluorescence intensity in three compartments (‘3-compartment-method’; lumen–epithelium–lamina propria) and along the epithelium (‘3-line-method’; base–middle–apex). CLE-derived metrics were correlated with disease stage and biomarkers. In the Vienna cohort, correlation with portal hypertension (HVPG) and liver-related events (decompensation, ACLF, death) was assessed.

**Results:**

Transepithelial fluorescein permeation into the gut lumen was absent in healthy controls but frequent in ACLD, and increased with disease severity (Vienna cohort: compensated *vs.* first decompensation *vs.* further decompensation: 24%, 57%, and 71%, *p* = 0.019). The 3-line and 3-compartment methods robustly distinguished healthy controls from cirrhosis (3-line apex/base ratio: 0.42 ± 0.12 *vs.* ACLD 0.83 ± 0.18, *p* = 0.001), and exhibited lowest interobserver variability. In both cohorts, quantitative permeability measures – especially epithelial 3-line apex/base ratio and 3-line-slopes (all *p* <0.05) – increased across Child-Turcotte-Pugh stages, and correlated with HVPG (apex/base ratio: r = 0.575, *p* <0.001) and liver function parameters (*e.g.* albumin: r = -0.553, *p* <0.001). CLE-based barrier dysfunction was linked to higher liver-related event incidence during follow-up (Cox regression; apex/base ratio: hazard ratio 10.6, 95% CI 1.71–66.0, *p* = 0.011).

**Conclusions:**

CLE identifies high prevalence of intestinal permeability in ACLD, correlating with disease stage and adverse outcomes. The novel 3-line epithelial analysis demonstrated strong associations with portal hypertension and biomarkers reflecting ACLD severity.

**Impact and implications:**

Intestinal barrier dysfunction is a key driver of complications in advanced chronic liver disease. Using CLE during endoscopy, the present study shows that transepithelial fluorescein leakage can be visualized and quantified, and aligns with cirrhosis stage, portal hypertension, and liver-related outcomes. These findings are relevant for hepatologists and translational researchers, as this technique might be used to identify patients with intestinal barrier dysfunction. Because the present analyses are exploratory and derive from two cohorts with limited sample size and requirement for manual image analysis, larger prospective studies and feasibility of (semi-)automated quantification are required before CLE-based permeability assessment can be applied more broadly.

**Clinical Trials Registration:**

The study is registered at ClinicalTrials.gov (NCT03267615).

## Highlights

• CLE-based visualization of gut barrier permeability in patients with cirrhosis performed in two European transplant centers.
• Novel quantitative 3-line/3-compartment CLE analysis methods are reproducible.
• Transepithelial fluorescein leakage is elevated compared with healthy controls and further rises with cirrhosis severity.
• CLE permeability metrics correlate with portal hypertension and liver function.
• Higher CLE-assessed intestinal permeability is linked to liver-related events.

## Graphical abstract

**Figure:**
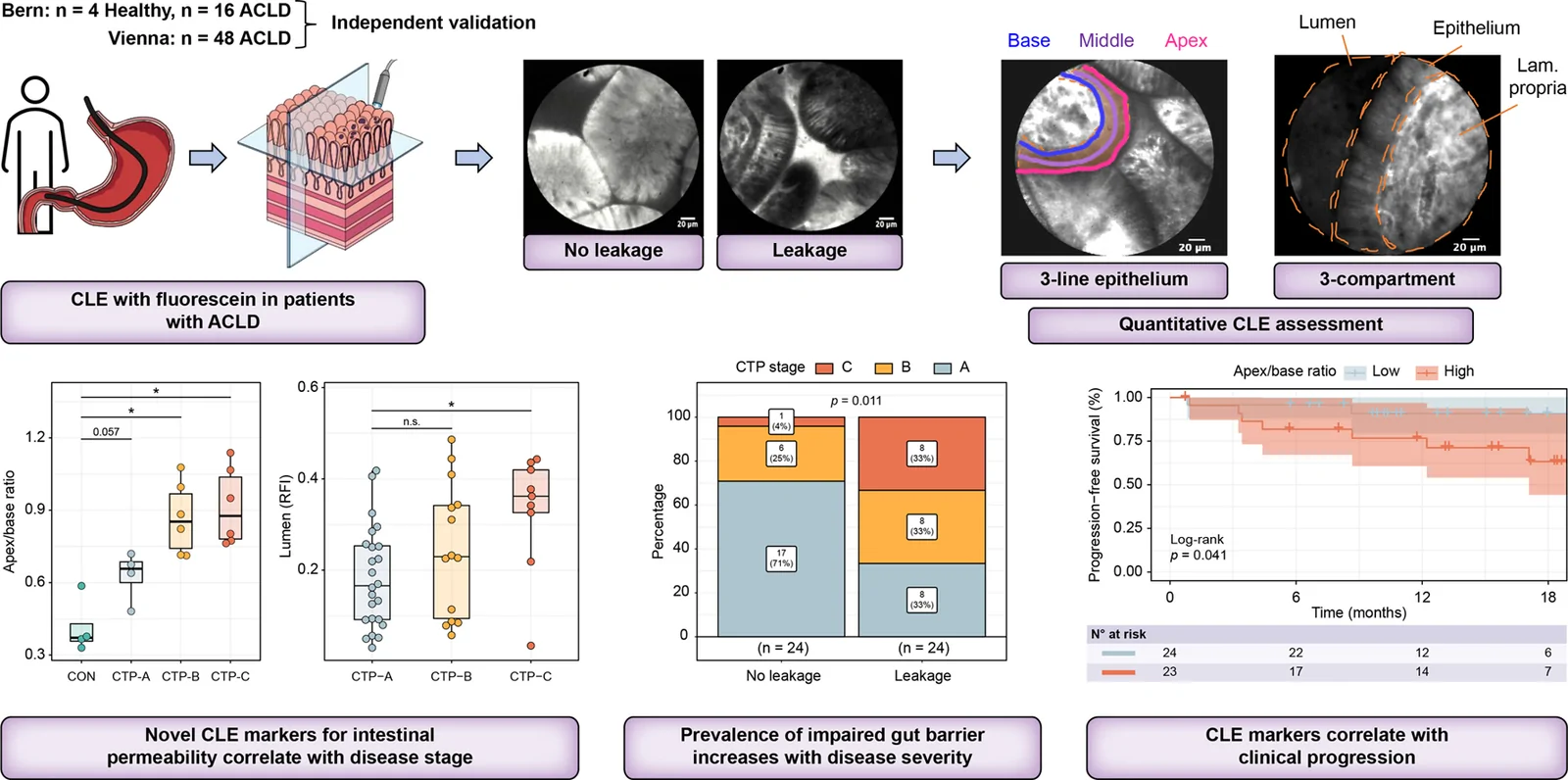

## Introduction

Advanced chronic liver disease (ACLD) is characterized by advanced fibrosis, functional and structural distortion of hepatic tissue, culminating in portal hypertension and liver dysfunction. Beyond intrinsic hepatic damage, the gut–liver axis plays a central role in the pathogenesis and progression of cirrhosis.^1^^,^^2^ Intestinal barrier dysfunction in patients with ACLD results in increased intestinal permeability, facilitates bacterial translocation and the systemic dissemination of pathogen-associated molecular patterns (PAMPs). This perpetuates inflammation and immune activation, thus exacerbating liver injury and contributing to progression of portal hypertension and fibrosis.[3], [4], [5]

These processes establish a vicious cycle of gut and liver dysfunction; wherein gut barrier failure amplifies inflammation and hepatic decompensation. Despite its critical role in disease progression, the clinical assessment of intestinal permeability remains challenging.[6], [7], [8] Conventional methods, including serum biomarkers and indirect assays, often lack specificity and spatial resolution. Furthermore, mucosa biopsies, although informative, are impractical for routine clinical assessment of barrier function because of delayed results.^9^ These limitations underscore the need for direct, *in vivo* visualization of intestinal barrier integrity.

Confocal laser endomicroscopy (CLE) is an advanced imaging technique that enables *in vivo*, high-resolution assessment of the intestinal mucosa at the cellular and subcellular levels during endoscopy.[10], [11], [12], [13] By integrating a confocal microscope into an endoscope and using fluorescein contrast, CLE provides insight into mucosal integrity by assessing fluorescein leakage, a marker of barrier dysfunction.

The primary aim of this study was to evaluate whether CLE-based assessment of intestinal barrier function detects increased transepithelial permeability that relates to disease severity in patients with ACLD. To this end, we developed and evaluated two novel quantitative CLE analysis techniques (3-line and 3-compartment methods) alongside established qualitative scores, and used a small group of healthy controls to test whether these readouts already deviate in early (compensated) ACLD. As exploratory objectives, we examined the relationship between CLE-derived permeability metrics and established markers of disease severity and liver-related events to explore their potential clinical relevance.

## Patients and methods

### Study design and clinical characterization

Patients with ACLD undergoing routine gastrointestinal endoscopy with CLE were prospectively recruited at two European tertiary centers: University Hospital Bern (Bern, Switzerland) and the Medical University of Vienna (Vienna, Austria).

In the Bern cohort, patients with ACLD undergoing screening or surveillance for esophageal varices were eligible. Exclusion criteria were recent gastrointestinal bleeding (<3 months) and active malignancy. Furthermore, healthy controls were recruited among individuals undergoing endoscopy for functional gastrointestinal complaints, provided that they had no relevant comorbidities, were not receiving ongoing medication, and showed no gastrointestinal pathology during endoscopy. General exclusion criteria were a known allergy to fluorescein, pregnancy or breast feeding, or impaired renal function (estimated glomerular filtration rate <45 ml/min).

In the Vienna cohort, patients with ACLD were included when at least one of the following criteria were present: HVPG ≥6 mmHg, liver stiffness measurement ≥10 kPa, histological fibrosis stage F3/F4, or radiological evidence of cirrhosis. Patients with posthepatic or presinusoidal portal hypertension or previous liver transplantation were not considered for the study. Hepatic decompensation events previous to/at the timepoint of endoscopy were determined according to Austrian^14^ and European guidelines^15^ and Baveno VII consensus criteria.^16^ Laboratory parameters were obtained from electronic hospital records and were analyzed by the Department of Laboratory Medicine at the Medical University of Vienna, following International Organization for Standardization (ISO)-certified measurement protocols.

### Endoscopic procedures and confocal laser endomicroscopy

Informed consent was obtained before endoscopy, which was performed under conscious sedation utilizing standard endoscopes (GIF-HQ190 endoscope and CV-190 imaging system, Olympus Corporation, Tokyo, Japan). CLE was performed during the endoscopy procedure using a Cellvizio probe (Mauna Kea Technologies, Paris, France) to obtain high-resolution, *in vivo* imaging of the second part of the duodenum. A single intravenous bolus of 2.5-5 ml fluorescein (SERB Pharmaceuticals, Paris, France) was used as a systemic contrast agent to enhance visualization and assess intestinal permeability. The small intestinal mucosa was inspected for up to 8 min post-fluorescein injection, focusing on identifying luminal transposition of fluorescein. Analysis was performed on selected images acquired within 2 min after first appearance of fluorescein in the microcirculation seen on CLE after injection, limiting the additional procedural time required for CLE. The presence of fluorescein leakage into the gut lumen was assessed, in line with previously published Watson scoring,^13^ which distinguishes between absence of leakage into the gut lumen (referred to as ‘No leakage’) and the presence of leakage with or without cell shedding (‘Leakage’).

### CLE image analysis

For the assessment of CLE-based qualitative and quantitative readouts of transepithelial fluorescein permeation, 10 representative images or more were selected and analyzed using Fiji (ImageJ).

#### Image analysis

Cellvizio Viewer software (Cellvizio Viewer Version 1.4.2; Mauna Kea Technologies, Paris, France) was used to extract the photograms from the CLE video. An adequate image was defined as one that fulfilled each of the following criteria: presence of vasculature/vessel, epithelium and lumen, and high contrast/delineation of morphology (Fig. 1). The analysis of the confocal images was conducted in a blinded manner by two independent observers at each center. Videos were reviewed and images of sufficient quality taken after first appearance of fluorescence were analyzed. Observers at each center analyzed the same pre-selected images, blinded to the measurements of the other observer.

**Fig. 1:**
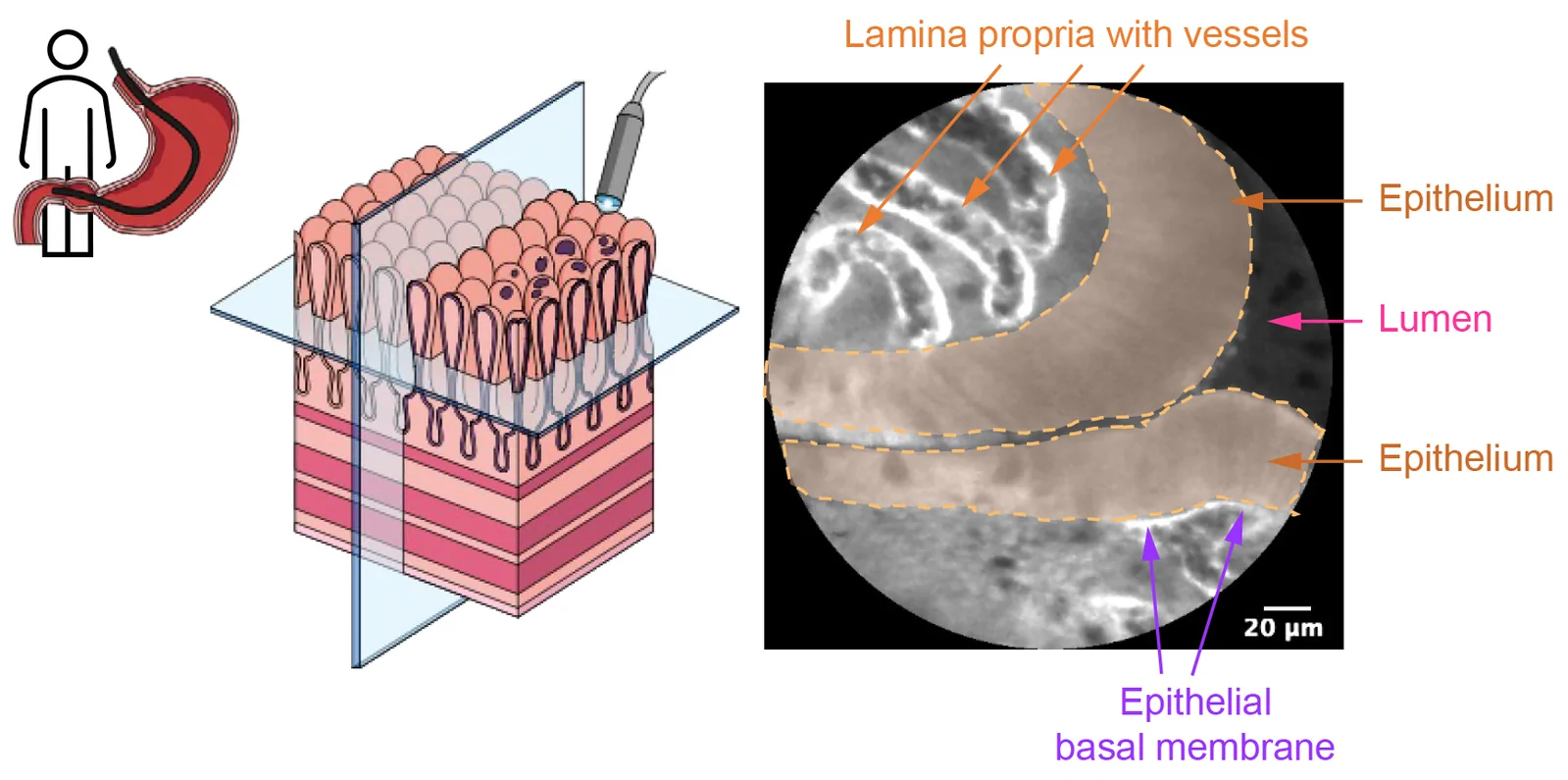
Confocal laser endomicroscopy (CLE) of the small intestinal mucosa in patients with advanced chronic liver disease. Representative confocal laser endomicroscopy images of the small intestinal mucosa after intravenous fluorescein administration, demonstrating epithelial architecture, lamina propria with vessels, and luminal compartment.

#### Three-compartment-analysis

The lamina propria, epithelium, and lumen compartments of a villus were manually outlined using the polygon tool in ImageJ. For each compartment the area and the mean fluorescence intensity (MFI) were computed (Fig. 2A). The sum of MFIs from all three compartments was defined as 100%, and the proportion of fluorescence intensity within each compartment was expressed as a percentage of this total, thereby defining the relative fluorescence intensity (RFI).

**Fig. 2:**
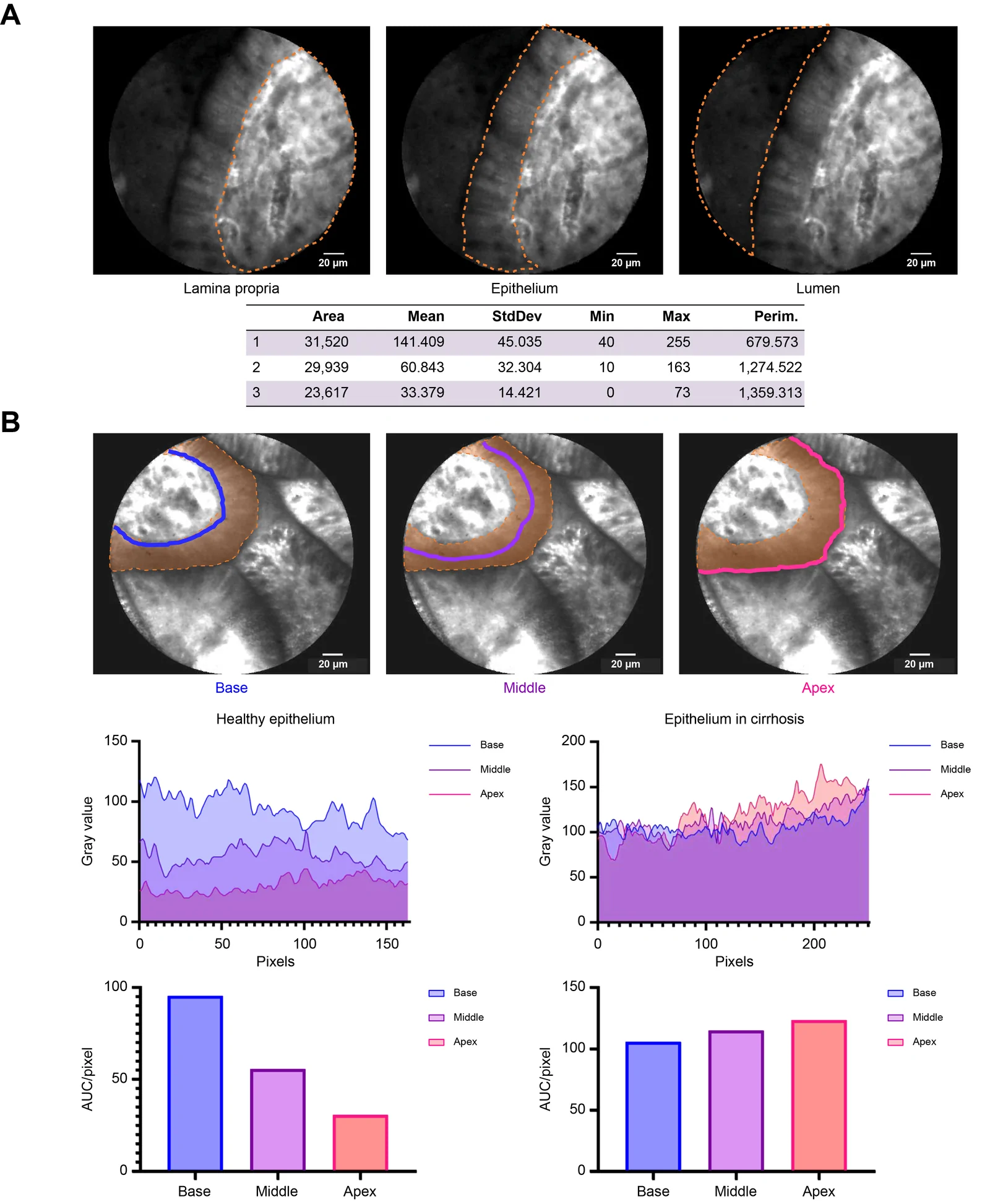
Novel quantitative CLE image analysis: 3-compartment and 3-line methods. Schematic overview of quantitative CLE image analysis. (A) Three-compartment analysis of epithelium, lamina propria, and lumen. (B) Three-line analysis assessing fluorescence intensity along the base–middle–apex axis of the intestinal epithelium.

#### Three-line method assessing gut epithelial barrier

Fluorescence intensity (FI, ‘gray value’) within the epithelial layer was measured at three predefined levels along the epithelial lining using Image J (Fig. 2B): base, middle, and apex. For each line, pixel-wise gray values were averaged to obtain the MFI. Ratios between the three epithelial levels and slopes were calculated based on the assumption that the amount of fluorescein determines fluorescence intensity as it permeates from the basal to the apical epithelial layers. Further details are delineated in the Supplementary material.

#### Confocal leak score

The Confocal Leak score (CLS) was assessed as previously described by Chang and colleagues^17^ in the context of inflammatory bowel disease (IBD). A detailed method can be found in the Supplementary material.

#### Gap score

Epithelial gap density was determined as previously described in the context of irritable bowel syndrome.^18^ A detailed methodological description is delineated in the Supplementary material.

### Liver-related events during the follow-up period

Liver-related events denoting disease progression were recorded during follow-up in the Vienna cohort, that is, the incidence of first decompensation, further decompensation, acute-on-chronic liver failure, and liver-related death (LRD). Patients were followed at routine clinical visits (every 6 months in compensated ACLD [cACLD]; every 3 months in decompensated ACLD, or shorter as clinically indicated), with decompensation events systematically recorded at each visit, as previously described.^19^ Patients were censored at the date of last clinical contact or liver transplantation in case no event occurred. In patients with cACLD, first decompensation was defined as the incidence of clinically evident ascites, variceal bleeding, or overt hepatic encephalopathy (HE). In patients with decompensated ACLD (dACLD), further decompensation was defined as the incidence of a second decompensating event or recurrent/refractory ascites, overt HE, variceal bleeding, and/or incidence of spontaneous bacterial peritonitis (SBP), hepatorenal syndrome–acute kidney injury (HRS-AKI), in line with Baveno VII consensus criteria.^16^ Furthermore, the incidence of acute-on-chronic liver failure according to guidelines by the EASL was recorded.^20^

### Statistical analysis

Statistical analyses were performed using R 4.3.2 (R Foundation for Statistical Computing, Vienna, Austria). Continuous variables were compared using Student’s *t* test or Mann-Whitney *U* test for comparisons of two groups, and one-way ANOVA or Kruskal-Wallis tests with Benjamini-Hochberg-adjusted pairwise Wilcoxon rank-sum tests, as appropriate, for comparisons of three groups. Kaplan-Meier curves and log-rank tests were used to visualize and compare the incidence of liver-related events between groups (‘survival’ package). Univariable Cox regression analyses were performed to evaluate whether individual parameters were associated with liver-related events (hazard ratios with 95% CIs). Interobserver agreement was assessed using the intraclass correlation coefficient (ICC) based on a two-way random-effects model with absolute agreement. Ninety-five percent confidence intervals were calculated for ICC estimates. Principal component analysis (PCA) was performed on centered and scaled variables using the ‘FactoMineR’ package, with visualization of biplots, scree plots, variable contributions, and stage-stratified individual factor maps generated using ‘factoextra’. Additional information on statistical analyses is delineated in the Supplementary material.

### Ethical considerations

The study was conducted in accordance with the principles of the Declaration of Helsinki and its amendments and approved by the local ethics committees (Vienna: EK 1262/2017; 1239/2023; Bern: KEK-2020-00873). Patients and healthy individuals provided written informed consent for endoscopic procedures.

## Results

### Patient characteristics

The Bern cohort comprised four healthy individuals and 16 patients with ACLD. Clinical and laboratory characteristics are summarized in Table S1. Briefly, patients had a median age of 53 (IQR: 50–63) years, were predominantly male (n = 13/16, 81%), and alcohol-related liver disease (ALD; n = 7, 44%) was the most prevalent etiology of ACLD. The distribution of patients across Child-Turcotte-Pugh (CTP) stages comprised n = 4 (25%) CTP-A, n = 6 (37.5%) CTP-B, and n = 6 (37.5%) CTP-C.

The Vienna cohort comprised 48 patients with ACLD with a median age of 55 years (IQR: 43–69) and 69% were male. Patient characteristics are summarized in Table 1. Median hepatic venous pressure gradient (HVPG) was 17 mmHg (IQR: 14–21; available in n = 43; median time interval to endoscopy: 2.20 [0.00–18.0] months), median model for end-stage liver disease (MELD) score was 12 (IQR: 9–15). ALD was the most common etiology of liver disease, accounting for 54% of the cohort. According to the CTP classification, 52% of patients were CTP-A (n = 25), 29% CTP-B (n = 14), and 19% CTP-C (n = 9). MELD and HVPG gradually increased across clinical stages (Table 1).

**Table 1:** Characteristics of the Vienna cohort stratified by clinical disease stages: cACLD, first decompensation, and further decompensation.

	Overall cohort	cACLD	FirstDec	FurtherDec	*p* value
	N = 48	n = 17	n = 14	n = 17	
CTP score	6.00 [5.00; 8.00]	5.00 [5.00; 5.00]	6.50 [5.25; 7.00]	8.00 [7.00; 12.0]	<0.001
CTP stage, n (%):					<0.001
A	25 (52.1)	16 (94.1)	7 (50.0)	2 (11.8)	
B	14 (29.2)	1 (5.88)	6 (42.9)	7 (41.2)	
C	9 (18.8)	0 (0.00)	1 (7.14)	8 (47.1)	
MELD score	12.0 [9.00; 15.2]	10.0 [8.00; 12.0]	11.0 [10.0; 13.5]	20.0 [12.0; 21.0]	0.001
HVPG (mmHg)	17.4 (4.94)	14.6 (4.57)	18.6 (5.80)	19.0 (3.26)	0.030
Etiology, n (%):					0.471
ALD	26 (54.2)	7 (41.2)	7 (50.0)	12 (70.6)	
Cholestatic	6 (12.5)	3 (17.6)	3 (21.4)	0 (0.00)	
MASLD	3 (6.25)	1 (5.88)	1 (7.14)	1 (5.88)	
ALD and viral	5 (10.4)	2 (11.8)	2 (14.3)	1 (5.88)	
Other	6 (12.5)	2 (11.8)	1 (7.14)	3 (17.6)	
Viral	2 (4.17)	2 (11.8)	0 (0.00)	0 (0.00)	
Sex (n, %):					0.075
F	15 (31.2)	2 (11.8)	7 (50.0)	6 (35.3)	
M	33 (68.8)	15 (88.2)	7 (50.0)	11 (64.7)	
Age (years)	55.4 (13.7)	59.7 (11.7)	52.3 (17.0)	53.5 (12.3)	0.263
Varices (n, %):					0.054
No	11 (22.9)	6 (35.3)	3 (21.4)	2 (11.8)	
Small	16 (33.3)	8 (47.1)	2 (14.3)	6 (35.3)	
Large	21 (43.8)	3 (17.6)	9 (64.3)	9 (52.9)	
Ascites (n, %):					<0.001
No	23 (47.9)	17 (100)	5 (35.7)	1 (5.88)	
Mild	20 (41.7)	0 (0.00)	8 (57.1)	12 (70.6)	
Severe/recurrent	5 (10.4)	0 (0.00)	1 (7.14)	4 (23.5)	
HE (n, %):					<0.001
No	40 (83.3)	17 (100)	14 (100)	9 (52.9)	
Mild	7 (14.6)	0 (0.00)	0 (0.00)	7 (41.2)	
Severe/recurrent	1 (2.08)	0 (0.00)	0 (0.00)	1 (5.88)	
Fluorescein leakage (n, %)					0.019
Absent	24 (50.0)	13 (76.5)	6 (42.9)	5 (29.4)	
Present	24 (50.0)	4 (23.5)	8 (57.1)	12 (70.6)	

Statistical Analysis: Kruskal-Wallis test or ANOVA were used to compare continuous variables. Group comparisons of categorical variables were performed using Χ^2^ or Fisher’s Exact test. ALD, alcohol-related liver disease; cACLD, compensated advanced chronic liver disease; CTP, Child-Turcotte-Pugh; HVPG, hepatic venous pressure gradient; MASLD, metabolic dysfunction-associated liver disease; MELD, model for end-stage liver disease.

### Exploration of novel CLE-based intestinal barrier analysis

To assess transepithelial fluorescein permeation of the intestinal mucosa, the 3-line method was applied in healthy controls and patients with cirrhosis from the Bern cohort (Fig. 3A and Fig. S1). In healthy controls, mean RFI was highest at the basal epithelial layer and progressively decreased towards the middle and apical layers. This gradient was also present in ACLD (Fig. S1A), but RFI values differed from controls: patients with ACLD exhibited significantly higher apical RFI compared with healthy controls (0.29 [0.28–0.30] *vs.* 0.18 [0.16–0.20], *p* = 0.003), whereas basal RFI was lower (0.39 [0.37–0.40] *vs.* 0.50 [0.48–0.51], *p* = 0.003). No significant group differences were observed at the middle epithelial layer. Consequently, the apex/base ratio significantly increased in patients with ACLD compared with healthy controls (Healthy: 0.42 ± 0.12, ACLD: 0.83 ± 0.18, *p* = 0.001), indicating enhanced transepithelial fluorescein passage towards the luminal surface in cirrhosis. Stratification by CTP stages revealed a stepwise increase of apex/base ratio with advancing disease severity: 0.42 ± 0.12 in healthy controls, 0.63 ± 0.10 in CTP-A, 0.87 ± 0.15 in CTP-B, and 0.92 ± 0.16 in CTP-C patients (overall *p* <0.001; Fig. 3A). Similar dynamics were present when stratifying patients by the presence of ascites (Fig. S1).

**Fig. 3:**
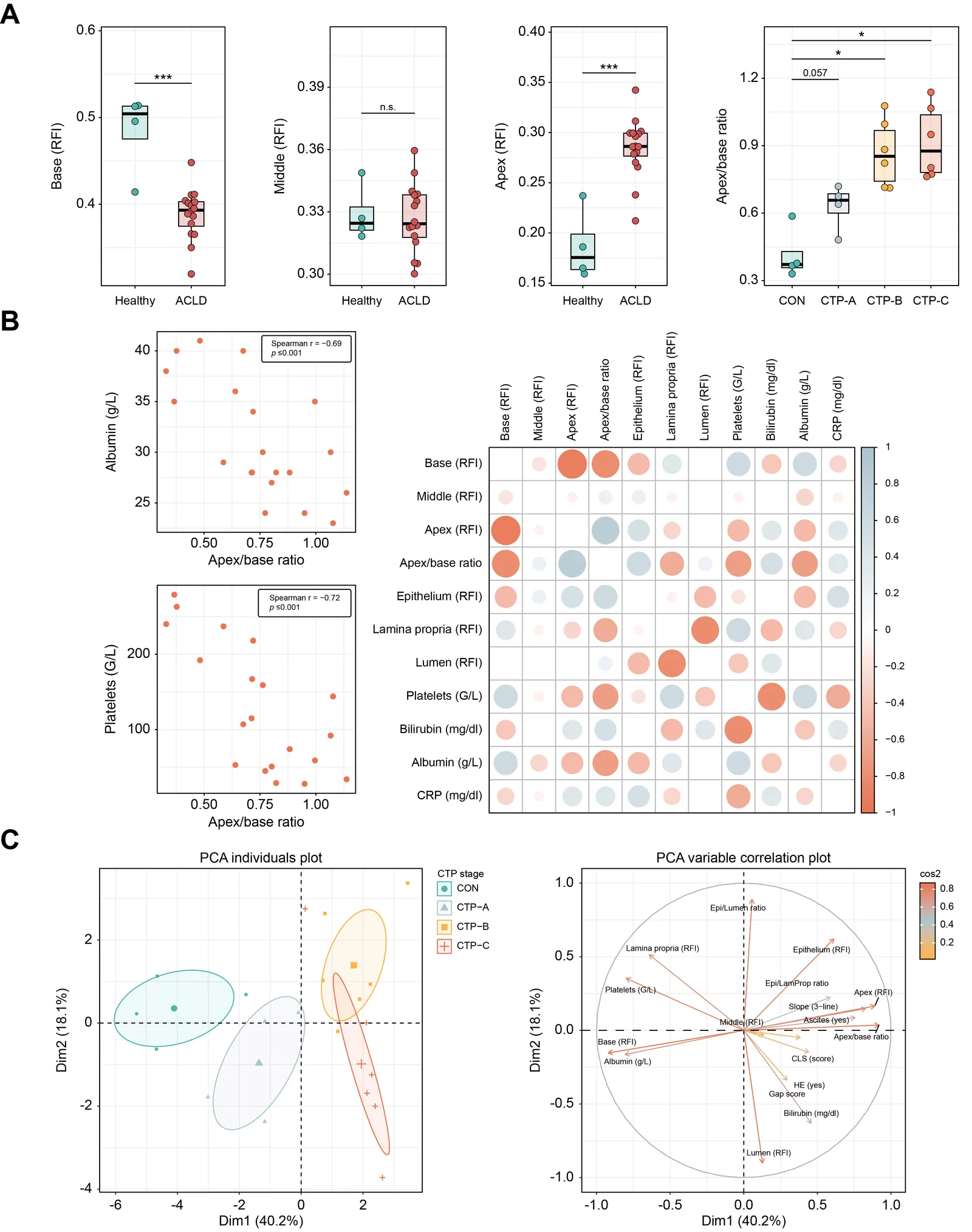
Application of quantitative CLE analysis in the exploration cohort (Bern). (A) Three-line analysis of epithelial fluorescence intensity in the exploration cohort, including healthy controls. Group comparisons were performed using Wilcoxon rank-sum tests with Benjamini-Hochberg correction for multiple testing, where applicable. (B) Correlation matrix of CLE-derived and laboratory parameters and correlation plots of apex/base ratio with serum albumin and platelet levels. Correlations were evaluated using Spearman rank correlation. (C) Principal component analysis (PCA) integrating CLE-derived metrics with clinical and laboratory variables. PCA was performed on scaled variables (unit variance) using complete-case observations. Statistics legend: (ns) not significant; (∗) *p* <0.05; (∗∗) *p* <0.01; (∗∗∗) *p* <0.001. CRP, C-reactive protein; CON, healthy controls; RFI, relative fluorescence intensity.

In the 3-compartment analysis (lamina propria, epithelium, and lumen), a physiological fluorescence gradient was observed across both healthy controls and patients with ACLD, characterized by decreasing RFI from the lamina propria to the epithelium and further into the intestinal lumen (Fig. S2). This pattern suggests preserved compartmental organization of fluorescein distribution. Lamina propria RFI was significantly reduced in patients with advanced cirrhosis compared with healthy controls (0.40 [0.39–0.45] *vs.* 0.57 [0.53–0.59], *p* = 0.018), most prominently in patients who had CTP-C (Fig. S2). In contrast, epithelial (0.34 ± 0.07 *vs.* 0.28 ± 0.04, *p* = 0.032) and luminal RFI (0.23 ± 0.11 *vs.* 0.17 ± 0.04; *p* = 0.096) were higher in patients with ACLD compared with healthy controls, consistent with enhanced transepithelial fluorescein leakage and redistribution towards the luminal compartment in advanced disease.

Together, these findings support progressive leakage of fluorescein across the epithelium into the lumen indicating an impairment of the intestinal barrier with increasing cirrhosis severity.

To compare the new quantitative CLE readouts against previously established scores, CLS and Gap score (Fig. S3) were assessed. In summary, both CLS and Gap score exhibited numerical trends toward increased mucosal barrier disruption, however, neither method was able to robustly discriminate between cirrhosis stages (see Supplementary material).

Furthermore, correlation analyses were performed to explore interactions between the novel CLE parameters and routine blood analyses used in the context of liver disease (Fig. 3B). Interestingly, apex/base ratio showed a strong negative correlation with serum albumin (r = -0.694, *p* <0.001) and platelet levels (r = -0.716, *p* <0.001), and a significant positive correlation with bilirubin (r = 0.478, *p* <0.001) denoting surrogates of liver function and portal hypertension, respectively.

PCA integrating clinical, laboratory, and CLE-derived parameters demonstrated separation of healthy controls from patients with ACLD, as well as stage-dependent clustering across CTP stages. Notably, variables derived from the quantitative 3-line/3-compartment CLE approaches were among the strongest contributors to the principal components differentiating patients (Fig. 3C and Fig. S4; Supplementary Results).

### Validation of CLE-based intestinal permeability analysis in independent cohort

In the Vienna cohort, transepithelial fluorescein permeation into the gut lumen (qualitative assessment, following Watson grading system^13^) showed a stepwise increase across disease stages, being significantly more prevalent in decompensated cirrhosis: 24% of patients with compensated ACLD, 57% with first decompensation, and 71% with further decompensation (*p* = 0.019; Fig. S5). As expected, stratification by CTP stages yielded a similar observation: 32% in CTP-A, 57% in CTP-B, and 89% in CTP-C (*p* = 0.011; Fig. 4A). As demonstrated in Table 1 and Table S2, established clinical and laboratory parameters reflecting disease severity such as MELD and HVPG increased across mentioned disease stages, emphasizing the representativeness of cohort characteristics.

**Fig. 4:**
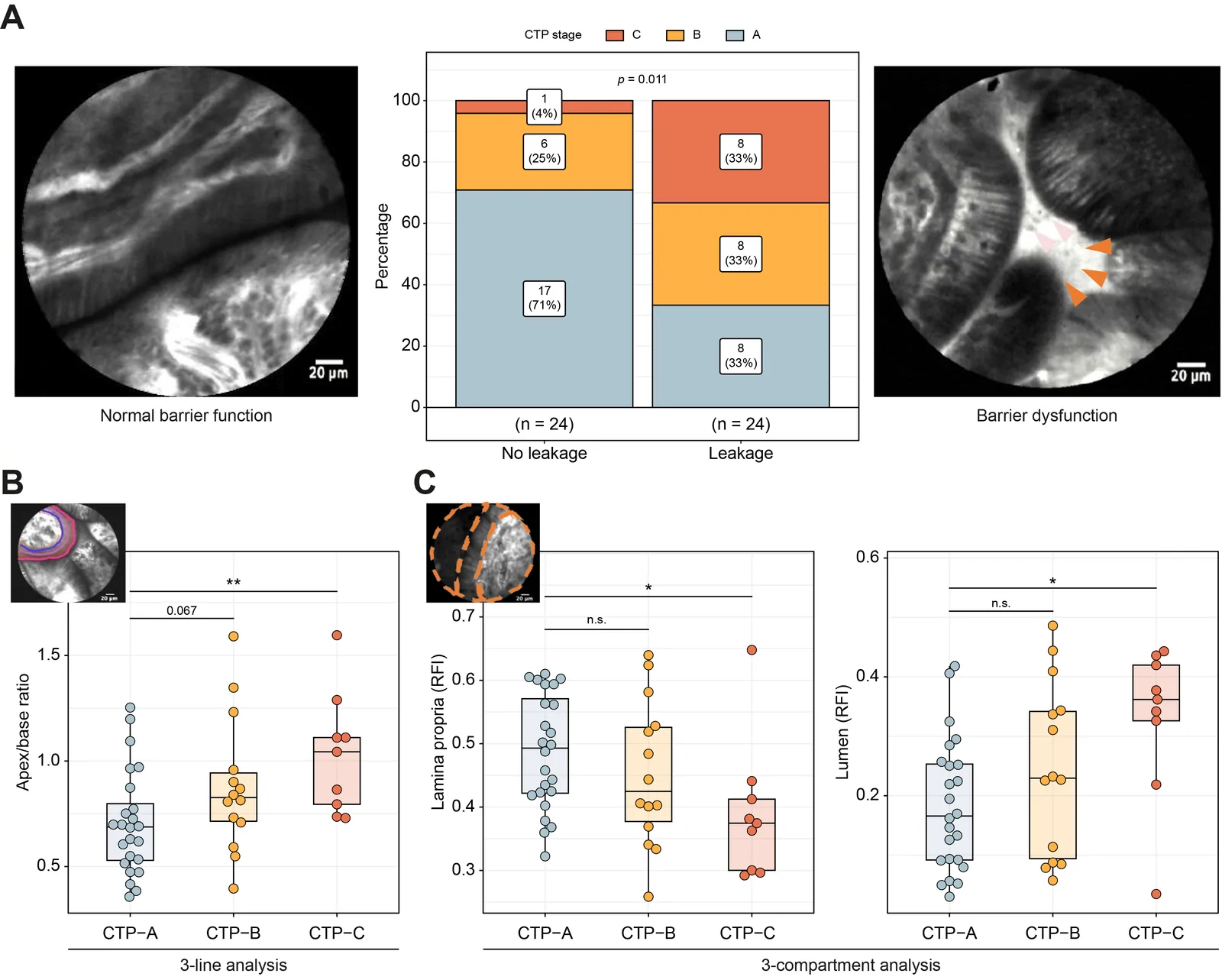
Application of CLE in the validation cohort (Vienna). (A) Representative CLE images illustrating absent and present intraluminal fluorescein leakage. Leakage prevalence was compared across CTP stages using the Χ^2^ test. (B) Three-line analysis of epithelial fluorescence intensity stratified by CTP stage. (C) Three-compartment analysis of fluorescence distribution across epithelial, lamina propria, and luminal compartments by CTP stage. Group comparisons were performed using Wilcoxon rank-sum tests with Benjamini–Hochberg correction for multiple testing. Statistics legend: (ns) not significant; (∗) *p* <0.05; (∗∗) *p* <0.01; (∗∗∗) *p* <0.001. CTP, Child-Turcotte-Pugh, RFI, relative fluorescence intensity.

Application of the 3-line and 3-compartment analysis technique in the Vienna ACLD cohort confirmed external reproducibility of the Bern cohort results regarding its relationship with disease stages. Of note, one patient was excluded for 3-line/3-compartment analyses because of technical issues with white balance of images.

Apex/base ratio, apex/middle ratio, and 3-line slopes significantly increased across CTP stages (Fig. 4B and Fig. S6). These observations were paralleled by increasing lumen and decreasing lamina propria RFI (Fig. 4C), representing quantitative readouts of transepithelial fluorescein leakage and intestinal permeability.

Concordant trends for 3-line readouts were observed when stratifying patients into cACLD, first decompensation, and further decompensation; while comparison of 3-compartment readouts did not attain statistical significance (Fig. S6). Finally, 3-line/3-compartment readouts were also aligned with the presence *vs.* absence of ascites, representing a further aspect of independent validation across cohorts (Fig. S7). Results on Gap score and CLS showed similar trends in the validation cohort as observed in the exploration cohort (Fig. S8).

### Correlation between CLE-based barrier dysfunction and relevant biomarkers in cirrhosis

Quantitative results from the 3-line and 3-compartment analysis were correlated with indicators of liver function and disease severity in the Vienna cohort. Most relevant findings are visualized by volcano plots (Fig. 5A and Fig. S9).

**Fig. 5:**
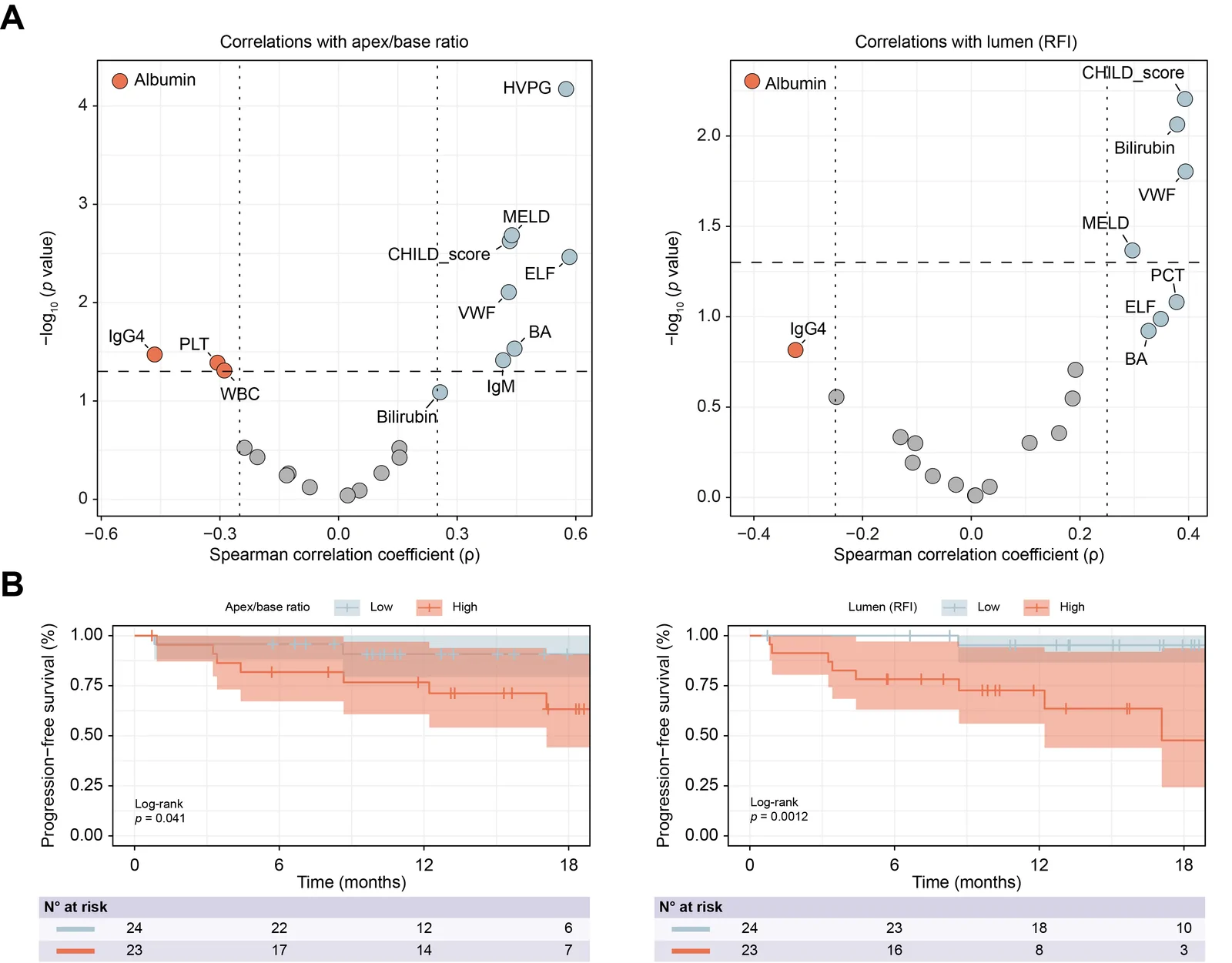
Associations between CLE-derived parameters and clinical features, and their prognostic value for liver-related events. (A) Correlation analysis of CLE-derived parameters, (B) Incidence of liver-related events during the follow-up period after stratification by CLE parameters. (A) Volcano plots illustrating Spearman rank correlations between the CLE readouts and clinical as well as laboratory parameters. The x-axis shows the Spearman correlation coefficient (ρ) and the y-axis shows -log_10_(*p* value); dashed and dotted lines indicate nominal significance and effect size thresholds, respectively. (B) Kaplan–Meier curves indicating disease progression-free follow-up (composite endpoint: first/further decompensation, ACLF, and liver-related death) were compared by log-rank test. Groups are stratified by median CLE parameters. ACLF, acute-on-chronic liver failure; BA, serum bile acids; CHILD, Child-Pugh score, CLE, confocal laser endomicroscopy; ELF®, Enhanced Liver Fibrosis score, HVPG, hepatic venous pressure gradient, Ig, immunoglobulin, MELD, model for end-stage liver disease; PCT, procalcitonin; RFI, relative fluorescence intensity; VWF, von Willebrand factor; WBC, white blood cells.

Apex/base ratio (r = 0.575, *p* <0.001) showed the strongest positive correlation to HVPG, and the strongest negative link to serum albumin (r = -0.553, *p* <0.001); while also correlating with MELD (r = 0.438, *p* <0.001), CTP score (r = 0.433, *p* <0.001), and platelet (PLT) count (r = -0.306, *p* <0.05). Similar correlation patterns and coefficients were observed for apex-middle ratio and the 3-layer slopes.

Furthermore, the link between CLE parameters and advanced serum biomarkers available in a patient subgroup were explored (available values indicated in Table S3). Importantly, 3-line parameters showed strong to moderate correlation with the Enhanced Liver Fibrosis (ELF®) score (apex/base: r = 0.584, apex/middle: r = 0.603, slope: r = 0.564), Von Willebrand Factor antigen (VWF) (apex/base: r = 0.431, apex/middle: r = 0.379, slope: r = 0.422), total serum bile acids (BA) (apex/base: r = 0.445, apex/middle: r = 0.533, slope: r = 0.430), and total IgM concentrations (apex/base: r = 0.416, apex/middle: r = 0.478, slope: r = 0.400; all *p* <0.05).

PCA demonstrated separation of patients according to Baveno clinical stages, with CLE-derived parameters among the strongest contributors to the principal components differentiating patients. Notably, variables derived from the novel quantitative CLE approaches were prominently represented among the top contributing features (Fig. S10; Supplementary Results).

### CLE barrier function and prognosis

During a median transplant-free follow-up of 13.2 months (IQR 9.85–18.3 months) in the Vienna cohort, 10 patients (21%) experienced an adverse liver-related event (LRE; composite endpoint): further decompensation in seven patients and ACLF in three patients. Among patients experiencing LRE, three patients experienced SBP, and three patients progressed to liver-related death. Two patients were censored at liver transplantation. Exploratory analyses on the relationship between CLE and clinical outcome were performed.

Importantly, CLE-assessed barrier dysfunction as reflected by presence of luminal fluorescein leakage (*vs.* absence of leakage^13^) was associated with a higher incidence of adverse LRE (log-rank *p* = 0.003; 25% *vs.* 5% at 12 months; 43% *vs.* 5% at 18 months; Fig. S11).

Consequently, quantitative 3-line and 3-layer analyses also showed prognostic value for LRE. First, stratification by median quantitative readouts discriminated between patients with high *vs*. low risk for disease progression (Fig. 5B and Fig. S11). Second, Cox proportional hazard models (Table 2) showed that MFI in the lumen was linked to a higher LRE incidence (hazard ratio [HR] 1.02, 1.01–1.03, *p* = 0.002), while higher MFI in the lamina propria was linked to a lower risk (HR 0.95, 0.91-0.99, *p* = 0.017), denoting opposing trends consistent with transepithelial fluorescein shift into the intestinal lumen. For the 3-line method, apex/base ratio (HR 10.6, 1.71-66.0, *p* = 0.011), apex/middle ratio (HR 19.1, 2.10-173, *p* = 0.009), and 3-line slopes (HR 1.05, 1.01-1.10, *p* = 0.013) correlated with LRE incidence during follow-up.

**Table 2:** Cox regression models for the prediction of adverse liver-related events.

Parameter	HR	95% CI	*p* value
Age (per year)	0.99	0.95–1.04	0.879
HVPG (per mmHg)	1.13	0.98–1.30	0.081
MELD (per point)	1.26	1.12–1.41	**<0.001**
Albumin (per g/L)	0.87	0.78–0.96	**0.006**
Luminal leakage (y)	11.9	1.50–95.0	**0.019**
Epithelium (MFI)	1.02	0.99–1.05	0.236
LamProp (MFI)	0.95	0.91–0.99	**0.017**
Lumen (MFI)	1.02	1.01–1.03	**0.002**
Apex/base ratio	10.6	1.71–66.0	**0.011**
Middle/base ratio	77.3	0.21–28,302	0.149
Apex/middle ratio	19.1	2.10–173	**0.009**
Slope	1.05	1.01–1.10	**0.013**
CLS	1.04	1.01–1.06	**0.006**
Gap score	3.00	0.717–12.529	0.133

Statistical analysis: univariable Cox regression analysis of factors associated with adverse LRE in the Vienna cohort: first/further decompensation, ACLF, and liver-related death (composite endpoint; n = 10 events). *p*-values below 0.05 are indicated in bold. CLS, Confocal Leak score; HR, hazard ratio; HVPG, hepatic venous pressure gradient; LRE, liver-related events; MELD, model for end-stage liver disease; MFI, mean fluorescence intensity.

Importantly, established prognostic factors such as MELD (HR per point: 1.26, 1.12–1.41, *p* <0.001), albumin (HR per g/L: 0.87, 0.78–0.96, *p* = 0.006), and HVPG (HR per point: 1.13, 0.98-1.30, *p* = 0.081) were also linked to adverse outcomes in Cox regression models, supporting the plausibility and representativeness of CLE results. Multivariate models or subgroup analyses were not performed because of sample size considerations to avoid overfitting.

### Interobserver variability of CLE readouts

To address interobserver reliability, images were independently analyzed by two blinded observers in the Bern and the Vienna cohorts, respectively. Interobserver variability was substantially higher for the qualitative scoring systems compared with the new quantitative 3-line/3-compartment approaches in both cohorts (Fig. 6). Among all assessed methods, the 3-line/3-compartment approach exhibited the best interobserver agreements, underscoring robustness and reproducibility. For example, the 3-line method showed excellent interobserver agreement in the Vienna cohort, including base (ICC = 0.95, 95% CI 0.91–0.97), middle (ICC = 0.97, 95% CI 0.95–0.98), and apex (ICC = 0.97, 95% CI 0.94–0.98).

**Fig. 6:**
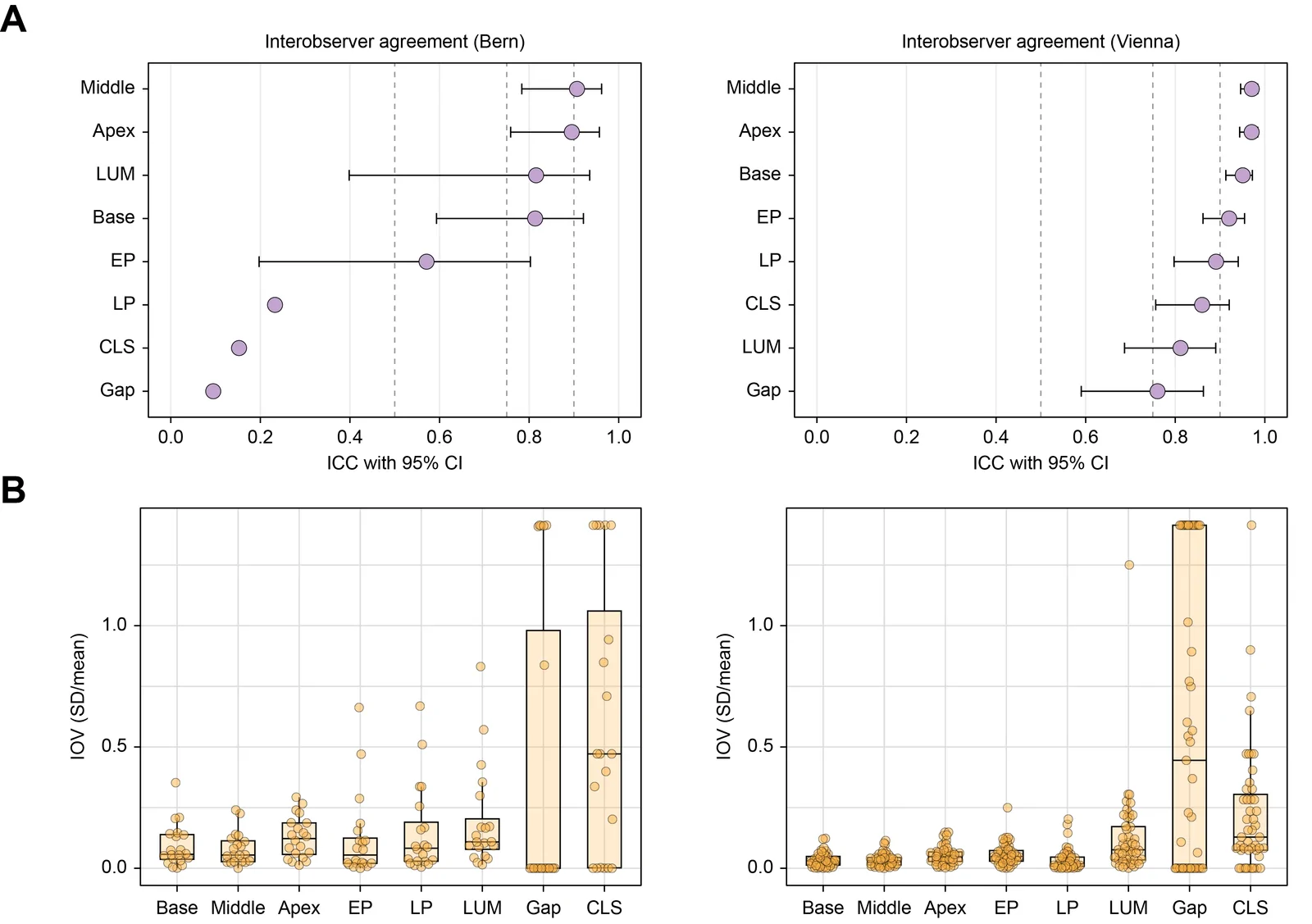
Interobserver agreement and variability of CLE readouts. (A) Interobserver agreement was evaluated using ICC(2,1) (two-way random-effects, absolute agreement); error bars indicate 95% CIs. (B) Standard deviation was divided by the mean of two different reader evaluations. CLE, confocal laser endomicroscopy; CLS, Confocal Leak Score; EP, epithelium; Gap, Gap score; ICC, intraclass correlation coefficient; IOV, interobserver variability; LP, lamina propria; LUM, lumen.

## Discussion

The present study shows that CLE enables direct, *in vivo*, high-resolution visualization of intestinal barrier dysfunction in patients with ACLD, and that quantitative CLE-derived measures indicating elevated permeability increase across disease stages. In exploratory analyses, these measures were additionally associated with portal hypertension markers, liver function, and liver-related outcomes. By integrating two independent cohorts from Bern and Vienna and introducing a novel quantitative analytical framework based on 3-line and 3-compartment analysis, this study extends previous CLE work in other diseases,[10], [11], [12], [13]^,^^21^ and in the context of cirrhosis,^22^ and supports the clinical relevance of intestinal barrier permeability as a key feature of gut–liver axis impairment in ACLD.^23^^,^^24^

The gut–liver axis is now recognized as a key driver of cirrhosis progression, where intestinal barrier dysfunction promotes microbial translocation, systemic inflammation, and thereby predisposes to clinical complications such as hepatic decompensation, ACLF, and liver-related death.[25], [26], [27], [28] The present results reinforce this concept by demonstrating that CLE-derived measures of intestinal permeability correlate closely with established markers of portal hypertension and liver dysfunction, including HVPG, albumin, MELD, and systemic inflammation biomarkers. Importantly, significant associations were consistently observed across two independent cohorts, underscoring the external validity and reproducibility of the findings. For example, 3-line parameters were robustly linked to disease stages, and key correlation results were validated, for example, the link between apex/base ratio and albumin, and surrogates for portal hypertension (HVPG/PLT count). Amelioration of portal hypertension may beneficially impact on intestinal (vascular) permeability via decreasing hydrostatic pressure within the splanchnic vasculature – a hypothesis that is also supported by observations that systemic inflammation ameliorates after transjugular intrahepatic portosystemic shunt.^29^

A major methodological advance of the present study is the implementation of a quantitative 3-line and 3-compartment CLE analysis. While earlier CLE-based approaches relied predominantly on qualitative or semi-quantitative grading of fluorescein leakage, the current framework enables a more granular and objective characterization of mucosal permeability across distinct anatomical layers. This quantitative read-out not only follows established methods of patient stratification (CTP stages, Baveno clinical stages, MELD, etc.), but also demonstrates stronger correlations with pathomechanistic biomarkers compared with conventional CLE scoring. The results indicate that the quantitative CLE metrics are robust and reproducible. Whether they are suited for other conditions associated with altered intestinal permeability remains to be tested in future studies. In its current form the quantitative analysis is performed offline and manually, which together with probe cost and the need for an experienced reader, currently limits its use to specialized research settings. Nevertheless, the strong interobserver agreement of the quantitative metrics suggests they are well suited for standardization and, if (semi-)automated image analysis would become available in the future, could be extended to broader application.

While CLE has been extensively studied as a tool to detect intestinal leakage in IBD and functional gastrointestinal disorders,[10], [11], [12], [13]^,^^21^ its application in ACLD has remained largely unexplored. In the present study, we observed a high prevalence of intestinal barrier dysfunction in ACLD, with overt fluorescein leakage into the gut lumen being present in approximately half of the patient cohort; importantly, being much more frequent in advanced (decompensated) cirrhosis stages. These findings are in line with accumulating evidence linking increased intestinal permeability to advanced portal hypertension and cirrhosis-related complications.^30^

Beyond cross-sectional associations, CLE-based assessment of intestinal barrier dysfunction showed prognostic relevance. Patients with severe CLE-derived permeability alterations experienced a significantly higher rate of adverse LRE, consistent with established prognostic models such as MELD or CTP scores. Besides binary classifications stratifying patients by risk of disease progression, the novel quantitative readouts showed proportional increase of hazard for disease progression with quantitative CLE variables, further emphasizing their clinical relevance. Notably, we observed an opposing trend of lamina propria and lumen fluorescence intensity regarding risk of disease progression in Cox regression analyses, likely representing a shift of fluorescein from the lamina propria to the gut lumen – in combination with apex/base ratio and slopes from the 3-line analysis – which emphasizes the plausibility of observed results. These exploratory observations suggest that CLE-derived metrics may carry prognostic information. However, given the limited number of events and the absence of multivariable adjustment, they should be regarded as hypothesis-generating rather than evidence of prognostic value independent of established markers such as MELD or HVPG.

Numerous experimental studies have deciphered mechanisms leading to intestinal barrier dysfunction in cirrhosis.[31], [32], [33], [34], [35], [36], [37], [38] Current human studies investigating intestinal barrier dysfunction in ACLD have largely relied on indirect biomarkers such as circulating PAMPs, inflammatory mediators,[39], [40], [41] sugar absorption tests, or radioisotopes.^42^^,^^43^ While informative, these surrogate markers often do not provide direct or quantitative insights into mucosal barrier permeability. Biopsy-based approaches (*e.g.* transepithelial electrical resistance and transcriptomic profiling^44^^,^^45^) enable mechanistic characterization but remain confined to highly specialized research settings. In contrast, CLE offers a direct, *in vivo* functional assessment during routine endoscopy, with quantitative analysis performed offline, representing a promising technique for translational research and clinical applications. Notably, CLE readouts predominantly reflect the paracellular/transepithelial permeability of the mucosa to a small solute (fluorescein, ∼330 Da). The stepwise apex/base gradient and the redistribution of fluorescence from the lamina propria to the lumen are consistent with loss of tight-junction integrity and increased convective transfer, in line with the observed correlations with albumin and HVPG. Thus, CLE should be regarded as a complementary functional read-out *in vivo* rather than competing with techniques dissecting molecular mechanisms.

Although the ileum is traditionally considered the primary site of bacterial translocation,^4^^,^^9^^,^^46^ our data suggest that CLE assessment of the small intestinal mucosa in the duodenum also yields clinically relevant information on intestinal barrier dysfunction in ACLD. Given that patients with ACLD commonly undergo routine upper gastrointestinal endoscopy for variceal screening or treatment according to current guidelines,^14^^,^^16^ CLE could be integrated into existing workflows. This contrasts with colonoscopy-based CLE, as colonoscopy may be less frequently performed in ACLD but more commonly in IBD.^47^

From a broader pathophysiological perspective, the direct visualization of (intravenously injected) fluorescein leakage into the intestinal lumen by CLE may also revive earlier concepts linking chronic liver disease to intestinal protein loss, such as increased fecal albumin excretion described decades ago.^48^ Recent experimental and translational studies have revisited these mechanisms, further supporting a central role of intestinal barrier dysfunction in ACLD progression.^49^^,^^50^ In this context, the strong correlations between apex/base ratio and albumin (negative correlation) and HVPG (positive correlation) should be noted and further investigated in future studies.

The present study has notable strengths. First, the inclusion of two independent cohorts, two independent readers for each center, and a strong interobserver agreement for the novel analysis methods enhances generalizability and preemptively addresses concerns of reader/center-specific bias. Second, the introduction of a quantitative, compartment-based CLE analysis represents a substantial methodological advance with suitable interobserver reproducibility. Third, the approach yields objective metrics that are closely linked to clinically and pathophysiologically relevant parameters, supporting their translational relevance.

Nevertheless, we acknowledge limitations. First, despite the inclusion of two independent cohorts, sample size remains moderate, particularly for subgroup and outcome analyses. Larger prospective studies are required to confirm these findings, to refine prognostic cut-offs, and to evaluate whether CLE-based barrier assessment adds incremental value beyond established risk models. Second, CLE readouts were not adjusted for medications that may influence intestinal permeability and co-vary with disease severity (*e.g.* non-selective beta-blockers, rifaximin) because of the limited sample size. Finally, the correlation between CLE results and certain biomarkers including HVPG could not be cross-validated in the Bern cohort, as a result of different clinical work-up between centers.

Future studies could determine whether therapeutic interventions directed at gut barrier function translate into improved clinical outcomes; and investigate if CLE results correlate with molecular readouts from mucosa biopsies such as tight-junction expression and immune cell activation. We note that for the latter point, no established treatment improving gut barrier integrity in ACLD is yet approved for clinical practice, but should be considered for future clinical trials. For example, despite challenges in clinical implementation,^51^ pharmacological agents targeting BA signaling improved intestinal barrier integrity and ameliorated chronic liver disease in experimental studies.^31^

In summary, CLE enables direct, *in vivo* visualization and quantitative assessment of intestinal barrier dysfunction in patients with ACLD. The newly introduced 3-line and 3-compartment analyses provide robust, reproducible metrics that closely reflect disease severity, portal hypertension, and clinical outcomes. Further prospective and translational studies are warranted to validate findings, link CLE to molecular mechanisms of gut barrier integrity, and explore further interventional strategies targeting the gut–liver axis in cirrhosis.

## Abbreviations

ACLD, advanced chronic liver disease; ACLF, acute-on-chronic liver failure; ALD, alcohol-related liver disease; BA, bile acid; cACLD, compensated ACLD; CLE, confocal laser endomicroscopy; CLS, Confocal Leak score; CRP, C-reactive protein; CTP, Child-Turcotte-Pugh; dACLD, decompensated ACLD; FI, fluorescence intensity; HE, hepatic encephalopathy; HR, hazard ratio; HRS-AKI, hepatorenal syndrome–acute kidney injury; HVPG, hepatic venous pressure gradient; IBD, inflammatory bowel disease; ICC, intraclass correlation coefficient; ISO, International Organization for Standardization; LRD, liver-related death; LRE, liver-related event; MASLD, metabolic dysfunction-associated liver disease; MELD, model for end-stage liver disease; MFI, mean fluorescence intensity; PAMPs, pathogen-associated molecular patterns; PC1, principal component 1; PC2, principal component 2; PCA, principal component analysis; PLT, platelet count; RFI, relative fluorescence intensity; SBP, spontaneous bacterial peritonitis.

## Authors’ contributions

Study concept and design: MS, RW, JH, BS, TR. Data acquisition: all authors. Data analysis: MS, RW, JH, BS, TR. Data interpretation: all authors. Drafted the manuscript: BS, MS. All authors of the manuscript: JS, MF, JC, SGR, MM, AB, MT, TR, JH, RW. Approved the final version of the manuscript: all authors.

## Data availability

Data are available at reasonable request to the corresponding author.

## Declaration of generative AI and AI-assisted technologies in the writing process

During the preparation of this work the authors used ChatGPT 5.0 (OpenAI) to maximize readability, eloquence and grammatical correctness. After using this tool, the authors reviewed and edited the content as needed and take full responsibility for the content of the publication.

## Financial support

The study was supported by the Clinical Research Group MOTION, Medical University of Vienna, Vienna, Austria – a project funded by the 10.13039/501100016168Clinical Research Groups Programme of the Ludwig Boltzmann Gesellschaft (Grant Nr: LBG_KFG_22_32) with funds from the Fonds Zukunft Österreich.

## Conflicts of interest

BS has received travel support from AbbVie, Gilead, Falk, and Mirum.

MM served as a speaker and/or consultant and/or advisory board member for AbbVie, AstraZeneca, Echosens, Eli Lilly, Gilead, Ipsen, Takeda, and W. L. Gore & Associates and received grant support from Echosens as well as travel support from AbbVie and Gilead. MT received grant support from Albireo, Alnylam, Cymabay, Falk, Genentech, Gilead, Intercept, MSD, Takeda, and UltraGenyx, honoraria for consulting from AbbVie, Albireo, Agomab, Boehringer Ingelheim, BiomX, Chemomab, Dexoligo Therapeutics, Falk, Genfit, Gilead, GSK, Hightide, Intercept, Ipsen, Janssen, MSD, Novartis, Phenex, Pliant, Regulus, Siemens, and Shire, speaker fees from Albireo, Boehringer Ingelheim, Bristol-Myers Squibb, Falk, Gilead, Ipsen, Intercept, MSD, and Madrigal, as well as travel support from AbbVie, Falk, Gilead, Janssen, and Intercept. He is also co-inventor of patents on the medical use of 24-norursodeoxycholic acid filed by the Medical University of Graz. TR received grant support from Abbvie, Boehringer Ingelheim, Gilead, Intercept/Advanz Pharma, MSD, Myr Pharmaceuticals, Philips Healthcare, Pliant, Siemens, and W. L. Gore & Associates; speaking honoraria from Abbvie, Echosens, Gilead, Intercept/Advanz Pharma, Roche, MSD, and W. L. Gore & Associates; consulting/advisory board fee from Abbvie, Astra Zeneca, Bayer, Boehringer Ingelheim, Gilead, Intercept/Advanz Pharma, MSD, Resolution Therapeutics, and Siemens; and travel support from Abbvie, Boehringer Ingelheim, Dr. Falk Pharma, Gilead, and Roche. The remaining authors declare no conflict of interest.

Please refer to the accompanying ICMJE disclosure forms for further details.
