# Supplementary material for: Intestinal barrier dysfunction assessed by confocal laser endomicroscopy correlates with cirrhosis severity and clinical outcomes
Source: JHEP Rep. 2026 Sep 18;8(12):102037. doi: 10.1016/j.jhepr.2026.102037 (PMC13756262; doi:10.1016/j.jhepr.2026.102037)
Supplement: Supplementary file 1 [file mmc1.pdf]

Intestinal barrier dysfunction assessed by confocal laser endomicroscopy correlates with cirrhosis severity and clinical outcomes

Benedikt Simbrunner, Marcel Sorribas, Jessica Schaerer, Marco Felber, Jordi Codinach, Susana Gomes Rodrigues, Mattias Mandorfer, Annalisa Berzigotti, Michael Trauner, Thomas Reiberger, Johann Hammer, Reiner Wiest

Table of contents

Supplementary methods..2
Supplementary results..4
Supplementary figures... 5
Supplementary tables...18
Supplementary references... 21

Supplementary methods

3-line method

Fluorescence intensity (FI, "grey value") within the epithelial layer was measured at three predefined levels along the epithelial lining – base, middle, and apex - using Image J **(Figure-2B)**. For each line, pixel-wise grey values were averaged to obtain the mean fluorescence intensity (MFI). The relative fluorescence intensity (RFI) of each epithelial level was calculated as the proportion of its MFI relative to the sum of MFIs across all three lines which was defined as 100%. Ratios between the three epithelial levels were calculated based on the assumption that the amount of fluorescein determines fluorescent intensity as it permeates from the basal to the apical epithelial layers. Accordingly, a higher apex-to-base ratio was interpreted as an indirect marker of increased fluorescein permeation across the epithelium, reflecting epithelial permeability in the assessed segment. In addition, fluorescence intensity slopes ('3-line slopes') were calculated for each subject by fitting a linear regression across the three anatomical layers (base, middle, apex) using their respective MFIs. The resulting regression coefficient was used as a quantitative measure of the FI slope.

Confocal leak score (CLS)

CLS was assessed as previously described by Chang et al [1]. The CLS scale ranges from 0 (representing no intestinal permeability) to 100 (representing complete loss of barrier function). Accordingly, three features were assessed **(Fig. S12)**: **(A)** cell junction enhancement (demonstrating fluorescein enhancement between epithelial cells assumed to be due to loss of inter-cellular connectivity, e.g. diminished tight junctions (TJ)/function), **(B)** fluorescein leak (an outflow of fluorescein from the submucosal space into the lumen through epithelial breaks), and **(C)** cell drop out (active shedding of one or multiple enterocytes leading to an epithelial gap). Each image was evaluated for these features, and the CLS was determined by dividing the number of images containing one or more of the features by the total number of images per patient, then multiplying that number by 100. This resulted in a score ranging from 0 (representing no barrier dysfunction) to 100 (representing complete barrier dysfunction).

Gap score

Epithelial gap density was determined as previously described [2]. In brief, the gap density was derived by normalizing the total number of epithelial gaps (as exemplified in **Fig. S12D**) per 1000 epithelial cells counted. In this study the perimeter of the epithelial layer was used as a surrogate marker for the number of epithelial cells.

Statistical analysis

Categorical variables are reported as absolute (n) and relative (%) frequencies, while continuous variables are displayed as mean ± standard error of the mean or median with interquartile range (IQR), as appropriate. Group comparisons of categorical variables were performed using Chi squared or Fisher's exact test.

Principal component analysis (PCA) was performed using the 'FactoMineR' package, with centered and scaled variables. PCA biplots, scree plots, variable contribution plots, and individual factor maps were generated ('factoextra' package). Patients were additionally visualized according to disease stage using color-coded individual factor maps with confidence ellipses.

The level of significance was set at a two-sided p-value <0.05 for all analyses.

Supplementary results

CLE results - Bern cohort

To compare the new quantitative CLE readouts against previously established scores, **CLS** and **Gap Score (Fig. S3)** were assessed. The CLS demonstrated a stepwise numerical increase from healthy controls to patients with more advanced cirrhosis, with mean CLS values rising from 9.41 ± 8.54 in controls to 20.3 ± 8.97 in CTP-A, 20.0 ± 15.1 in CTP-B, and 25.6 ± 7.27 in CTP-C (p=0.184). The Gap Score remained within the reported normal range in healthy controls and showed few increased scores in patients with ACLD (p=0.595).

PCA was performed to assess the relative contribution of the different parameters to the overall variance. The analysis incorporated clinical and laboratory markers, CLS and Gap Score, as well as 3-line/-compartment CLE parameters. The PCA yielded two principal components explaining 59% of the total variance, with Dimensional Axis 1 (PC1) accounting for 39.9% and Dimensional Axis 2 (PC2) for 18.6% of the variability **(Figure-3C; Fig. S4)**. Healthy controls formed a clearly distinct cluster, indicating strong separation from patients with ACLD. In contrast, patients with ACLD exhibited closer spatial proximity but showed separation according to disease severity (CTP stages). Analysis of variable loadings revealed that novel CLE parameters were strong contributors to PC1 and PC2. Specifically, 5 of the 8 variables with the highest loadings on PC1 originated from 3-line/-compartment analyses, including the apex/base ratio. Similarly, 4 of the 5 top contributors to PC2 were derived from CLE readouts, underscoring their relevance in capturing variance related to intestinal barrier dysfunction in cirrhosis.

CLE results - Vienna cohort

PCA was performed in the Vienna cohort to explore multivariate patterns across clinical variables stratified by Baveno clinical stages **(Fig. S10)** [3]. The first two principal components explained 53.8% of the total variance, with PC1 accounting for 33.6% and PC2 for 20.2%. PC1 was predominantly driven by variables derived from the novel CLE-based measurement technique, with 6 of the 9 highest contributing variables originating from CLE parameters. PC2 was likewise strongly determined by CLE-derived variables, with 3 of the 5 strongest contributors originating from the new 3-line/-compartment approach. In the PCA score plot, patients demonstrated separation along PC1 and PC2 according to Baveno clinical stages, indicating that CLE-derived parameters capture clinically relevant variance associated with clinical disease stages.

Supplementary figures

Fig. S1. Quantitative epithelial 3-line analysis (Bern cohort): (A) comparison of base-middle-apex, (B/C) comparison between disease stages.

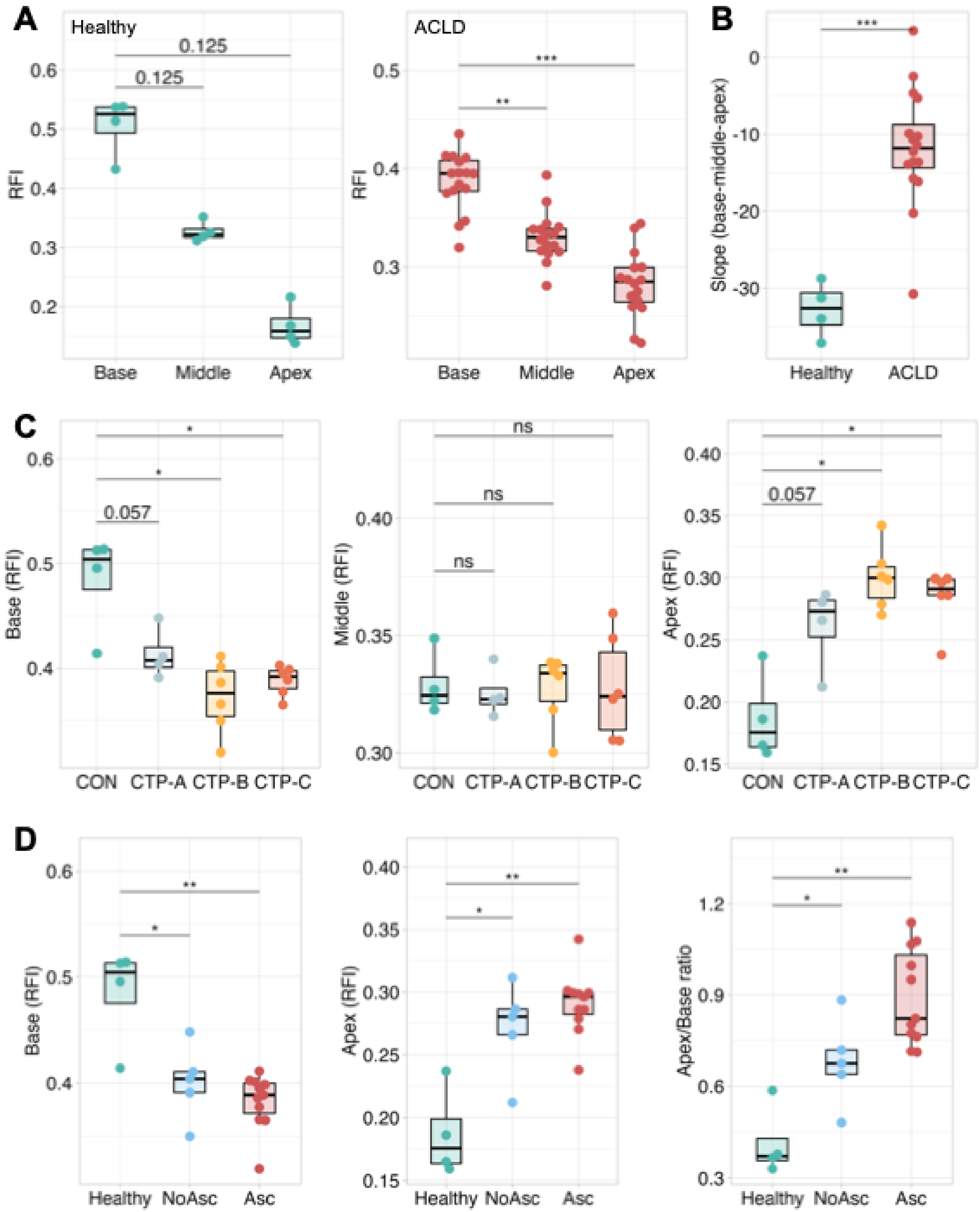

Statistics: Group comparisons were performed using pairwise Wilcoxon rank-sum tests versus the first group (Base; Healthy, CON) with Benjamini–Hochberg correction for multiple testing. Figure legend: (ns) not significant; (*) p<0.05; (**) p<0.01; (***) p<0.001. Abbreviations: (RFI) relative fluorescence intensity; (NoAsc) no ascites; (Asc) ascites; (CON) healthy controls, (CTP) Child-Turcotte-Pugh stage.

Fig. S2. Quantitative epithelial 3-compartment analysis (Bern cohort): (A) RFI between 3 compartments, (B/C) comparison between disease stages.

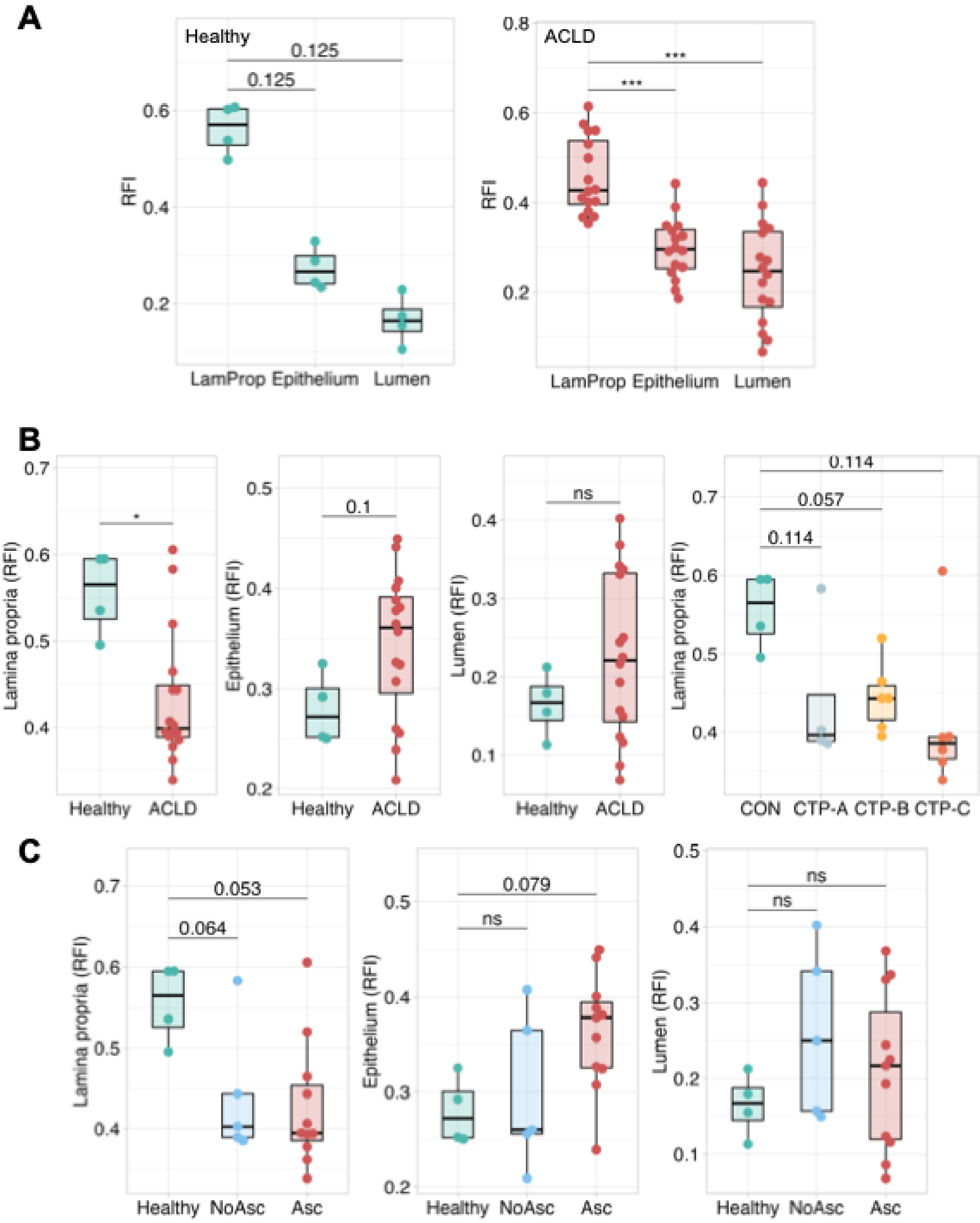

Statistics: Group comparisons were performed using pairwise Wilcoxon rank-sum tests versus the first group (Base; Healthy, CON) with Benjamini–Hochberg correction for multiple testing. Figure legend: (ns) not significant; (*) $p<0.05$; (**) $p<0.01$; (***) $p<0.001$. Abbreviations: (ACLD) advanced chronic liver disease; (RFI) relative fluorescence intensity; (NoAsc) no ascites; (Asc) ascites; (CON) healthy controls, (CTP) Child-Turcotte-Pugh stage.

Fig. S3. Gap and Confocal Leak Score (Bern cohort): comparison between healthy controls and patients with ACLD.

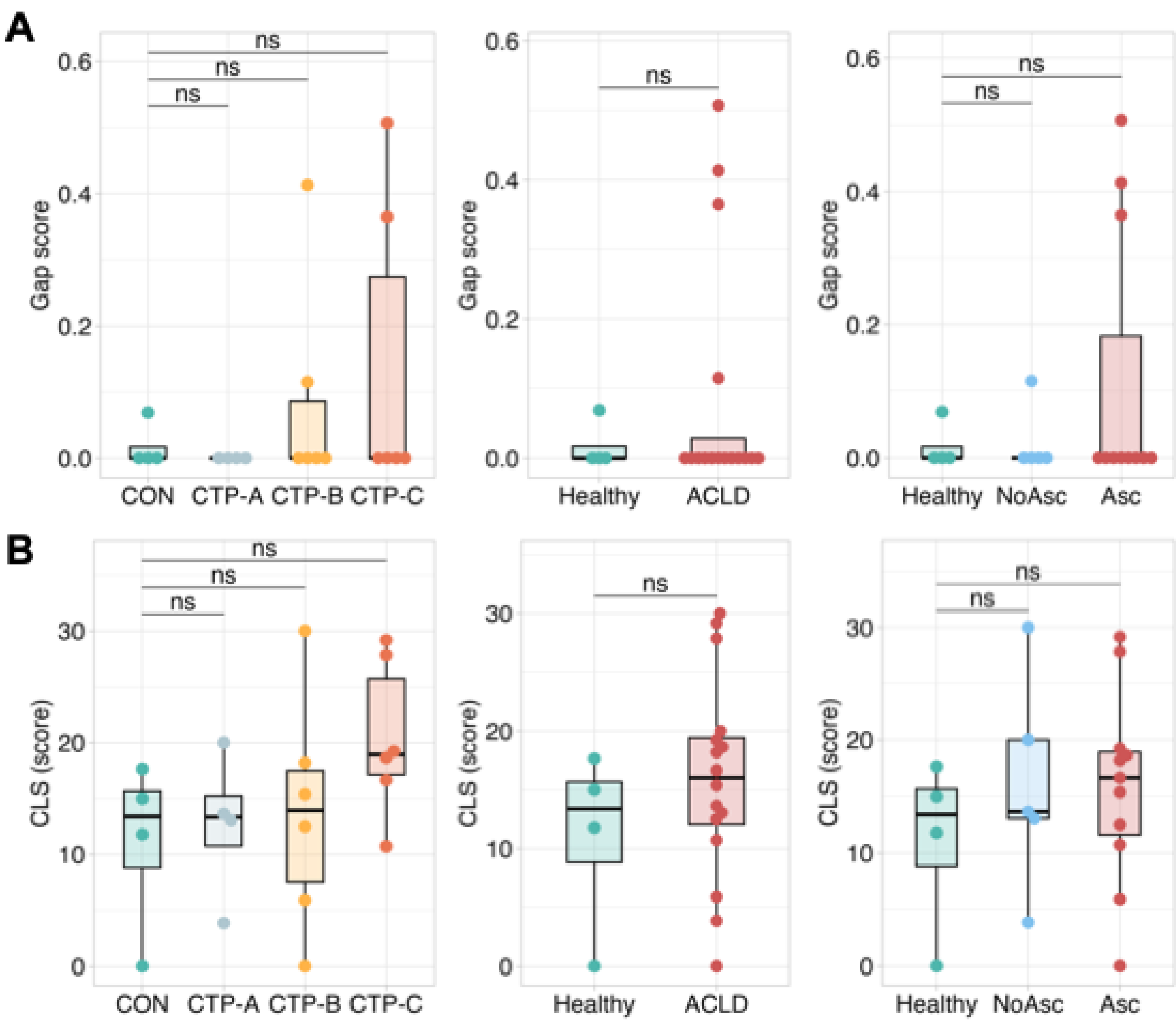

Statistics: Group comparisons were performed using pairwise Wilcoxon rank-sum tests versus the first group (Healthy controls) with Benjamini–Hochberg correction for multiple testing. Figure legend: (ns) not significant; (*) p<0.05; (**) p<0.01; (***) p<0.001. Abbreviations: (ACLD) advanced chronic liver disease; (CLS) confocal leak score; (CON) healthy controls, (CTP) Child-Turcotte-Pugh stage; (Asc) ascites.

Fig. S4. Principal component analysis: variance explained, biplot, and variable contributions (Bern cohort).

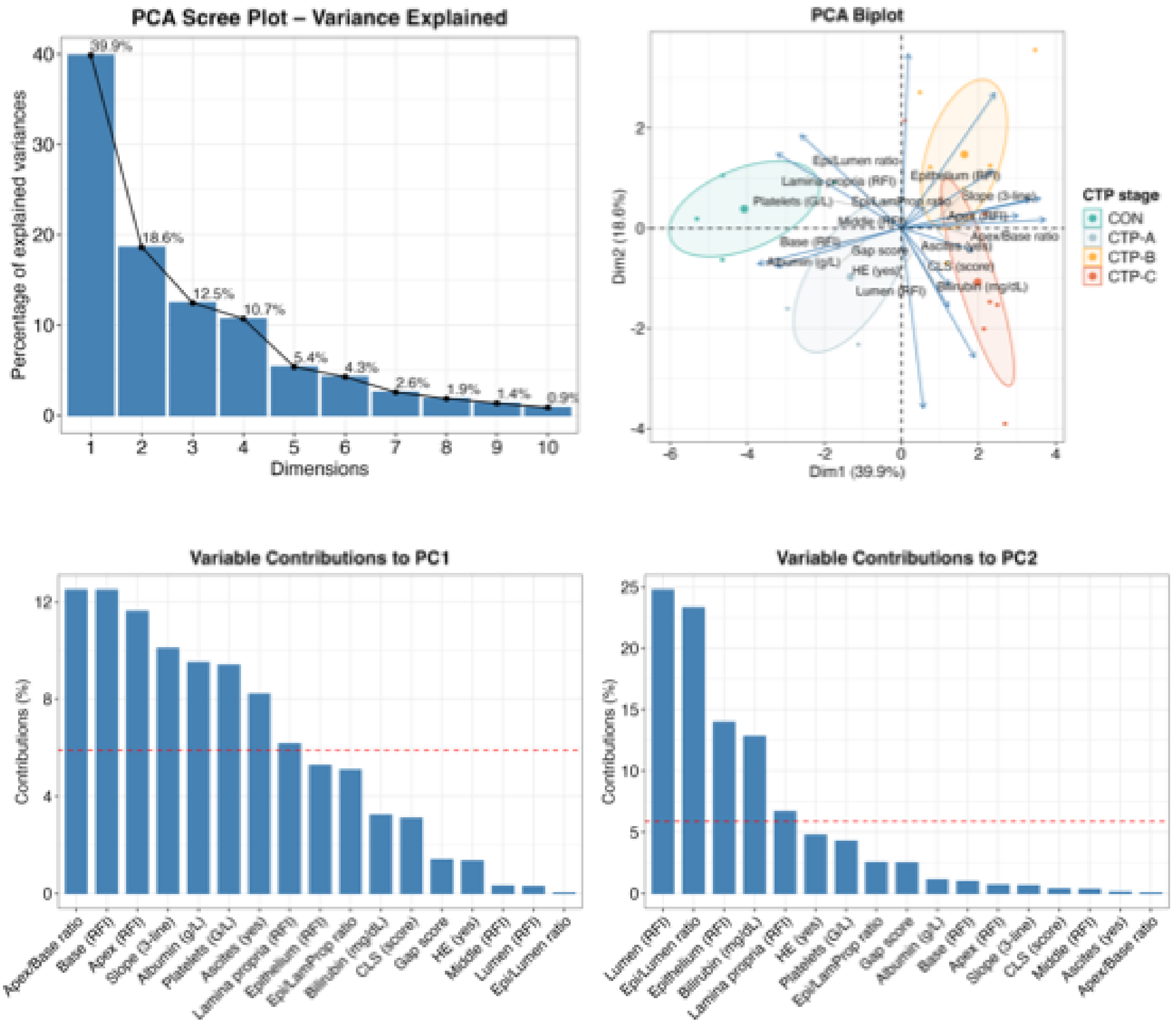

Statistics and Legend: PCA was conducted using a correlation matrix with unit variance scaling; all plots are based on complete-case observations only. Panel 1: Scree plot showing the proportion of variance explained by each principal component. Panel 2: PCA biplot illustrating the joint projection of individuals and variables onto the first two principal components. Individuals are colored by either healthy controls “CON” or patients “CTP stage” with 95% confidence ellipses, while arrows represent variable loadings. Panels 3-4: Bar plots depicting the relative contributions of individual variables to the first (PC1) and second (PC2) principal components. Abbreviations: (RFI) relative fluorescence intensity; (CLS) confocal leak score; (LamProp) lamina propria; (HE) hepatic encephalopathy; (CON) healthy controls, (CTP) Child-Turcotte-Pugh stage.

Fig. S5. Presence of fluorescein leakage in patients stratified by Baveno clinical stages: cACLD, first decompensation, further decompensation (Vienna cohort).

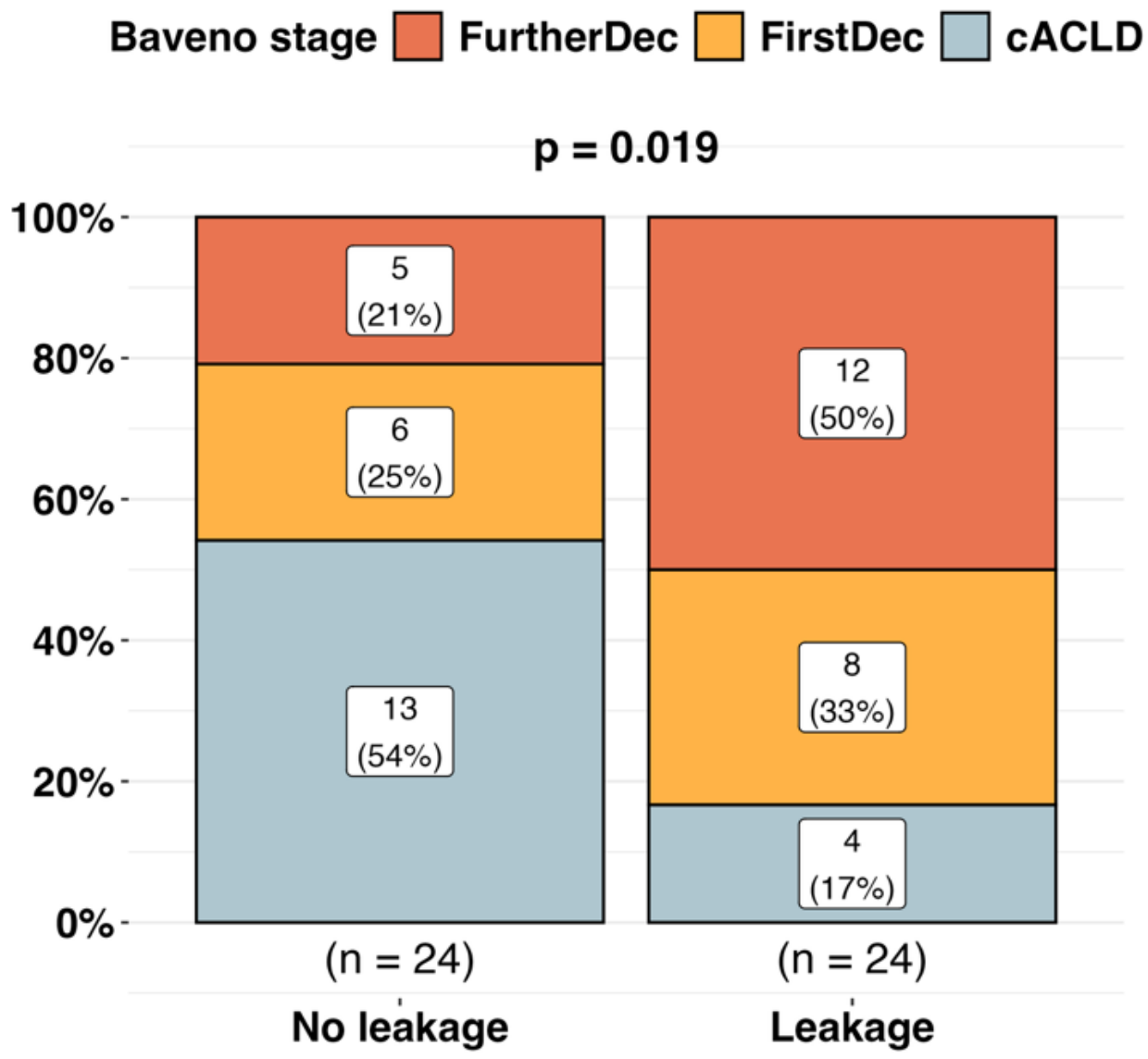

Statistics: Group comparison was performed by Chi squared test. Abbreviations: (cACLD) compensated advanced chronic liver disease, (FirstDec) first hepatic decompensation, (FurtherDec) further hepatic decompensation.

Fig. S6. CLE readouts in patients stratified by (A) CTP stages and (B) Baveno clinical stages: cACLD, first decompensation, further decompensation (Vienna cohort).

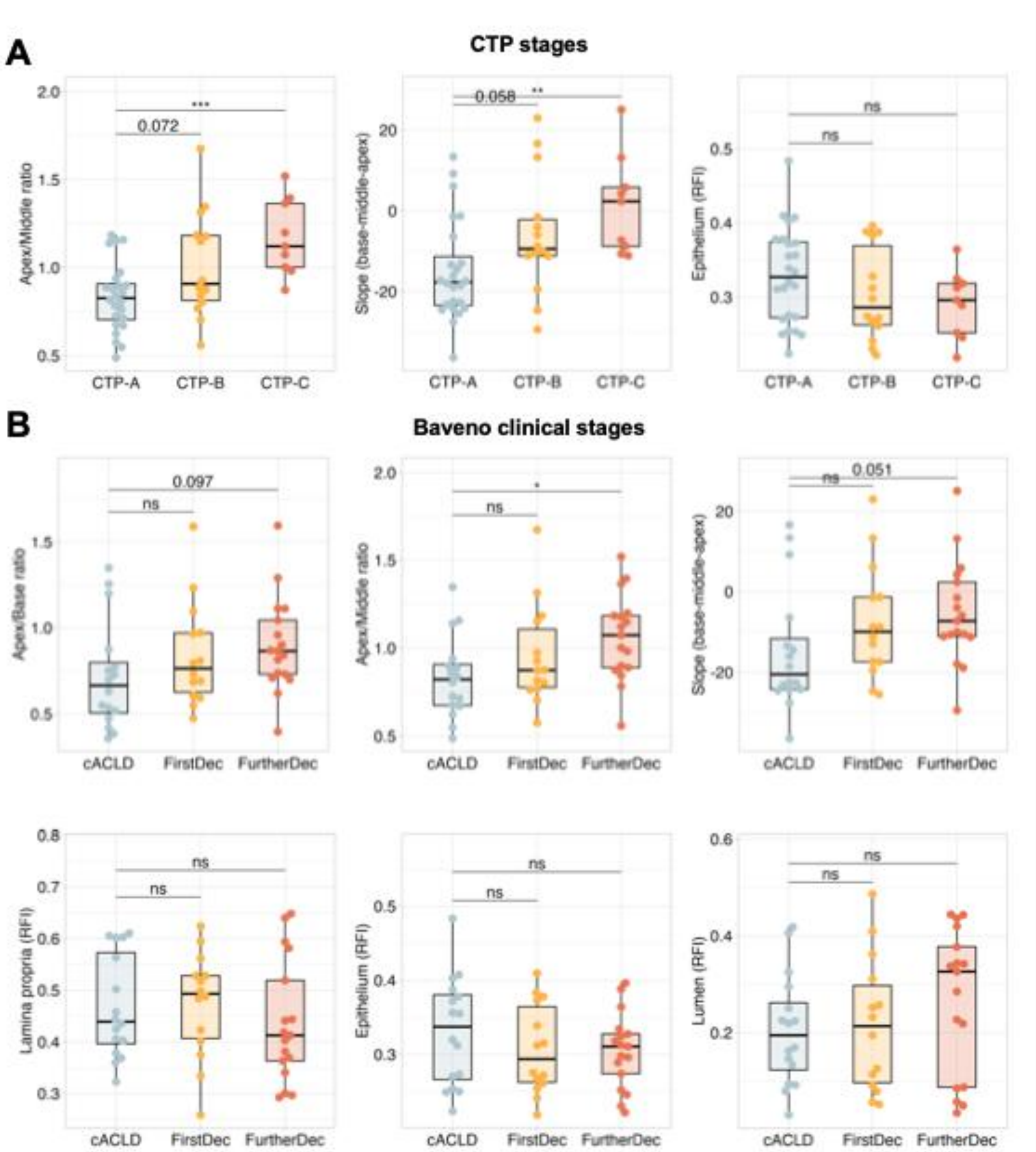

Statistics: Group comparisons were performed using pairwise Wilcoxon rank-sum tests versus the CTP-A/cACLD group with Benjamini–Hochberg correction for multiple testing. Figure legend: (ns) not significant; (*) p<0.05; (**) p<0.01; (***) p<0.001. Abbreviations: (CTP) Child-Turcotte-Pugh, (cACLD) compensated advanced chronic liver disease, (FirstDec) first hepatic decompensation, (FurtherDec) further hepatic decompensation, (RFI) relative fluorescence intensity, (CLE) confocal laser endomicroscopy.

Fig. S7. CLE readouts in patients stratified by the presence of ascites (Vienna cohort).

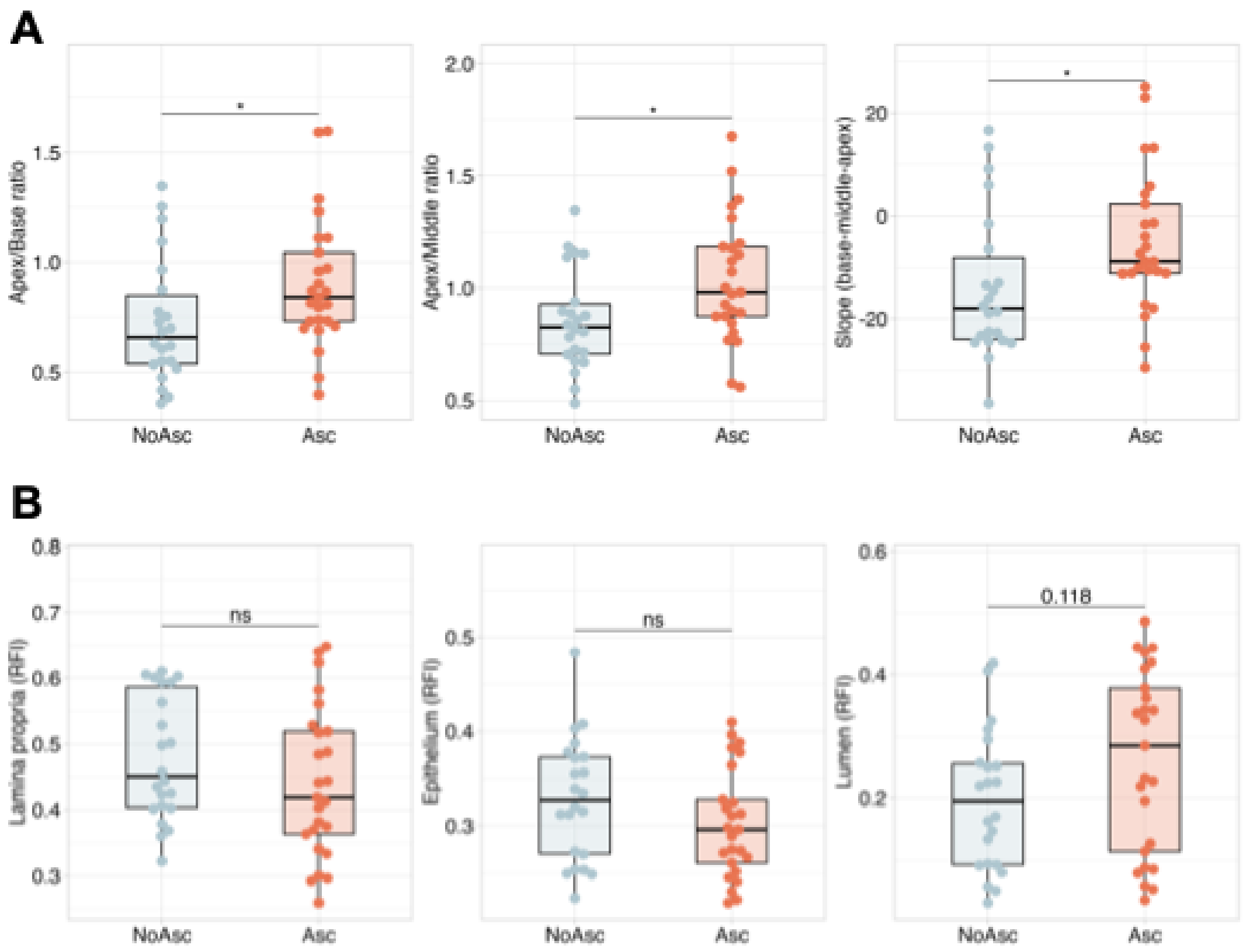

Statistics: Group comparisons were performed using two-sided Wilcoxon rank-sum tests. Figure legend: (ns) not significant; (*) p<0.05; (**) p<0.01; (***) p<0.001. Abbreviations: (Asc) Ascites, (RFI) relative fluorescence intensity, (CLE) confocal laser endomicroscopy.

Fig. S8. Gap score and CLS compared by (A) CTP stages and (B) presence of ascites (Vienna cohort).

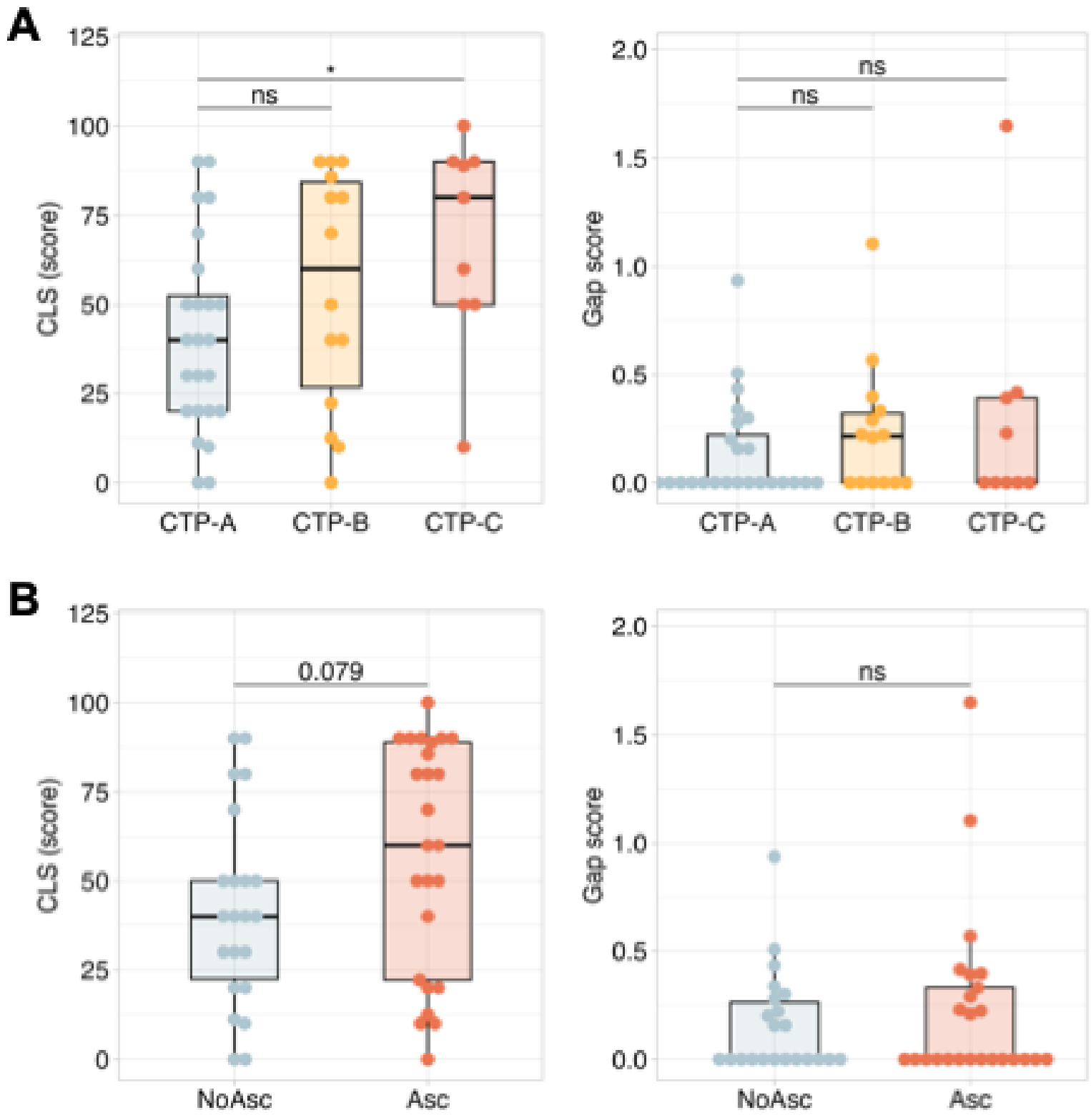

Statistics: Group comparisons were performed using pairwise Wilcoxon rank-sum tests with Benjamini–Hochberg correction for multiple testing. Figure legend: (ns) not significant; (*) p<0.05; (**) p<0.01; (***) p<0.001. Abbreviations: (Asc) ascites, (CTP) Child-Turcotte-Pugh stage, (CLS) Confocal Leak Score.

Fig. S9. Correlation between CLE readouts and markers of liver disease severity and inflammation (Vienna cohort).

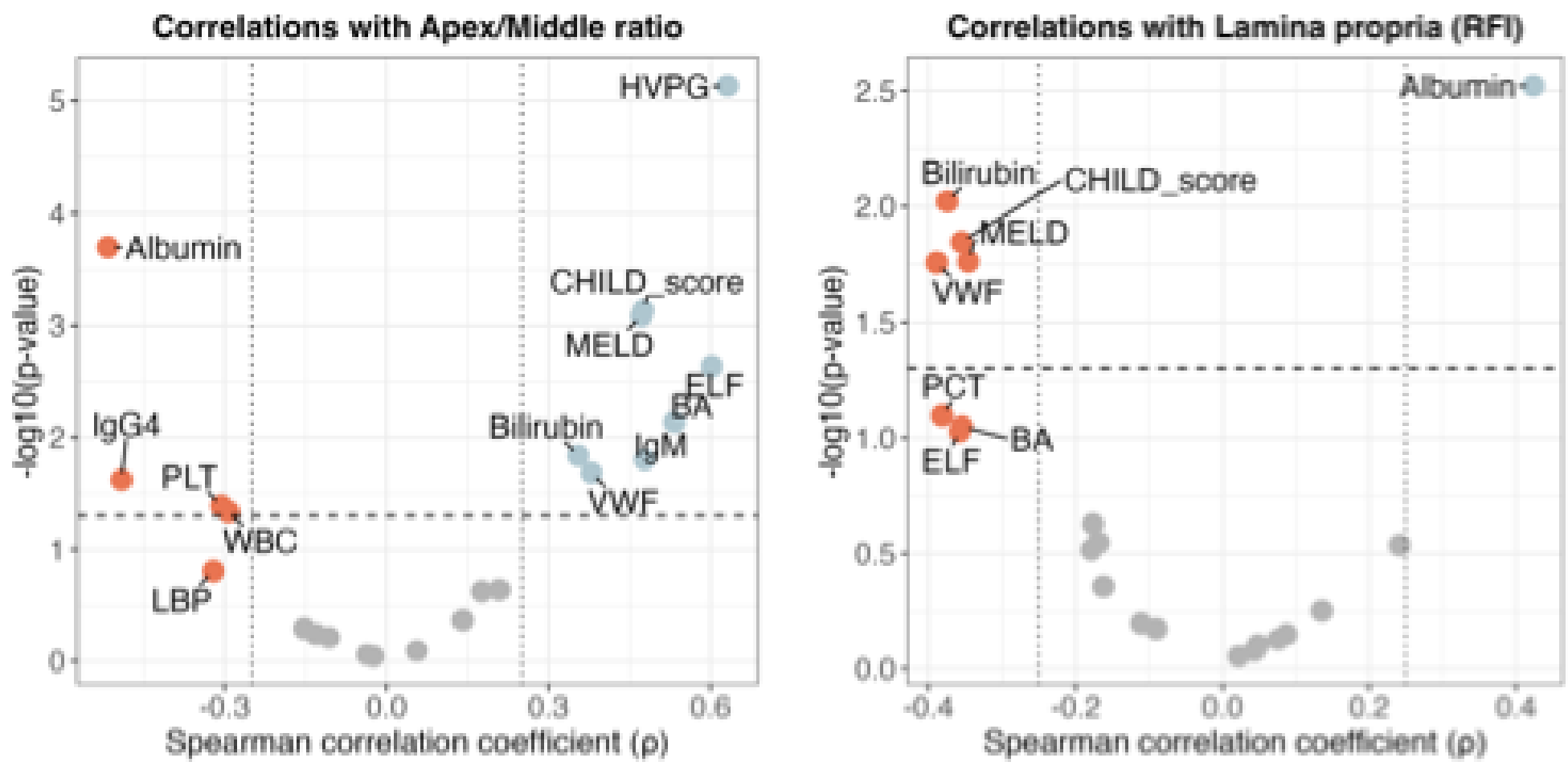

Statistics: Associations were assessed using two-sided Spearman rank correlation tests; p-values are shown without adjustment for multiple testing. Legend: Volcano plots depict Spearman rank correlations between imaging-derived metrics (dependent variables) and clinical and laboratory parameters. The x-axis shows the Spearman correlation coefficient (ρ), and the y-axis shows $-\log_{10}$(p-value). Horizontal dashed lines indicate nominal statistical significance ($p = 0.05$), and vertical dotted lines indicate effect size thresholds ($|\rho| = 0.25$). Points are color-coded by correlation strength and direction (positive correlations in blue, negative correlations in red, weak correlations in grey). Variables with $p < 0.05$ or $|\rho| > 0.25$ are labeled. Abbreviations: (CHILD) Child–Pugh score, (MELD) Model for End-Stage Liver Disease, (HVPG) Hepatic Venous Pressure Gradient, (PLT) platelet count, (VWF) von Willebrand factor, (CRP) C-Reactive Protein, (WBC) White Blood Cells, (PCT) Procalcitonin, (ELF) Enhanced Liver Fibrosis score, (BA) Bile acids, (Ig) Immunoglobulin.

Fig. S10. Principal component analysis: variance explained, biplot, and variable contributions (Vienna cohort).

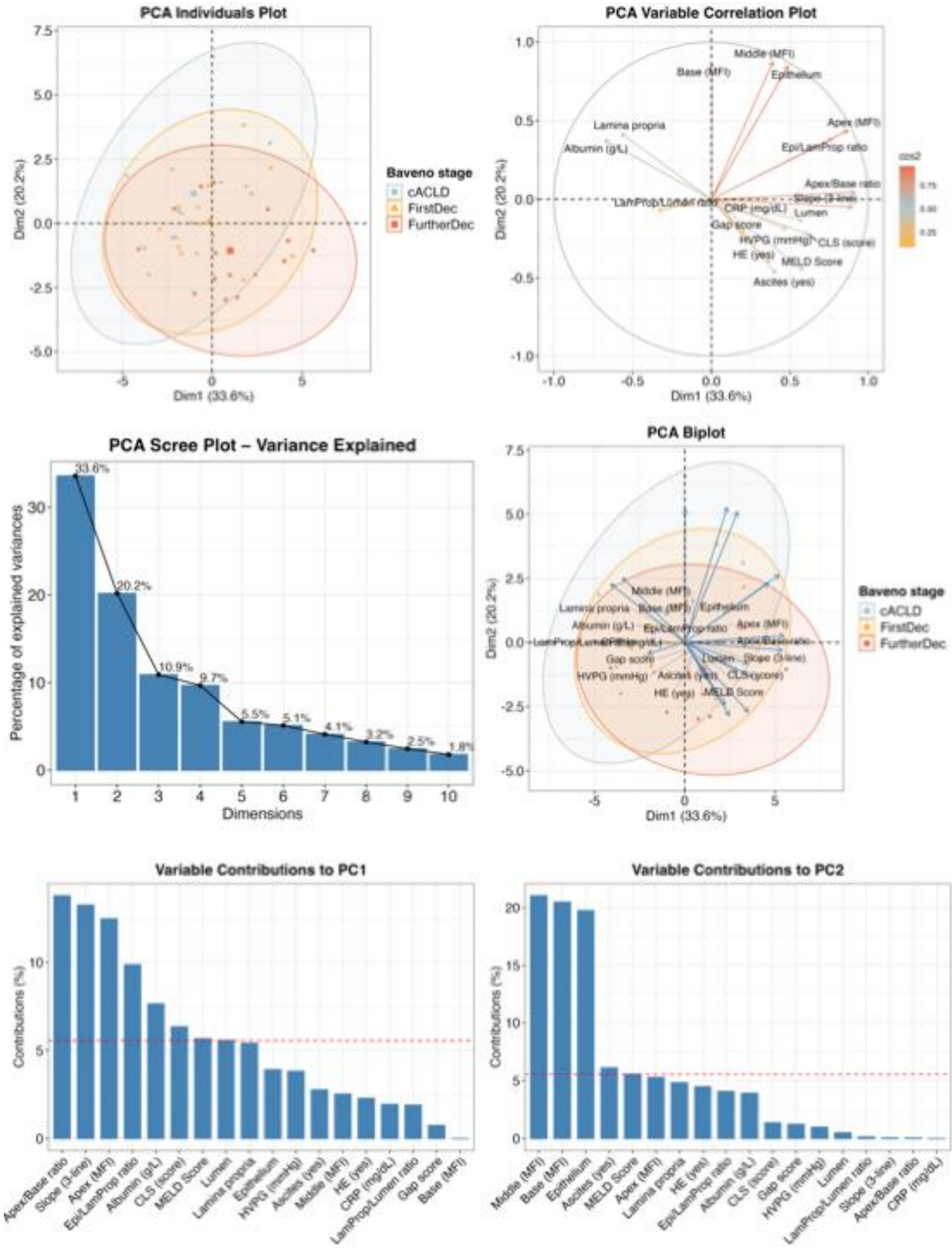

Statistics: PCA was conducted using a correlation matrix with unit variance scaling; all plots are based on complete-case observations. Panel 1: PCA plot illustrating the projection of individual patients onto the first two principal components, colored according to Baveno stage with 95% confidence ellipses. Panel 2: PCA variable correlation plot showing the relationships and loadings of variables across the first two principal components. Panel 3: Scree plot showing the proportion of variance explained by each principal component. Panel 4: PCA biplot illustrating the

joint projection of individuals and variables onto the first two principal components. Individuals are colored by Baveno stage with 95% confidence ellipses, while arrows represent variable loadings. Panels 5-6: Bar plots depicting the relative contributions of individual variables to the first (PC1) and second (PC2) principal components. Abbreviations: (MFI) mean fluorescence intensity, (MELD) Model for End-Stage Liver Disease, (HE) hepatic encephalopathy, (Epi/LamProp) epithelium-to-lamina propria ratio, (CLS) confocal leak score, (HVPG) hepatic venous pressure gradient, (CRP) C-reactive protein.

Fig. S11. Incidence of liver-related events during the follow-up period: Stratification by presence of intraluminal fluorescein leakage (Vienna cohort).

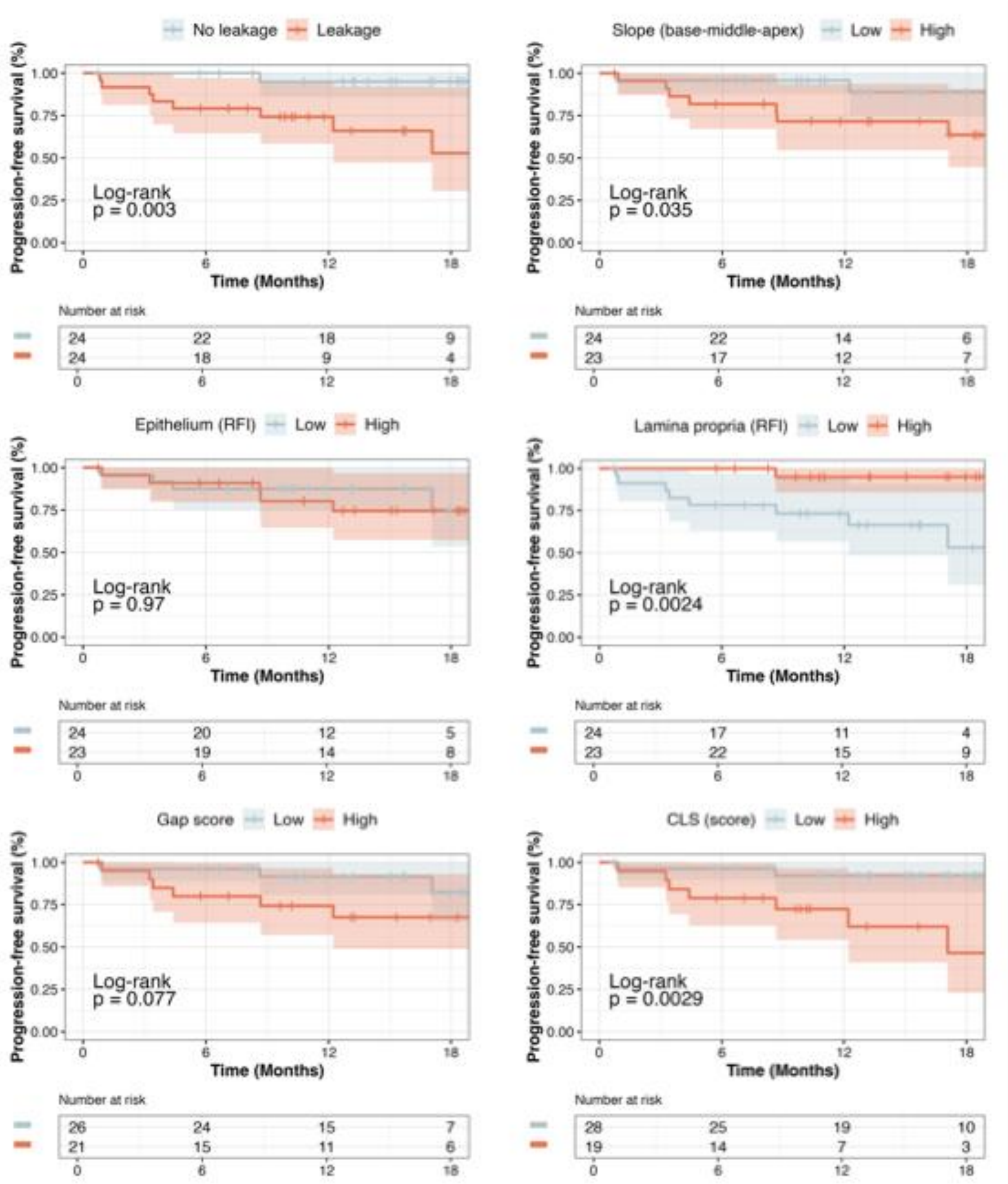

Statistical Analysis: Kaplan-Meier curves indicating disease progression-free follow-up (composite endpoint: first/further decompensation, ACLF, and liver related death) were compared by log-rank test. Panel 1: Groups are defined by presence or absence of intraluminal fluorescein leakage (binary classification). Panel 2-6: Groups are stratified by median CLE parameters (n=1 missing value due to technical issues with white balance of images). Abbreviations: (ACLF) Acute-on-Chronic Liver Failure, (CLE) Confocal Laser Endomicroscopy, (CLS) Confocal Leak Score, (RFI) Relative Fluorescence Intensity.

Fig. S12. Representative images for (A-C) Confocal leak score (CLS) and (D) Gap score.

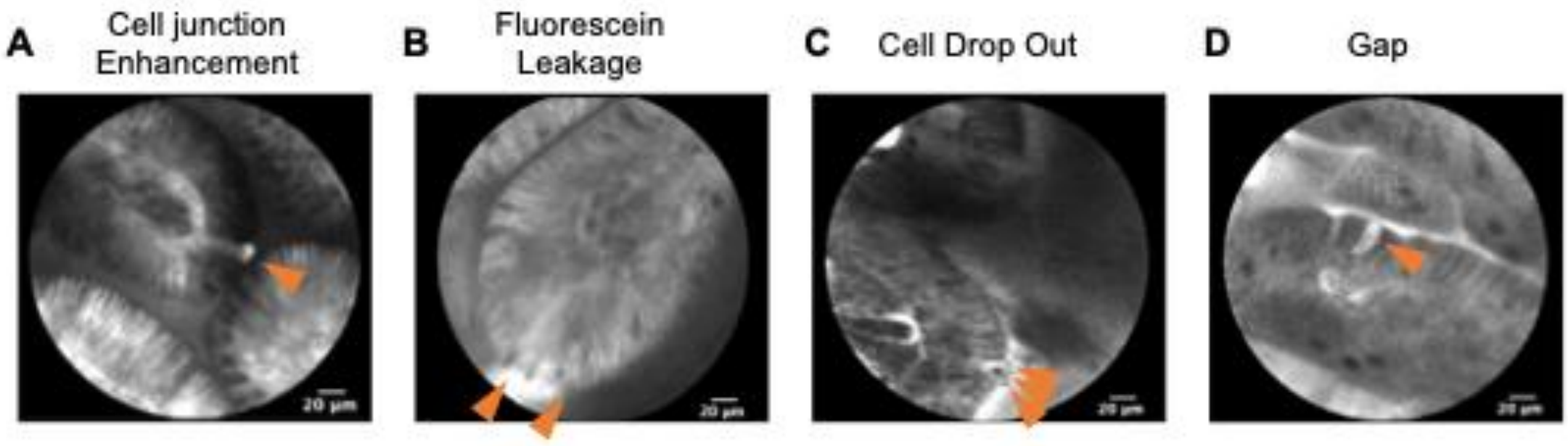

Supplementary tables

Table S1. Patient characteristics in patients stratified by CTP stage (Bern Cohort).

	Healthy<br>*N=4*	CTP-A<br>*N=4*	CTP-B<br>*N=6*	CTP-C<br>*N=6*	p-value
Age (Years)	44.3 (12.7)	61.2 (8.96)	55.5 (12.3)	53.2 (11.5)	0.250
Sex (n, %):					0.211
Female	0 (0.00%)	2 (50.0%)	1 (16.7%)	0 (0.00%)	
Male	4 (100%)	2 (50.0%)	5 (83.3%)	6 (100%)	
Etiology (n, %):					0.013
ALD	---	1 (25.0%)	3 (50.0%)	3 (50.0%)	
Viral	---	1 (25.0%)	0 (0.00%)	0 (0.00%)	
ALD&Viral	---	1 (25.0%)	1 (16.7%)	2 (33.3%)	
MASLD	---	1 (25.0%)	2 (33.3%)	0 (0.00%)	
Other	---	0 (0.00%)	0 (0.00%)	1 (16.7%)	
Ascites (n, %):					<0.001
Absent	---	4 (100%)	1 (16.7%)	0 (0.00%)	
Present	---	0 (0.00%)	5 (83.3%)	6 (100%)	
HE (n, %):					0.194
Absent	---	3 (75.0%)	3 (50.0%)	2 (33.3%)	
Present	---	1 (25.0%)	3 (50.0%)	4 (66.7%)	
CTP score	---	5.25 (0.50)	8.33 (0.82)	11.5 (1.22)	<0.001
MELD Score	---	9.00 [8.75;9.25]	11.0 [10.0;12.8]	24.0 [21.8;27.0]	0.018
Platelets (G/L)	255 (19.9)	142 (76.2)	98.0 (53.0)	68.2 (49.9)	<0.001
Bilirubin (µmol/L)	6.75 [4.80;8.60]	15.5 [11.8;20.2]	13.5 [12.0;21.0]	60.0 [33.5;96.2]	0.005
Albumin (g/L)	35.5 (4.80)	37.8 (3.30)	28.3 (3.83)	26.8 (2.71)	0.001
CRP (mg/L)	0.15 [0.08;0.28]	8.50 [7.62;9.38]	13.5 [10.5;23.2]	4.00 [3.25;10.8]	0.007

Statistical Analysis: Kruskal Wallis test or ANOVA were used to compare continuous variables. Group comparisons of categorical variables were performed using Chi squared or Fisher's Exact test. Abbreviations: (CTP) Child-Turcotte-Pugh; (ALD) Alcohol-Related Liver Disease, (CHOL) Cholestatic Liver Disease, (MASLD) Metabolic Dysfunction-Associated Liver Disease, (MELD) Model for End-Stage Liver Disease, (HE) Hepatic Encephalopathy, (CRP) C-Reactive Protein

Table S2. Patient characteristics across CTP stages (Vienna Cohort).

	A *N=25*	B *N=14*	C *N=9*	p-value
MELD Score	10.0 [8.00;11.0]	13.0 [12.0;15.0]	25.0 [20.0;26.0]	<0.001
HVPG (mmHg)	16.4 (4.77)	18.2 (5.69)	19.0 (3.89)	0.358
Etiology (n, %):				0.290
ALD	13 (52.0%)	6 (42.9%)	7 (77.8%)	
Cholestatic	3 (12.0%)	3 (21.4%)	0 (0.00%)	
MASLD	2 (8.00%)	1 (7.14%)	0 (0.00%)	
ALD&Viral	3 (12.0%)	0 (0.00%)	2 (22.2%)	
Other	2 (8.00%)	4 (28.6%)	0 (0.00%)	
Viral	2 (8.00%)	0 (0.00%)	0 (0.00%)	
Sex (n, %):				0.917
F	7 (28.0%)	5 (35.7%)	3 (33.3%)	
M	18 (72.0%)	9 (64.3%)	6 (66.7%)	
Age (Years)	58.6 (11.6)	50.7 (17.7)	53.8 (10.9)	0.215
Varices (n, %):				0.645
No	7 (28.0%)	3 (21.4%)	1 (11.1%)	
Small	9 (36.0%)	3 (21.4%)	4 (44.4%)	
Large	9 (36.0%)	8 (57.1%)	4 (44.4%)	
Ascites (n, %):				<0.001
No	21 (84.0%)	2 (14.3%)	0 (0.00%)	
Mild	4 (16.0%)	10 (71.4%)	6 (66.7%)	
Severe	0 (0.00%)	2 (14.3%)	3 (33.3%)	
Hepatic Encephalopathy (n, %):				<0.001
No	25 (100%)	12 (85.7%)	3 (33.3%)	
Mild	0 (0.00%)	2 (14.3%)	5 (55.6%)	
Severe	0 (0.00%)	0 (0.00%)	1 (11.1%)	
Albumin (g/L)	40.0 (3.73)	34.0 (3.23)	28.5 (4.22)	<0.001
CRP (mg/dL)	0.27 [0.10;0.54]	0.44 [0.32;0.61]	0.71 [0.58;0.76]	0.044
Gap score	0.00 [0.00;0.22]	0.21 [0.00;0.32]	0.00 [0.00;0.39]	0.465
CLS (score)	40.0 [20.0;52.5]	60.0 [26.7;84.3]	80.0 [50.0;90.0]	0.055
Apex/Base ratio	0.69 [0.53;0.80]	0.83 [0.71;0.94]	1.04 [0.79;1.11]	0.008
Middle/Base ratio	0.79 [0.77;0.88]	0.84 [0.77;0.94]	0.91 [0.77;0.94]	0.597
Apex/Middle ratio	0.83 (0.19)	1.01 (0.30)	1.17 (0.22)	0.002
Epi/LamProp ratio	0.67 [0.57;0.79]	0.68 [0.58;0.77]	0.70 [0.63;0.90]	0.703
Epi/Lumen ratio	2.11 [1.23;3.53]	1.43 [0.66;3.53]	0.73 [0.61;1.05]	0.057
LamProp/Lumen ratio	2.68 [1.58;6.52]	1.71 [1.22;5.82]	1.01 [0.86;1.26]	0.021
Slope (base-middle-apex)	-14.97 (12.3)	-6.03 (15.0)	1.39 (12.3)	0.007
Epithelium (RFI)	0.33 (0.07)	0.30 (0.06)	0.29 (0.05)	0.231
Lamina propria (RFI)	0.49 (0.09)	0.45 (0.11)	0.39 (0.11)	0.058
Lumen (RFI)	0.17 [0.09;0.25]	0.23 [0.09;0.34]	0.36 [0.33;0.42]	0.027
Fluorescein leakage (n, %):				0.011
Absent	17 (68.0%)	6 (42.9%)	1 (11.1%)	
Present	8 (32.0%)	8 (57.1%)	8 (88.9%)	

Statistical Analysis: Kruskal Wallis test or ANOVA were used to compare continuous variables. Group comparisons of categorical variables were performed using Chi squared or Fisher's Exact test. Abbreviations: (CTP) Child-Turcotte-Pugh, (cACLD) compensated advanced chronic liver disease, (MELD) Model for End-Stage Liver Disease, (HVPG) Hepatic Venous Pressure Gradient, (ALD) Alcohol-Related Liver Disease, (MASLD) Metabolic Dysfunction-Associated Liver Disease, (HE) Hepatic Encephalopathy, (CRP) C-Reactive Protein, (CLS) Confocal Leak Score, (RFI) Relative Fluorescence Intensity.

Table S3. Availability of blood biomarkers (Vienna Cohort).

Parameter	N available (total: 48)
WBC (G/L)	48
Bilirubin (mg/dL)	48
Albumin (g/L)	48
CRP (mg/dL)	48
VWF-Ag (%)	38
PCT (ng/mL)	23
ELF score	24
BA (µmol/L)	25
IgG (g/L)	26
IgA (g/L)	26
IgM (g/L)	26
IgG1 (g/L)	22
IgG2 (g/L)	22
IgG3 (g/L)	22
IgG4 (g/L)	22

Abbreviations: (WBC) White Blood Cells, (CRP) C-Reactive Protein, (VWF-Ag) von Willebrand factor antigen, (PCT) Procalcitonin, (ELF) Enhanced Liver Fibrosis score, (BA) Bile acids, (Ig) Immunoglobulin.

Supplementary references

1. Chang, J., et al., *Impaired Intestinal Permeability Contributes to Ongoing Bowel Symptoms in Patients With Inflammatory Bowel Disease and Mucosal Healing.* Gastroenterology, 2017. **153**(3): p. 723-731.e1.
2. Shavrov, A., et al., *A Pilot Study of Confocal Laser Endomicroscopy to Predict Barrier Dysfunction and Relapse in Pediatric Inflammatory Bowel Disease.* J Pediatr Gastroenterol Nutr, 2016. **62**(6): p. 873-8.
3. de Franchis, R., et al., *Baveno VII - Renewing consensus in portal hypertension.* J Hepatol, 2022. **76**(4): p. 959-974.
