# Supplementary material for: Intestinal barrier dysfunction assessed by confocal laser endomicroscopy correlates with cirrhosis severity and clinical outcomes
Source: JHEP Rep. 2026 Sep 18;8(12):102037. doi: 10.1016/j.jhepr.2026.102037 (PMC13756262; doi:10.1016/j.jhepr.2026.102037)
Supplement: Supplementary file 3 [file mmc4.pdf]

Intestinal barrier dysfunction assessed by confocal laser endomicroscopy correlates with cirrhosis severity and clinical outcomes

Authors

Benedikt Simbrunner, Marcel Sorribas, Jessica Schaerer, …, Thomas Reiberger, Johann Hammer, Reiner Wiest

Correspondence

thomas.reiberger@meduniwien.ac.at (T. Reiberger).

Graphical abstract

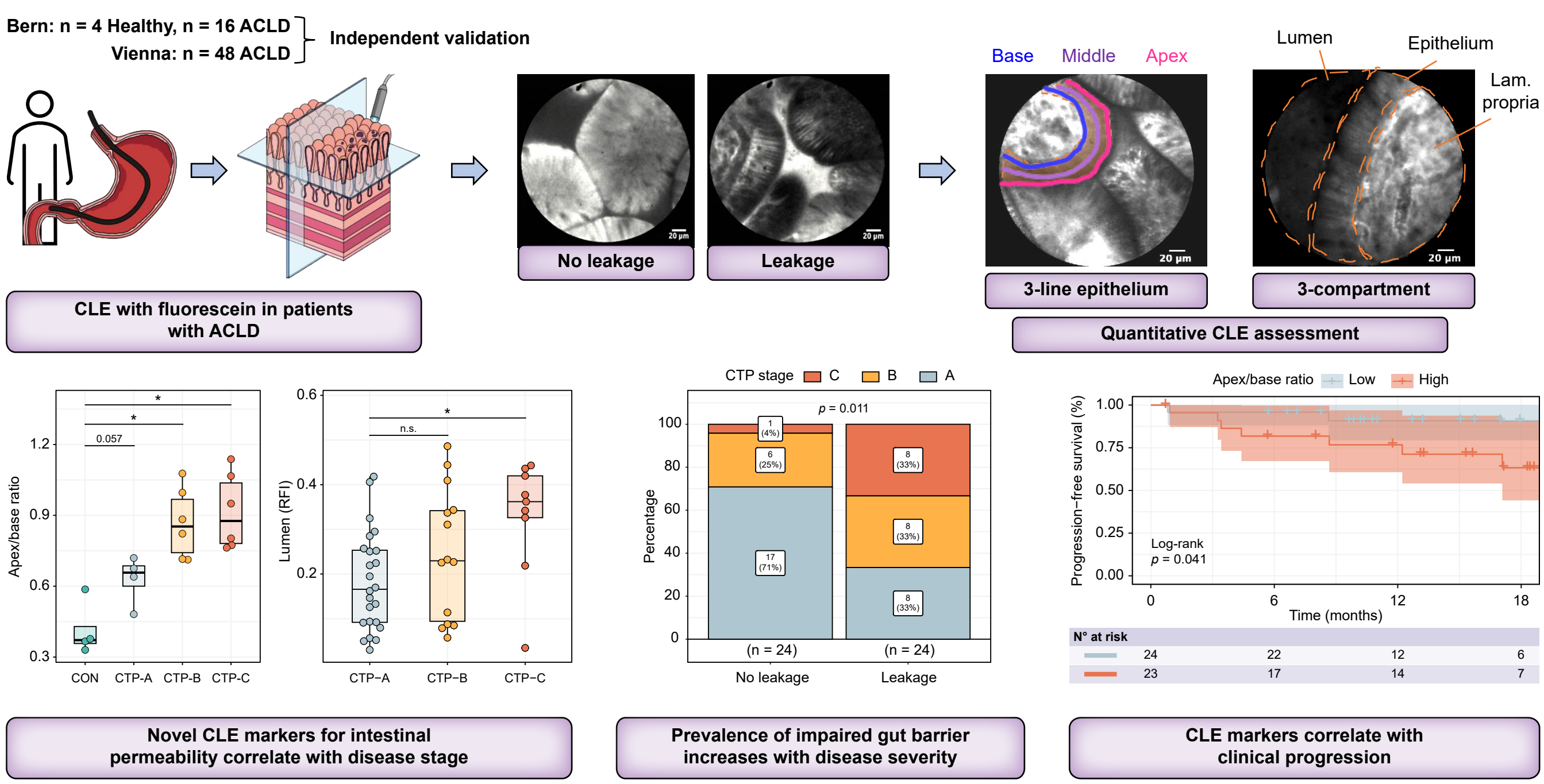

Highlights:

- CLE-based visualization of gut barrier permeability in patients with cirrhosis performed in two European transplant centers.
- Novel quantitative 3-line/3-compartment CLE analysis methods are reproducible.
- Transepithelial fluorescein leakage is elevated compared with healthy controls and further rises with cirrhosis severity.
- CLE permeability metrics correlate with portal hypertension and liver function.
- Higher CLE-assessed intestinal permeability is linked to liver-related events.

Impact and implications:

Intestinal barrier dysfunction is a key driver of complications in advanced chronic liver disease. Using CLE during endoscopy, the present study shows that transepithelial fluorescein leakage can be visualized and quantified, and aligns with cirrhosis stage, portal hypertension, and liver-related outcomes. These findings are relevant for hepatologists and translational researchers, as this technique might be used to identify patients with intestinal barrier dysfunction. Because the present analyses are exploratory and derive from two cohorts with limited sample size and requirement for manual image analysis, larger prospective studies and feasibility of (semi-)automated quantification are required before CLE-based permeability assessment can be applied more broadly.

https://doi.org/10.1016/j.jhepr.2026.102037
© 2026 The Authors. Published by Elsevier B.V. on behalf of European Association for the Study of the Liver (EASL). This is an open access article under the CC BY license (http://creativecommons.org/licenses/by/4.0/).

Intestinal barrier dysfunction assessed by confocal laser endomicroscopy correlates with cirrhosis severity and clinical outcomes☆

Benedikt Simbrunner[1,2,3,4,5,†], **Marcel Sorribas**[6,7,†], Jessica Schaerer[8], Marco Felber[9], Jordi Codinach[10], Susana Gomes Rodrigues[8], Mattias Mandorfer[1,2,3], Annalisa Berzigotti[8], Michael Trauner[1,3], Thomas Reiberger[1,2,3,4,5,*], Johann Hammer[1,‡], Reiner Wiest[8,‡]

JHEP Reports **2026**. vol. 8 | 1–13

Check for updates

Background & Aims: Intestinal barrier dysfunction drives complications in advanced chronic liver disease (ACLD). Confocal laser endomicroscopy (CLE) enables direct visualization of transepithelial fluorescein leakage during endoscopy. This study evaluated the link between CLE-assessed intestinal permeability and disease severity in ACLD.

Methods: CLE was performed in two independent cohorts after intravenous fluorescein administration in the small intestine: (1) Bern cohort (n = 4 healthy controls, n = 16 ACLD) and (2) Vienna cohort (n = 48 ACLD). Image analysis included established CLE scores, and novel quantitative assessments measuring fluorescence intensity in three compartments ('3-compartment-method'; lumen–epithelium–lamina propria) and along the epithelium ('3-line-method'; base–middle–apex). CLE-derived metrics were correlated with disease stage and biomarkers. In the Vienna cohort, correlation with portal hypertension (HVPG) and liver-related events (decompensation, ACLF, death) was assessed.

Results: Transepithelial fluorescein permeation into the gut lumen was absent in healthy controls but frequent in ACLD, and increased with disease severity (Vienna cohort: compensated *vs.* first decompensation *vs.* further decompensation: 24%, 57%, and 71%, $p = 0.019$). The 3-line and 3-compartment methods robustly distinguished healthy controls from cirrhosis (3-line apex/base ratio: 0.42 ± 0.12 *vs.* ACLD 0.83 ± 0.18, $p = 0.001$), and exhibited lowest interobserver variability. In both cohorts, quantitative permeability measures – especially epithelial 3-line apex/base ratio and 3-line-slopes (all $p < 0.05$) – increased across Child-Turcotte-Pugh stages, and correlated with HVPG (apex/base ratio: $r = 0.575$, $p < 0.001$) and liver function parameters (*e.g.* albumin: $r = -0.553$, $p < 0.001$). CLE-based barrier dysfunction was linked to higher liver-related event incidence during follow-up (Cox regression; apex/base ratio: hazard ratio 10.6, 95% CI 1.71–66.0, $p = 0.011$).

Conclusions: CLE identifies high prevalence of intestinal permeability in ACLD, correlating with disease stage and adverse outcomes. The novel 3-line epithelial analysis demonstrated strong associations with portal hypertension and biomarkers reflecting ACLD severity.

Clinical Trials Registration: The study is registered at ClinicalTrials.gov (NCT03267615).

© 2026 The Authors. Published by Elsevier B.V. on behalf of European Association for the Study of the Liver (EASL). This is an open access article under the CC BY license (http://creativecommons.org/licenses/by/4.0/).

Introduction

Advanced chronic liver disease (ACLD) is characterized by advanced fibrosis, functional and structural distortion of hepatic tissue, culminating in portal hypertension and liver dysfunction. Beyond intrinsic hepatic damage, the gut–liver axis plays a central role in the pathogenesis and progression of cirrhosis.[1,2] Intestinal barrier dysfunction in patients with ACLD results in increased intestinal permeability, facilitates bacterial translocation and the systemic dissemination of pathogen-associated molecular patterns (PAMPs). This perpetuates inflammation and immune activation, thus exacerbating liver injury and contributing to progression of portal hypertension and fibrosis.[3–5]

These processes establish a vicious cycle of gut and liver dysfunction; wherein gut barrier failure amplifies inflammation and hepatic decompensation. Despite its critical role in disease progression, the clinical assessment of intestinal permeability remains challenging.[6–8] Conventional methods, including serum biomarkers and indirect assays, often lack specificity and spatial resolution. Furthermore, mucosa biopsies, although informative,

☆ Given their role as Associate Editor, Mattias Mandorfer, had no involvement in the peer-review of this article and had no access to information regarding its peer-review. Full responsibility for the editorial process for this article was delegated to the Co-Editor Viginia Hernández-Gea.

* Corresponding author. Address: Division of Gastroenterology and Hepatology, Department of Medicine III, Medical University of Vienna, Waehringer Guertel 18-20, 1090 Vienna, Austria. Tel.: +43 1 40400 47440; fax: +43 1 40400 47350.
E-mail address: thomas.reiberger@meduniwien.ac.at (T. Reiberger).
† Authors contributed equally and share first authorship.
‡ Authors share last/senior authorship.

https://doi.org/10.1016/j.jhepr.2026.102037

are impractical for routine clinical assessment of barrier function because of delayed results.[9] These limitations underscore the need for direct, *in vivo* visualization of intestinal barrier integrity.

Confocal laser endomicroscopy (CLE) is an advanced imaging technique that enables *in vivo*, high-resolution assessment of the intestinal mucosa at the cellular and subcellular levels during endoscopy.[10–13] By integrating a confocal microscope into an endoscope and using fluorescein contrast, CLE provides insight into mucosal integrity by assessing fluorescein leakage, a marker of barrier dysfunction.

The primary aim of this study was to evaluate whether CLE-based assessment of intestinal barrier function detects increased transepithelial permeability that relates to disease severity in patients with ACLD. To this end, we developed and evaluated two novel quantitative CLE analysis techniques (3-line and 3-compartment methods) alongside established qualitative scores, and used a small group of healthy controls to test whether these readouts already deviate in early (compensated) ACLD. As exploratory objectives, we examined the relationship between CLE-derived permeability metrics and established markers of disease severity and liver-related events to explore their potential clinical relevance.

Patients and methods

Study design and clinical characterization

Patients with ACLD undergoing routine gastrointestinal endoscopy with CLE were prospectively recruited at two European tertiary centers: University Hospital Bern (Bern, Switzerland) and the Medical University of Vienna (Vienna, Austria).

In the Bern cohort, patients with ACLD undergoing screening or surveillance for esophageal varices were eligible. Exclusion criteria were recent gastrointestinal bleeding (<3 months) and active malignancy. Furthermore, healthy controls were recruited among individuals undergoing endoscopy for functional gastrointestinal complaints, provided that they had no relevant comorbidities, were not receiving ongoing medication, and showed no gastrointestinal pathology during endoscopy. General exclusion criteria were a known allergy to fluorescein, pregnancy or breast feeding, or impaired renal function (estimated glomerular filtration rate <45 ml/min).

In the Vienna cohort, patients with ACLD were included when at least one of the following criteria were present: HVPG ≥6 mmHg, liver stiffness measurement ≥10 kPa, histological fibrosis stage F3/F4, or radiological evidence of cirrhosis. Patients with posthepatic or presinusoidal portal hypertension or previous liver transplantation were not considered for the study. Hepatic decompensation events previous to/at the timepoint of endoscopy were determined according to Austrian[14] and European guidelines[15] and Baveno VII consensus criteria.[16] Laboratory parameters were obtained from electronic hospital records and were analyzed by the Department of Laboratory Medicine at the Medical University of Vienna, following International Organization for Standardization (ISO)-certified measurement protocols.

Endoscopic procedures and confocal laser endomicroscopy

Informed consent was obtained before endoscopy, which was performed under conscious sedation utilizing standard endoscopes (GIF-HQ190 endoscope and CV-190 imaging system, Olympus Corporation, Tokyo, Japan). CLE was performed during the endoscopy procedure using a Cellvizio probe (Mauna Kea Technologies, Paris, France) to obtain high-resolution, *in vivo* imaging of the second part of the duodenum. A single intravenous bolus of 2.5-5 ml fluorescein (SERB Pharmaceuticals, Paris, France) was used as a systemic contrast agent to enhance visualization and assess intestinal permeability. The small intestinal mucosa was inspected for up to 8 min post-fluorescein injection, focusing on identifying luminal transposition of fluorescein. Analysis was performed on selected images acquired within 2 min after first appearance of fluorescein in the microcirculation seen on CLE after injection, limiting the additional procedural time required for CLE. The presence of fluorescein leakage into the gut lumen was assessed, in line with previously published Watson scoring,[13] which distinguishes between absence of leakage into the gut lumen (referred to as 'No leakage') and the presence of leakage with or without cell shedding ('Leakage').

CLE image analysis

For the assessment of CLE-based qualitative and quantitative readouts of transepithelial fluorescein permeation, 10 representative images or more were selected and analyzed using Fiji (ImageJ).

Image analysis

Cellvizio Viewer software (Cellvizio Viewer Version 1.4.2; Mauna Kea Technologies, Paris, France) was used to extract the photograms from the CLE video. An adequate image was defined as one that fulfilled each of the following criteria: presence of vasculature/vessel, epithelium and lumen, and high contrast/delineation of morphology (Fig. 1). The analysis of the confocal images was conducted in a blinded manner by two independent observers at each center. Videos were reviewed and images of sufficient quality taken after first appearance of fluorescence were analyzed. Observers at each center analyzed the same pre-selected images, blinded to the measurements of the other observer.

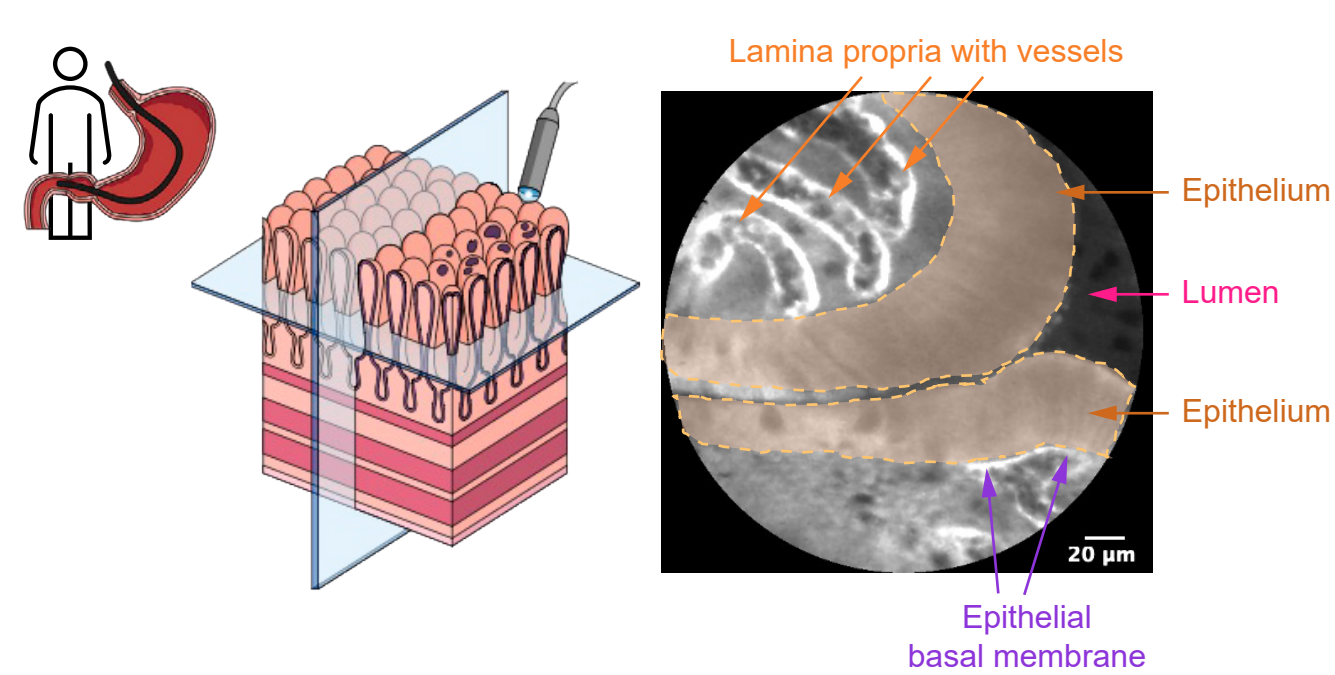

Fig. 1. Confocal laser endomicroscopy (CLE) of the small intestinal mucosa in patients with advanced chronic liver disease. Representative confocal laser endomicroscopy images of the small intestinal mucosa after intravenous fluorescein administration, demonstrating epithelial architecture, lamina propria with vessels, and luminal compartment.

Three-compartment-analysis

The lamina propria, epithelium, and lumen compartments of a villus were manually outlined using the polygon tool in ImageJ. For each compartment the area and the mean fluorescence intensity (MFI) were computed (Fig. 2A). The sum of MFIs from all three compartments was defined as 100%, and the proportion of fluorescence intensity within each compartment was expressed as a percentage of this total, thereby defining the relative fluorescence intensity (RFI).

Three-line method assessing gut epithelial barrier

Fluorescence intensity (FI, 'gray value') within the epithelial layer was measured at three predefined levels along the epithelial lining using Image J (Fig. 2B): base, middle, and apex. For each line, pixel-wise gray values were averaged to obtain the MFI. Ratios between the three epithelial levels and slopes were calculated based on the assumption that the amount of fluorescein determines fluorescence intensity as it permeates from the basal to the apical epithelial layers. Further details are delineated in the Supplementary material.

Confocal leak score

The Confocal Leak score (CLS) was assessed as previously described by Chang and colleagues[17] in the context of inflammatory bowel disease (IBD). A detailed method can be found in the Supplementary material.

Gap score

Epithelial gap density was determined as previously described in the context of irritable bowel syndrome.[18] A detailed methodological description is delineated in the Supplementary material.

Liver-related events during the follow-up period

Liver-related events denoting disease progression were recorded during follow-up in the Vienna cohort, that is, the incidence of first decompensation, further decompensation, acute-on-chronic liver failure, and liver-related death (LRD). Patients were followed at routine clinical visits (every 6 months in compensated ACLD [cACLD]; every 3 months in decompensated ACLD, or shorter as clinically indicated), with decompensation events systematically recorded at each visit, as previously described.[19] Patients were censored at the date of last clinical contact or liver transplantation in case no event occurred. In patients with cACLD, first decompensation was defined as the incidence of clinically evident ascites, variceal bleeding, or overt hepatic encephalopathy (HE). In patients with decompensated ACLD (dACLD), further decompensation was defined as the incidence of a second decompensating event or recurrent/refractory ascites, overt HE, variceal bleeding, and/or incidence of spontaneous bacterial peritonitis (SBP), hepatorenal syndrome–acute kidney injury (HRS-AKI), in line with Baveno VII consensus criteria.[16] Furthermore, the incidence of acute-on-chronic liver failure according to guidelines by the EASL was recorded.[20]

Statistical analysis

Statistical analyses were performed using R 4.3.2 (R Foundation for Statistical Computing, Vienna, Austria). Continuous variables were compared using Student's *t* test or Mann-Whitney *U* test for comparisons of two groups, and one-way ANOVA or Kruskal-Wallis tests with Benjamini-Hochberg-adjusted pairwise Wilcoxon rank-sum tests, as appropriate, for comparisons of three groups. Kaplan-Meier curves and log-rank tests were used to visualize and compare the incidence of liver-related events between groups ('survival' package). Univariable Cox regression analyses were performed to evaluate whether individual parameters were associated with liver-related events (hazard ratios with 95% CIs). Interobserver agreement was assessed using the intraclass correlation coefficient (ICC) based on a two-way random-effects model with absolute agreement. Ninety-five percent confidence intervals were calculated for ICC estimates. Principal component analysis (PCA) was performed on centered and scaled variables using the 'FactoMineR' package, with visualization of biplots, scree plots, variable contributions, and stage-stratified individual factor maps generated using 'factoextra'. Additional information on statistical analyses is delineated in the Supplementary material.

Ethical considerations

The study was conducted in accordance with the principles of the Declaration of Helsinki and its amendments and approved by the local ethics committees (Vienna: EK 1262/2017; 1239/2023; Bern: KEK-2020-00873). Patients and healthy individuals provided written informed consent for endoscopic procedures.

Results

Patient characteristics

The Bern cohort comprised four healthy individuals and 16 patients with ACLD. Clinical and laboratory characteristics are summarized in Table S1. Briefly, patients had a median age of 53 (IQR: 50–63) years, were predominantly male (n = 13/16, 81%), and alcohol-related liver disease (ALD; n = 7, 44%) was the most prevalent etiology of ACLD. The distribution of patients across Child-Turcotte-Pugh (CTP) stages comprised n = 4 (25%) CTP-A, n = 6 (37.5%) CTP-B, and n = 6 (37.5%) CTP-C.

The Vienna cohort comprised 48 patients with ACLD with a median age of 55 years (IQR: 43–69) and 69% were male. Patient characteristics are summarized in Table 1. Median hepatic venous pressure gradient (HVPG) was 17 mmHg (IQR: 14–21; available in n = 43; median time interval to endoscopy: 2.20 [0.00–18.0] months), median model for end-stage liver disease (MELD) score was 12 (IQR: 9–15). ALD was the most common etiology of liver disease, accounting for 54% of the cohort. According to the CTP classification, 52% of patients were CTP-A (n = 25), 29% CTP-B (n = 14), and 19% CTP-C (n = 9). MELD and HVPG gradually increased across clinical stages (Table 1).

Exploration of novel CLE-based intestinal barrier analysis

To assess transepithelial fluorescein permeation of the intestinal mucosa, the 3-line method was applied in healthy controls

A

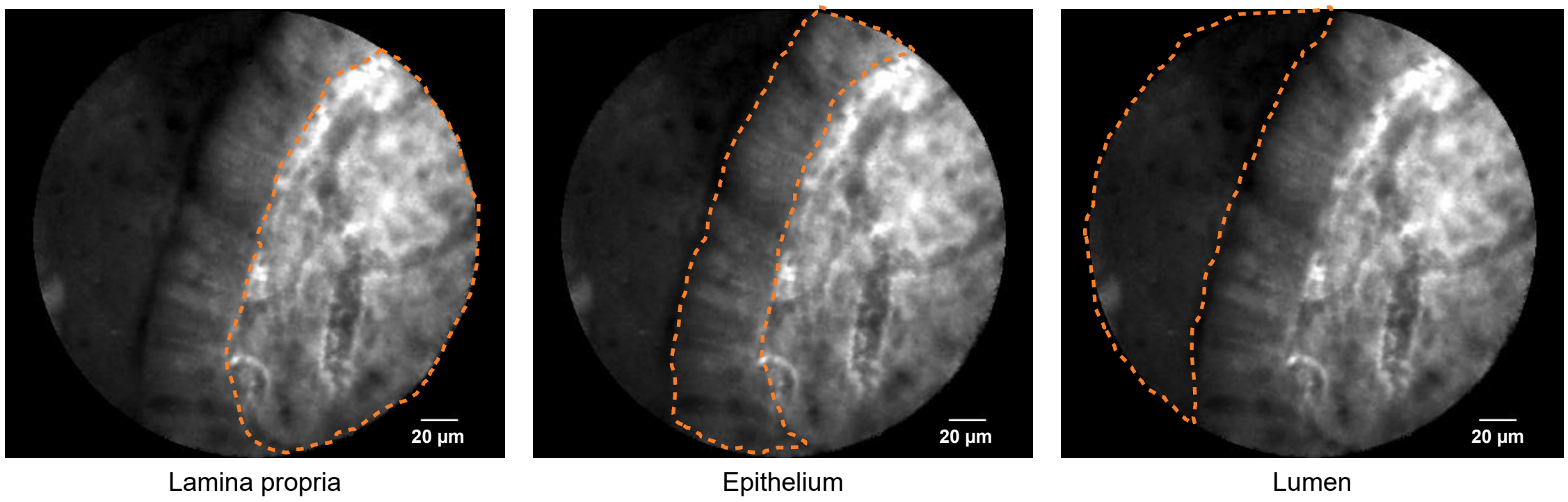

	Area	Mean	StdDev	Min	Max	Perim.
1	31,520	141.409	45.035	40	255	679.573
2	29,939	60.843	32.304	10	163	1,274.522
3	23,617	33.379	14.421	0	73	1,359.313

B

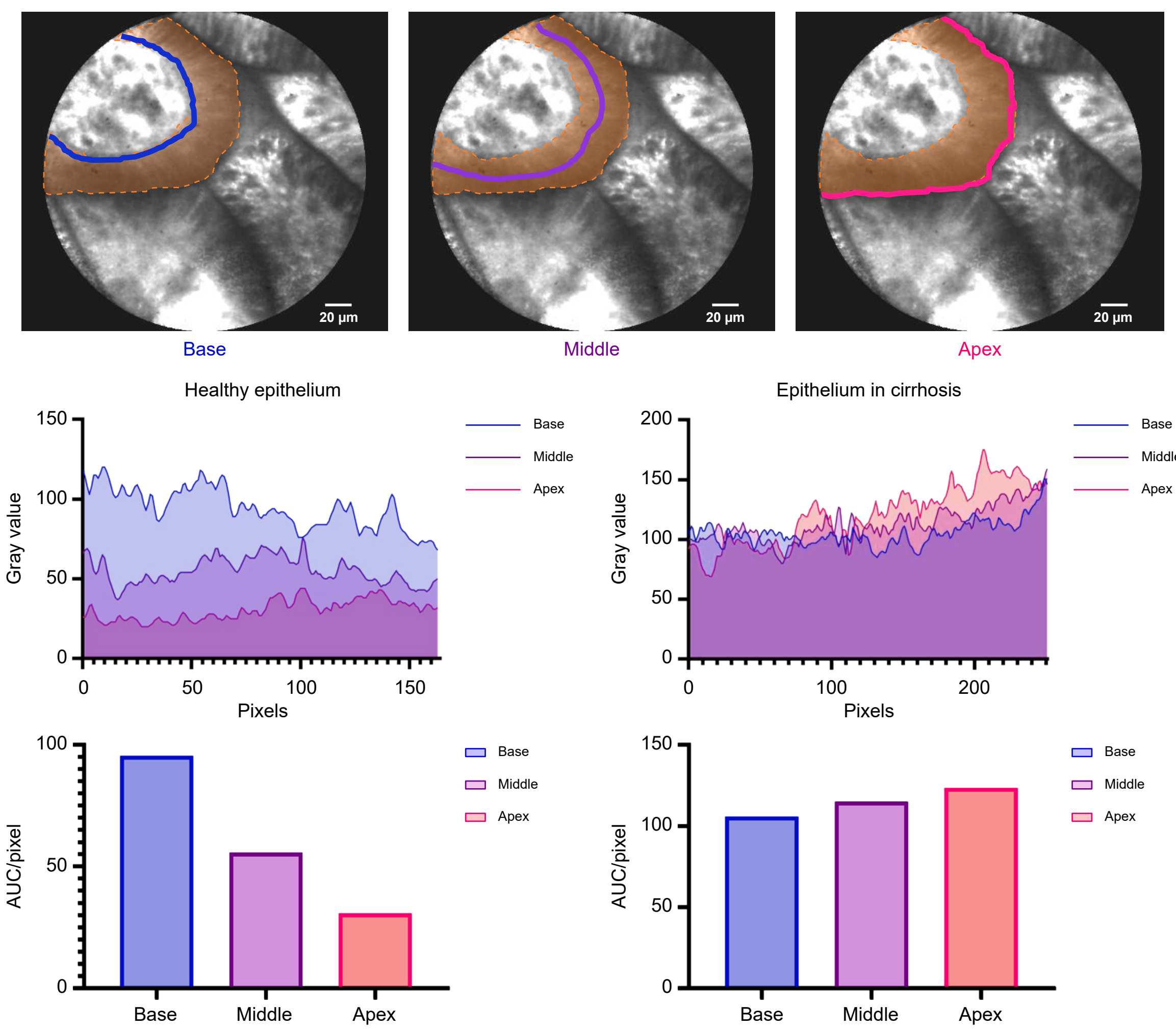

Fig. 2. Novel quantitative CLE image analysis: 3-compartment and 3-line methods. Schematic overview of quantitative CLE image analysis. (A) Three-compartment analysis of epithelium, lamina propria, and lumen. (B) Three-line analysis assessing fluorescence intensity along the base–middle–apex axis of the intestinal epithelium.

Table 1. Characteristics of the Vienna cohort stratified by clinical disease stages: cACLD, first decompensation, and further decompensation.

	Overall cohort	cACLD	FirstDec	FurtherDec	
	N = 48	n = 17	n = 14	n = 17	*p* value
CTP score	6.00 [5.00; 8.00]	5.00 [5.00; 5.00]	6.50 [5.25; 7.00]	8.00 [7.00; 12.0]	<0.001
CTP stage, n (%):					<0.001
A	25 (52.1)	16 (94.1)	7 (50.0)	2 (11.8)	
B	14 (29.2)	1 (5.88)	6 (42.9)	7 (41.2)	
C	9 (18.8)	0 (0.00)	1 (7.14)	8 (47.1)	
MELD score	12.0 [9.00; 15.2]	10.0 [8.00; 12.0]	11.0 [10.0; 13.5]	20.0 [12.0; 21.0]	0.001
HVPG (mmHg)	17.4 (4.94)	14.6 (4.57)	18.6 (5.80)	19.0 (3.26)	0.030
Etiology, n (%):					0.471
ALD	26 (54.2)	7 (41.2)	7 (50.0)	12 (70.6)	
Cholestatic	6 (12.5)	3 (17.6)	3 (21.4)	0 (0.00)	
MASLD	3 (6.25)	1 (5.88)	1 (7.14)	1 (5.88)	
ALD and viral	5 (10.4)	2 (11.8)	2 (14.3)	1 (5.88)	
Other	6 (12.5)	2 (11.8)	1 (7.14)	3 (17.6)	
Viral	2 (4.17)	2 (11.8)	0 (0.00)	0 (0.00)	
Sex (n, %):					0.075
F	15 (31.2)	2 (11.8)	7 (50.0)	6 (35.3)	
M	33 (68.8)	15 (88.2)	7 (50.0)	11 (64.7)	
Age (years)	55.4 (13.7)	59.7 (11.7)	52.3 (17.0)	53.5 (12.3)	0.263
Varices (n, %):					0.054
No	11 (22.9)	6 (35.3)	3 (21.4)	2 (11.8)	
Small	16 (33.3)	8 (47.1)	2 (14.3)	6 (35.3)	
Large	21 (43.8)	3 (17.6)	9 (64.3)	9 (52.9)	
Ascites (n, %):					<0.001
No	23 (47.9)	17 (100)	5 (35.7)	1 (5.88)	
Mild	20 (41.7)	0 (0.00)	8 (57.1)	12 (70.6)	
Severe/recurrent	5 (10.4)	0 (0.00)	1 (7.14)	4 (23.5)	
HE (n, %):					<0.001
No	40 (83.3)	17 (100)	14 (100)	9 (52.9)	
Mild	7 (14.6)	0 (0.00)	0 (0.00)	7 (41.2)	
Severe/recurrent	1 (2.08)	0 (0.00)	0 (0.00)	1 (5.88)	
Fluorescein leakage (n, %)					0.019
Absent	24 (50.0)	13 (76.5)	6 (42.9)	5 (29.4)	
Present	24 (50.0)	4 (23.5)	8 (57.1)	12 (70.6)	

Statistical Analysis: Kruskal-Wallis test or ANOVA were used to compare continuous variables. Group comparisons of categorical variables were performed using X^2 or Fisher's Exact test. ALD, alcohol-related liver disease; cACLD, compensated advanced chronic liver disease; CTP, Child-Turcotte-Pugh; HVPG, hepatic venous pressure gradient; MASLD, metabolic dysfunction-associated liver disease; MELD, model for end-stage liver disease.

and patients with cirrhosis from the Bern cohort (Fig. 3A and Fig. S1). In healthy controls, mean RFI was highest at the basal epithelial layer and progressively decreased towards the middle and apical layers. This gradient was also present in ACLD (Fig. S1A), but RFI values differed from controls: patients with ACLD exhibited significantly higher apical RFI compared with healthy controls (0.29 [0.28–0.30] *vs.* 0.18 [0.16–0.20], p = 0.003), whereas basal RFI was lower (0.39 [0.37–0.40] *vs.* 0.50 [0.48–0.51], p = 0.003). No significant group differences were observed at the middle epithelial layer. Consequently, the apex/base ratio significantly increased in patients with ACLD compared with healthy controls (Healthy: 0.42 ± 0.12, ACLD: 0.83 ± 0.18, p = 0.001), indicating enhanced transepithelial fluorescein passage towards the luminal surface in cirrhosis. Stratification by CTP stages revealed a stepwise increase of apex/base ratio with advancing disease severity: 0.42 ± 0.12 in healthy controls, 0.63 ± 0.10 in CTP-A, 0.87 ± 0.15 in CTP-B, and 0.92 ± 0.16 in CTP-C patients (overall p <0.001; Fig. 3A). Similar dynamics were present when stratifying patients by the presence of ascites (Fig. S1).

In the 3-compartment analysis (lamina propria, epithelium, and lumen), a physiological fluorescence gradient was observed across both healthy controls and patients with ACLD, characterized by decreasing RFI from the lamina propria to the epithelium and further into the intestinal lumen (Fig. S2). This pattern suggests preserved compartmental organization of fluorescein distribution. Lamina propria RFI was significantly reduced in patients with advanced cirrhosis compared with healthy controls (0.40 [0.39–0.45] *vs.* 0.57 [0.53–0.59], p = 0.018), most prominently in patients who had CTP-C (Fig. S2). In contrast, epithelial (0.34 ± 0.07 *vs.* 0.28 ± 0.04, p = 0.032) and luminal RFI (0.23 ± 0.11 *vs.* 0.17 ± 0.04; p = 0.096) were higher in patients with ACLD compared with healthy controls, consistent with enhanced transepithelial fluorescein leakage and redistribution towards the luminal compartment in advanced disease.

Together, these findings support progressive leakage of fluorescein across the epithelium into the lumen indicating an impairment of the intestinal barrier with increasing cirrhosis severity.

To compare the new quantitative CLE readouts against previously established scores, CLS and Gap score (Fig. S3) were assessed. In summary, both CLS and Gap score exhibited numerical trends toward increased mucosal barrier disruption, however, neither method was able to robustly discriminate between cirrhosis stages (see Supplementary material).

Furthermore, correlation analyses were performed to explore interactions between the novel CLE parameters and routine blood analyses used in the context of liver disease (Fig. 3B).

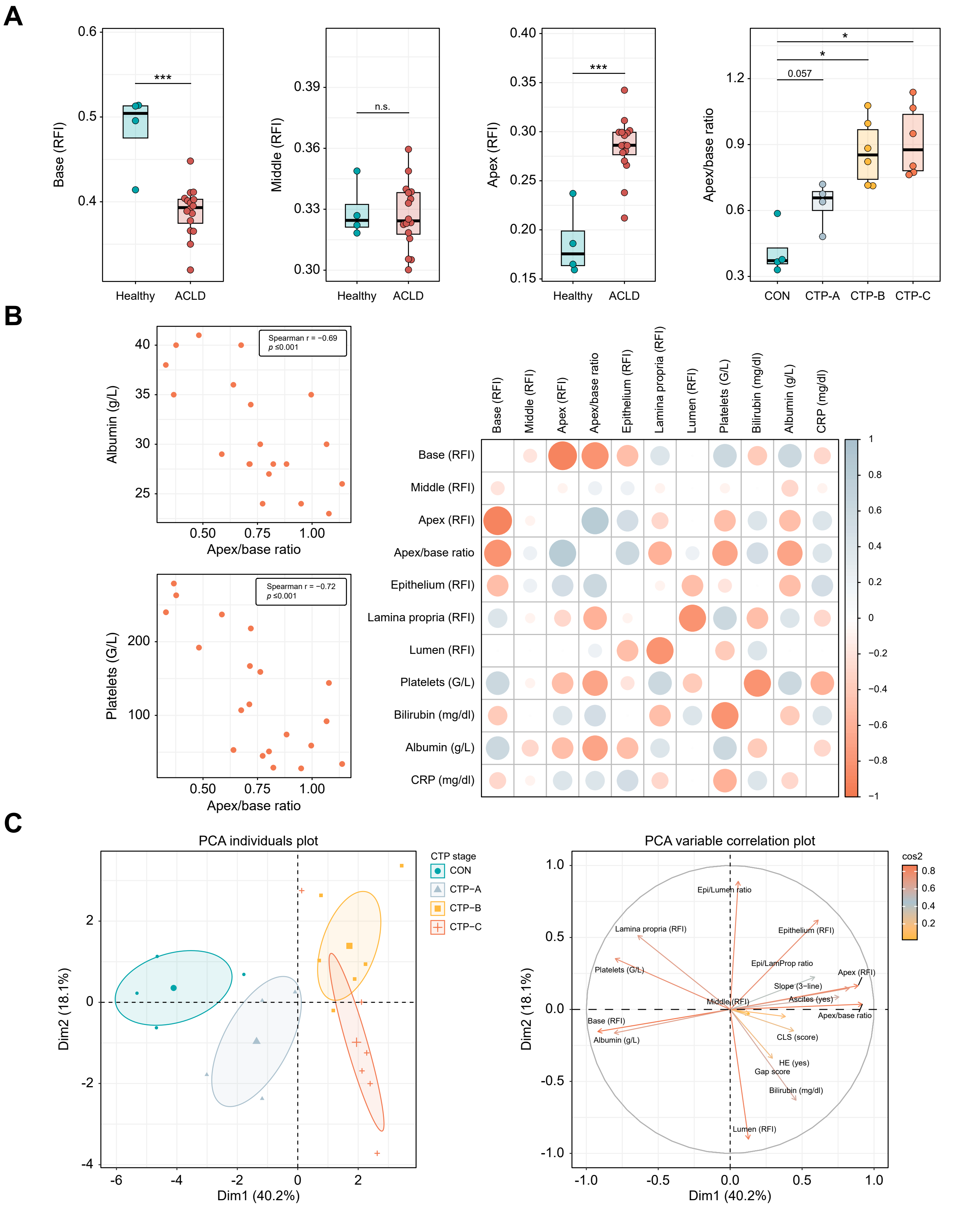

Fig. 3. Application of quantitative CLE analysis in the exploration cohort (Bern). (A) Three-line analysis of epithelial fluorescence intensity in the exploration cohort, including healthy controls. Group comparisons were performed using Wilcoxon rank-sum tests with Benjamini-Hochberg correction for multiple testing, where applicable. (B) Correlation matrix of CLE-derived and laboratory parameters and correlation plots of apex/base ratio with serum albumin and platelet levels. Correlations were evaluated using Spearman rank correlation. (C) Principal component analysis (PCA) integrating CLE-derived metrics with clinical and laboratory

Interestingly, apex/base ratio showed a strong negative correlation with serum albumin (r = -0.694, p <0.001) and platelet levels (r = -0.716, p <0.001), and a significant positive correlation with bilirubin (r = 0.478, p <0.001) denoting surrogates of liver function and portal hypertension, respectively.

PCA integrating clinical, laboratory, and CLE-derived parameters demonstrated separation of healthy controls from patients with ACLD, as well as stage-dependent clustering across CTP stages. Notably, variables derived from the quantitative 3-line/3-compartment CLE approaches were among the strongest contributors to the principal components differentiating patients (Fig. 3C and Fig. S4; Supplementary Results).

Validation of CLE-based intestinal permeability analysis in independent cohort

In the Vienna cohort, transepithelial fluorescein permeation into the gut lumen (qualitative assessment, following Watson grading system[13]) showed a stepwise increase across disease stages, being significantly more prevalent in decompensated cirrhosis: 24% of patients with compensated ACLD, 57% with first decompensation, and 71% with further decompensation (p = 0.019; Fig. S5). As expected, stratification by CTP stages yielded a similar observation: 32% in CTP-A, 57% in CTP-B, and 89% in CTP-C (p = 0.011; Fig. 4A). As demonstrated in Table 1 and Table S2, established clinical and laboratory parameters reflecting disease severity such as MELD and HVPG increased across mentioned disease stages, emphasizing the representativeness of cohort characteristics.

Application of the 3-line and 3-compartment analysis technique in the Vienna ACLD cohort confirmed external reproducibility of the Bern cohort results regarding its relationship with disease stages. Of note, one patient was excluded for 3-line/3-compartment analyses because of technical issues with white balance of images.

Apex/base ratio, apex/middle ratio, and 3-line slopes significantly increased across CTP stages (Fig. 4B and Fig. S6). These observations were paralleled by increasing lumen and decreasing lamina propria RFI (Fig. 4C), representing quantitative readouts of transepithelial fluorescein leakage and intestinal permeability.

Concordant trends for 3-line readouts were observed when stratifying patients into cACLD, first decompensation, and further decompensation; while comparison of 3-compartment readouts did not attain statistical significance (Fig. S6). Finally, 3-line/3-compartment readouts were also aligned with the presence *vs.* absence of ascites, representing a further aspect of independent validation across cohorts (Fig. S7). Results on Gap score and CLS showed similar trends in the validation cohort as observed in the exploration cohort (Fig. S8).

Correlation between CLE-based barrier dysfunction and relevant biomarkers in cirrhosis

Quantitative results from the 3-line and 3-compartment analysis were correlated with indicators of liver function and disease severity in the Vienna cohort. Most relevant findings are visualized by volcano plots (Fig. 5A and Fig. S9).

Apex/base ratio (r = 0.575, p <0.001) showed the strongest positive correlation to HVPG, and the strongest negative link to serum albumin (r = -0.553, p <0.001); while also correlating with MELD (r = 0.438, p <0.001), CTP score (r = 0.433, p <0.001), and platelet (PLT) count (r = -0.306, p <0.05). Similar correlation patterns and coefficients were observed for apex-middle ratio and the 3-layer slopes.

Furthermore, the link between CLE parameters and advanced serum biomarkers available in a patient subgroup were explored (available values indicated in Table S3). Importantly, 3-line parameters showed strong to moderate correlation with the Enhanced Liver Fibrosis (ELF®) score (apex/base: r = 0.584, apex/middle: r = 0.603, slope: r = 0.564), Von Willebrand Factor antigen (VWF) (apex/base: r = 0.431, apex/middle: r = 0.379, slope: r = 0.422), total serum bile acids (BA) (apex/base: r = 0.445, apex/middle: r = 0.533, slope: r = 0.430), and total IgM concentrations (apex/base: r = 0.416, apex/middle: r = 0.478, slope: r = 0.400; all p <0.05).

PCA demonstrated separation of patients according to Baveno clinical stages, with CLE-derived parameters among the strongest contributors to the principal components differentiating patients. Notably, variables derived from the novel quantitative CLE approaches were prominently represented among the top contributing features (Fig. S10; Supplementary Results).

CLE barrier function and prognosis

During a median transplant-free follow-up of 13.2 months (IQR 9.85–18.3 months) in the Vienna cohort, 10 patients (21%) experienced an adverse liver-related event (LRE; composite endpoint): further decompensation in seven patients and ACLF in three patients. Among patients experiencing LRE, three patients experienced SBP, and three patients progressed to liver-related death. Two patients were censored at liver transplantation. Exploratory analyses on the relationship between CLE and clinical outcome were performed.

Importantly, CLE-assessed barrier dysfunction as reflected by presence of luminal fluorescein leakage (*vs.* absence of leakage[13]) was associated with a higher incidence of adverse LRE (log-rank p = 0.003; 25% *vs.* 5% at 12 months; 43% *vs.* 5% at 18 months; Fig. S11).

Consequently, quantitative 3-line and 3-layer analyses also showed prognostic value for LRE. First, stratification by median quantitative readouts discriminated between patients with high *vs*. low risk for disease progression (Fig. 5B and Fig. S11). Second, Cox proportional hazard models (Table 2) showed that MFI in the lumen was linked to a higher LRE incidence (hazard ratio [HR] 1.02, 1.01–1.03, p = 0.002), while higher MFI in the lamina propria was linked to a lower risk (HR 0.95, 0.91-0.99, p = 0.017), denoting opposing trends consistent with transepithelial fluorescein shift into the intestinal lumen. For the 3-line method, apex/base ratio (HR 10.6, 1.71-66.0, p = 0.011), apex/middle ratio (HR 19.1, 2.10-173, p = 0.009), and 3-line

variables. PCA was performed on scaled variables (unit variance) using complete-case observations. Statistics legend: (ns) not significant; (*) p <0.05; (**) p <0.01; (***) p <0.001. CRP, C-reactive protein; CON, healthy controls; RFI, relative fluorescence intensity.

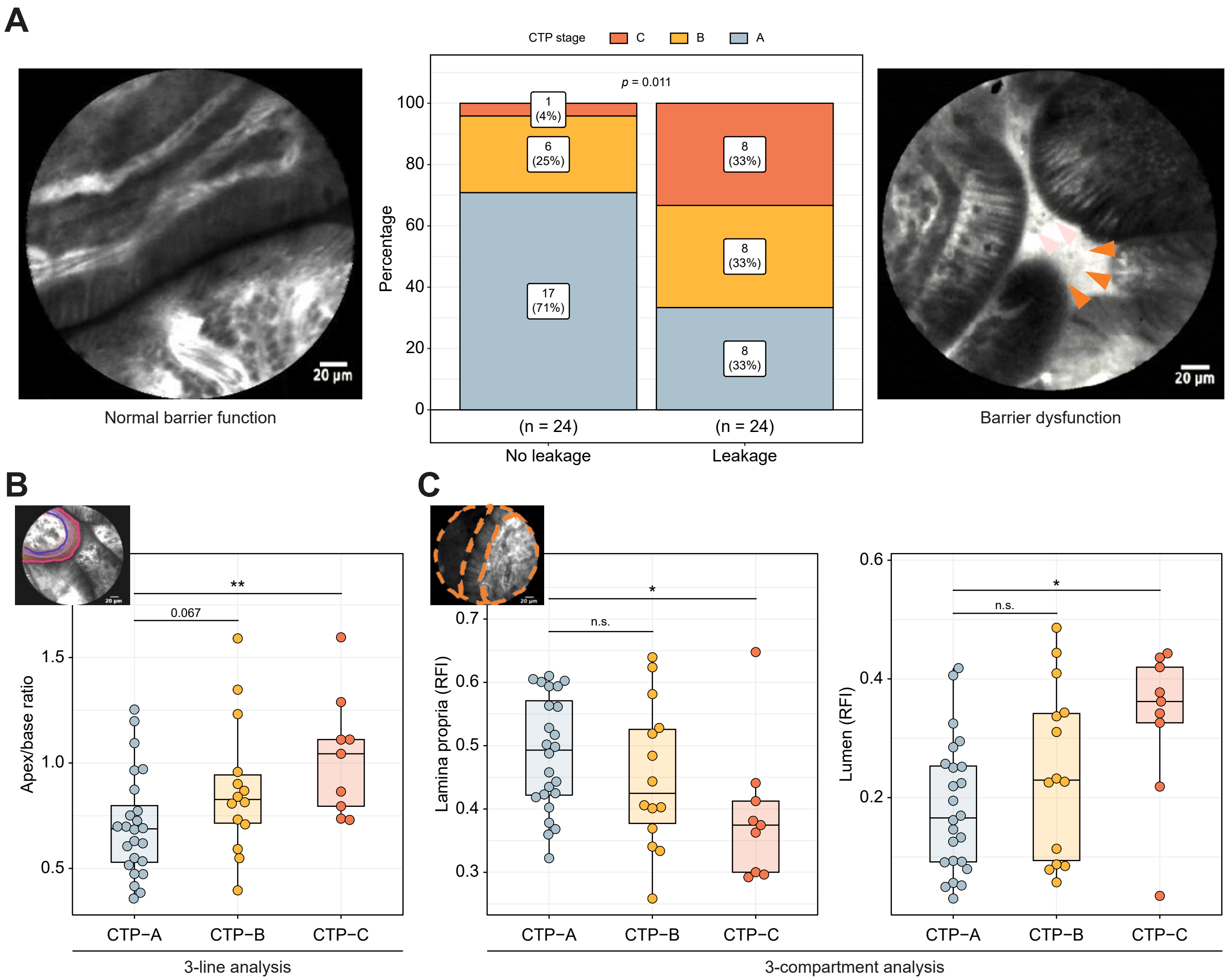

Fig. 4. Application of CLE in the validation cohort (Vienna). (A) Representative CLE images illustrating absent and present intraluminal fluorescein leakage. Leakage prevalence was compared across CTP stages using the X^2 test. (B) Three-line analysis of epithelial fluorescence intensity stratified by CTP stage. (C) Three-compartment analysis of fluorescence distribution across epithelial, lamina propria, and luminal compartments by CTP stage. Group comparisons were performed using Wilcoxon rank-sum tests with Benjamini–Hochberg correction for multiple testing. Statistics legend: (ns) not significant; (*) p <0.05; (**) p <0.01; (***) p <0.001. CTP, Child-Turcotte-Pugh, RFI, relative fluorescence intensity.

slopes (HR 1.05, 1.01-1.10, p = 0.013) correlated with LRE incidence during follow-up.

Importantly, established prognostic factors such as MELD (HR per point: 1.26, 1.12–1.41, p <0.001), albumin (HR per g/L: 0.87, 0.78–0.96, p = 0.006), and HVPG (HR per point: 1.13, 0.98-1.30, p = 0.081) were also linked to adverse outcomes in Cox regression models, supporting the plausibility and representativeness of CLE results. Multivariate models or subgroup analyses were not performed because of sample size considerations to avoid overfitting.

Interobserver variability of CLE readouts

To address interobserver reliability, images were independently analyzed by two blinded observers in the Bern and the Vienna cohorts, respectively. Interobserver variability was substantially higher for the qualitative scoring systems compared with the new quantitative 3-line/3-compartment approaches in both cohorts (Fig. 6). Among all assessed methods, the 3-line/3-compartment approach exhibited the best interobserver agreements, underscoring robustness and reproducibility. For example, the 3-line method showed excellent interobserver agreement in the Vienna cohort, including base (ICC = 0.95, 95% CI 0.91–0.97), middle (ICC = 0.97, 95% CI 0.95–0.98), and apex (ICC = 0.97, 95% CI 0.94–0.98).

Discussion

The present study shows that CLE enables direct, *in vivo*, high-resolution visualization of intestinal barrier dysfunction in patients with ACLD, and that quantitative CLE-derived measures indicating elevated permeability increase across disease stages. In exploratory analyses, these measures were additionally associated with portal hypertension markers, liver function, and liver-related outcomes. By integrating two

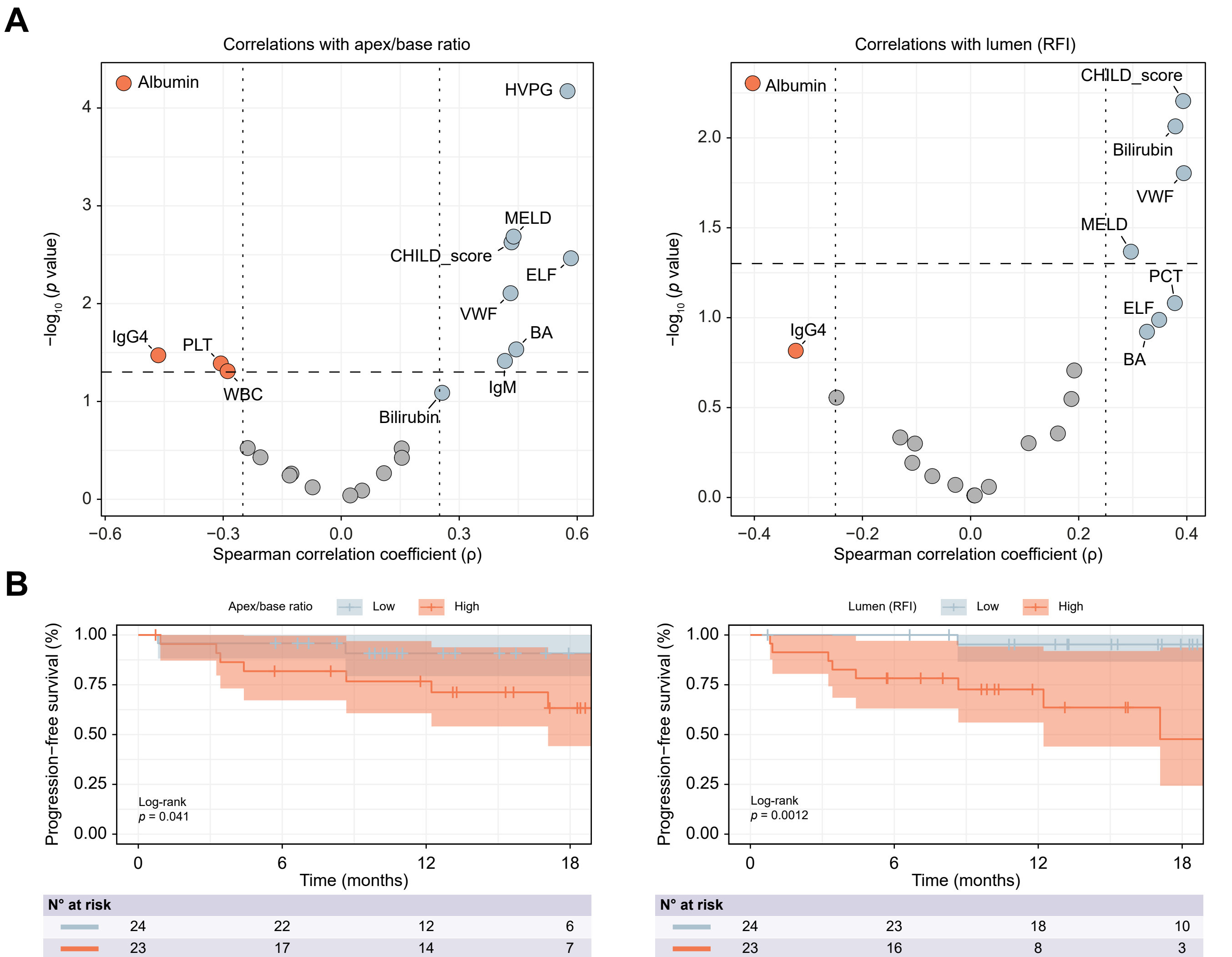

Fig. 5. Associations between CLE-derived parameters and clinical features, and their prognostic value for liver-related events. (A) Correlation analysis of CLE-derived parameters, (B) Incidence of liver-related events during the follow-up period after stratification by CLE parameters. (A) Volcano plots illustrating Spearman rank correlations between the CLE readouts and clinical as well as laboratory parameters. The x-axis shows the Spearman correlation coefficient (ρ) and the y-axis shows -$\log_{10}$(p value); dashed and dotted lines indicate nominal significance and effect size thresholds, respectively. (B) Kaplan–Meier curves indicating disease progression-free follow-up (composite endpoint: first/further decompensation, ACLF, and liver-related death) were compared by log-rank test. Groups are stratified by median CLE parameters. ACLF, acute-on-chronic liver failure; BA, serum bile acids; CHILD, Child-Pugh score, CLE, confocal laser endomicroscopy; ELF®, Enhanced Liver Fibrosis score, HVPG, hepatic venous pressure gradient, Ig, immunoglobulin, MELD, model for end-stage liver disease; PCT, procalcitonin; RFI, relative fluorescence intensity; VWF, von Willebrand factor; WBC, white blood cells.

independent cohorts from Bern and Vienna and introducing a novel quantitative analytical framework based on 3-line and 3-compartment analysis, this study extends previous CLE work in other diseases,[10–13,21] and in the context of cirrhosis,[22] and supports the clinical relevance of intestinal barrier permeability as a key feature of gut–liver axis impairment in ACLD.[23,24]

The gut–liver axis is now recognized as a key driver of cirrhosis progression, where intestinal barrier dysfunction promotes microbial translocation, systemic inflammation, and thereby predisposes to clinical complications such as hepatic decompensation, ACLF, and liver-related death.[25–28] The present results reinforce this concept by demonstrating that CLE-derived measures of intestinal permeability correlate closely with established markers of portal hypertension and liver dysfunction, including HVPG, albumin, MELD, and systemic inflammation biomarkers. Importantly, significant associations were consistently observed across two independent cohorts, underscoring the external validity and reproducibility of the findings. For example, 3-line parameters were robustly linked to disease stages, and key correlation results were validated, for example, the link between apex/base ratio and albumin, and surrogates for portal hypertension (HVPG/PLT count). Amelioration of portal hypertension may beneficially impact on intestinal (vascular) permeability via decreasing hydrostatic pressure within the splanchnic vasculature – a hypothesis that is also supported by observations that systemic inflammation ameliorates after transjugular intrahepatic portosystemic shunt.[29]

A major methodological advance of the present study is the implementation of a quantitative 3-line and 3-compartment CLE analysis. While earlier CLE-based approaches relied predominantly on qualitative or semi-

Table 2. Cox regression models for the prediction of adverse liver-related events.

Parameter	HR	95% CI	*p* value
Age (per year)	0.99	0.95–1.04	0.879
HVPG (per mmHg)	1.13	0.98–1.30	0.081
MELD (per point)	1.26	1.12–1.41	**<0.001**
Albumin (per g/L)	0.87	0.78–0.96	**0.006**
Luminal leakage (y)	11.9	1.50–95.0	**0.019**
Epithelium (MFI)	1.02	0.99–1.05	0.236
LamProp (MFI)	0.95	0.91–0.99	**0.017**
Lumen (MFI)	1.02	1.01–1.03	**0.002**
Apex/base ratio	10.6	1.71–66.0	**0.011**
Middle/base ratio	77.3	0.21–28,302	0.149
Apex/middle ratio	19.1	2.10–173	**0.009**
Slope	1.05	1.01–1.10	**0.013**
CLS	1.04	1.01–1.06	**0.006**
Gap score	3.00	0.717–12.529	0.133

Statistical analysis: univariable Cox regression analysis of factors associated with adverse LRE in the Vienna cohort: first/further decompensation, ACLF, and liver-related death (composite endpoint; n = 10 events). *p*-values below 0.05 are indicated in bold. CLS, Confocal Leak score; HR, hazard ratio; HVPG, hepatic venous pressure gradient; LRE, liver-related events; MELD, model for end-stage liver disease; MFI, mean fluorescence intensity.

quantitative grading of fluorescein leakage, the current framework enables a more granular and objective characterization of mucosal permeability across distinct anatomical layers. This quantitative read-out not only follows established methods of patient stratification (CTP stages, Baveno clinical stages, MELD, etc.), but also demonstrates stronger correlations with pathomechanistic biomarkers compared with conventional CLE scoring. The results indicate that the quantitative CLE metrics are robust and reproducible. Whether they are suited for other conditions associated with altered intestinal permeability remains to be tested in future studies. In its current form the quantitative analysis is performed offline and manually, which together with probe cost and the need for an experienced reader, currently limits its use to specialized research settings. Nevertheless, the strong interobserver agreement of the quantitative metrics suggests they are well suited for standardization and, if (semi-)automated image analysis would become available in the future, could be extended to broader application.

While CLE has been extensively studied as a tool to detect intestinal leakage in IBD and functional gastrointestinal disorders,[10–13,21] its application in ACLD has remained largely unexplored. In the present study, we observed a high prevalence of intestinal barrier dysfunction in ACLD, with overt fluorescein leakage into the gut lumen being present in approximately half of the patient cohort; importantly, being much more frequent in advanced (decompensated) cirrhosis stages. These findings are in line with accumulating evidence linking increased intestinal permeability to advanced portal hypertension and cirrhosis-related complications.[30]

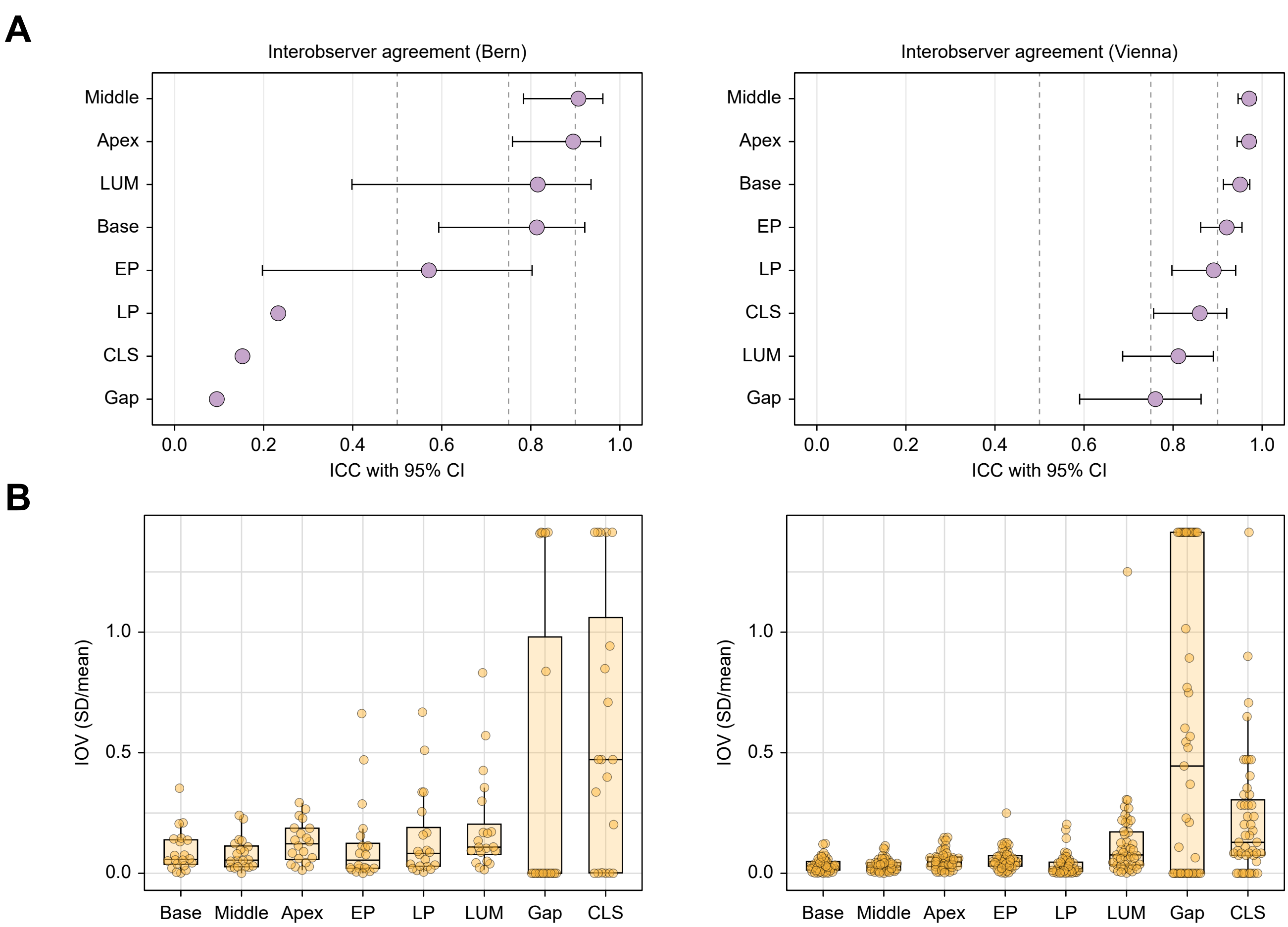

Fig. 6. Interobserver agreement and variability of CLE readouts. (A) Interobserver agreement was evaluated using ICC(2,1) (two-way random-effects, absolute agreement); error bars indicate 95% CIs. (B) Standard deviation was divided by the mean of two different reader evaluations. CLE, confocal laser endomicroscopy; CLS, Confocal Leak Score; EP, epithelium; Gap, Gap score; ICC, intraclass correlation coefficient; IOV, interobserver variability; LP, lamina propria; LUM, lumen.

Beyond cross-sectional associations, CLE-based assessment of intestinal barrier dysfunction showed prognostic relevance. Patients with severe CLE-derived permeability alterations experienced a significantly higher rate of adverse LRE, consistent with established prognostic models such as MELD or CTP scores. Besides binary classifications stratifying patients by risk of disease progression, the novel quantitative readouts showed proportional increase of hazard for disease progression with quantitative CLE variables, further emphasizing their clinical relevance. Notably, we observed an opposing trend of lamina propria and lumen fluorescence intensity regarding risk of disease progression in Cox regression analyses, likely representing a shift of fluorescein from the lamina propria to the gut lumen – in combination with apex/base ratio and slopes from the 3-line analysis – which emphasizes the plausibility of observed results. These exploratory observations suggest that CLE-derived metrics may carry prognostic information. However, given the limited number of events and the absence of multivariable adjustment, they should be regarded as hypothesis-generating rather than evidence of prognostic value independent of established markers such as MELD or HVPG.

Numerous experimental studies have deciphered mechanisms leading to intestinal barrier dysfunction in cirrhosis.[31–38] Current human studies investigating intestinal barrier dysfunction in ACLD have largely relied on indirect biomarkers such as circulating PAMPs, inflammatory mediators,[39–41] sugar absorption tests, or radioisotopes.[42,43] While informative, these surrogate markers often do not provide direct or quantitative insights into mucosal barrier permeability. Biopsy-based approaches (*e.g.* transepithelial electrical resistance and transcriptomic profiling[44,45]) enable mechanistic characterization but remain confined to highly specialized research settings. In contrast, CLE offers a direct, *in vivo* functional assessment during routine endoscopy, with quantitative analysis performed offline, representing a promising technique for translational research and clinical applications. Notably, CLE readouts predominantly reflect the paracellular/transepithelial permeability of the mucosa to a small solute (fluorescein, ∼330 Da). The stepwise apex/base gradient and the redistribution of fluorescence from the lamina propria to the lumen are consistent with loss of tight-junction integrity and increased convective transfer, in line with the observed correlations with albumin and HVPG. Thus, CLE should be regarded as a complementary functional read-out *in vivo* rather than competing with techniques dissecting molecular mechanisms.

Although the ileum is traditionally considered the primary site of bacterial translocation,[4,9,46] our data suggest that CLE assessment of the small intestinal mucosa in the duodenum also yields clinically relevant information on intestinal barrier dysfunction in ACLD. Given that patients with ACLD commonly undergo routine upper gastrointestinal endoscopy for variceal screening or treatment according to current guidelines,[14,16] CLE could be integrated into existing workflows. This contrasts with colonoscopy-based CLE, as colonoscopy may be less frequently performed in ACLD but more commonly in IBD.[47]

From a broader pathophysiological perspective, the direct visualization of (intravenously injected) fluorescein leakage into the intestinal lumen by CLE may also revive earlier concepts linking chronic liver disease to intestinal protein loss, such as increased fecal albumin excretion described decades ago.[48] Recent experimental and translational studies have revisited these mechanisms, further supporting a central role of intestinal barrier dysfunction in ACLD progression.[49,50] In this context, the strong correlations between apex/base ratio and albumin (negative correlation) and HVPG (positive correlation) should be noted and further investigated in future studies.

The present study has notable strengths. First, the inclusion of two independent cohorts, two independent readers for each center, and a strong interobserver agreement for the novel analysis methods enhances generalizability and preemptively addresses concerns of reader/center-specific bias. Second, the introduction of a quantitative, compartment-based CLE analysis represents a substantial methodological advance with suitable interobserver reproducibility. Third, the approach yields objective metrics that are closely linked to clinically and pathophysiologically relevant parameters, supporting their translational relevance.

Nevertheless, we acknowledge limitations. First, despite the inclusion of two independent cohorts, sample size remains moderate, particularly for subgroup and outcome analyses. Larger prospective studies are required to confirm these findings, to refine prognostic cut-offs, and to evaluate whether CLE-based barrier assessment adds incremental value beyond established risk models. Second, CLE readouts were not adjusted for medications that may influence intestinal permeability and co-vary with disease severity (*e.g.* non-selective beta-blockers, rifaximin) because of the limited sample size. Finally, the correlation between CLE results and certain biomarkers including HVPG could not be cross-validated in the Bern cohort, as a result of different clinical work-up between centers.

Future studies could determine whether therapeutic interventions directed at gut barrier function translate into improved clinical outcomes; and investigate if CLE results correlate with molecular readouts from mucosa biopsies such as tight-junction expression and immune cell activation. We note that for the latter point, no established treatment improving gut barrier integrity in ACLD is yet approved for clinical practice, but should be considered for future clinical trials. For example, despite challenges in clinical implementation,[51] pharmacological agents targeting BA signaling improved intestinal barrier integrity and ameliorated chronic liver disease in experimental studies.[31]

In summary, CLE enables direct, *in vivo* visualization and quantitative assessment of intestinal barrier dysfunction in patients with ACLD. The newly introduced 3-line and 3-compartment analyses provide robust, reproducible metrics that closely reflect disease severity, portal hypertension, and clinical outcomes. Further prospective and translational studies are warranted to validate findings, link CLE to molecular mechanisms of gut barrier integrity, and explore further interventional strategies targeting the gut–liver axis in cirrhosis.

Affiliations

[1]*Division of Gastroenterology and Hepatology, Department of Medicine III, Medical University of Vienna, Vienna, Austria;* [2]*Vienna Hepatic Hemodynamic Lab, Division of Gastroenterology and Hepatology, Department of Medicine III, Medical University of Vienna, Vienna, Austria;* [3]*Clinical Research Group MOTION, Medical University of Vienna, Vienna, Austria;* [4]*Christian Doppler Laboratory for Portal Hypertension and Liver Fibrosis, Medical University of Vienna, Vienna, Austria;* [5]*CeMM Research*

Center for Molecular Medicine of the Austrian Academy of Sciences, Vienna, Austria; [6]Department of Disease Biology, Molecular Cell Biology Group, Almirall, Sant Feliu de Llobregat, Spain; [7]Department of Agri-Food Engineering and Biotechnology, Universitat Politècnica de Catalunya (UPC), Castelldefels, Sant Feliu de Llobregat, Spain; [8]Department of Visceral Surgery and Medicine, Inselspital, Bern University Hospital, University of Bern, Bern, Switzerland; [9]Department for BioMedical Research, Visceral Surgery and Medicine, University of Bern, Bern, Switzerland; [10]Department of Bioengineering, School of Engineering, Universitat Ramon Llull - Institut Químic de Sarrià (URL-IQS), Barcelona, Spain

Abbreviations

ACLD, advanced chronic liver disease; ACLF, acute-on-chronic liver failure; ALD, alcohol-related liver disease; BA, bile acid; cACLD, compensated ACLD; CLE, confocal laser endomicroscopy; CLS, Confocal Leak score; CRP, C-reactive protein; CTP, Child-Turcotte-Pugh; dACLD, decompensated ACLD; FI, fluorescence intensity; HE, hepatic encephalopathy; HR, hazard ratio; HRS-AKI, hepatorenal syndrome–acute kidney injury; HVPG, hepatic venous pressure gradient; IBD, inflammatory bowel disease; ICC, intraclass correlation coefficient; ISO, International Organization for Standardization; LRD, liver-related death; LRE, liver-related event; MASLD, metabolic dysfunction-associated liver disease; MELD, model for end-stage liver disease; MFI, mean fluorescence intensity; PAMPs, pathogen-associated molecular patterns; PC1, principal component 1; PC2, principal component 2; PCA, principal component analysis; PLT, platelet count; RFI, relative fluorescence intensity; SBP, spontaneous bacterial peritonitis.

Financial support

The study was supported by the Clinical Research Group MOTION, Medical University of Vienna, Vienna, Austria – a project funded by the Clinical Research Groups Programme of the Ludwig Boltzmann Gesellschaft (Grant Nr: LBG_KFG_22_32) with funds from the Fonds Zukunft Österreich.

Conflicts of interest

BS has received travel support from AbbVie, Gilead, Falk, and Mirum.

MM served as a speaker and/or consultant and/or advisory board member for AbbVie, AstraZeneca, Echosens, Eli Lilly, Gilead, Ipsen, Takeda, and W. L. Gore & Associates and received grant support from Echosens as well as travel support from AbbVie and Gilead. MT received grant support from Albireo, Alnylam, Cymabay, Falk, Genentech, Gilead, Intercept, MSD, Takeda, and UltraGenyx, honoraria for consulting from AbbVie, Albireo, Agomab, Boehringer Ingelheim, BiomX, Chemomab, Dexoligo Therapeutics, Falk, Genfit, Gilead, GSK, Hightide, Intercept, Ipsen, Janssen, MSD, Novartis, Phenex, Pliant, Regulus, Siemens, and Shire, speaker fees from Albireo, Boehringer Ingelheim, Bristol-Myers Squibb, Falk, Gilead, Ipsen, Intercept, MSD, and Madrigal, as well as travel support from AbbVie, Falk, Gilead, Janssen, and Intercept. He is also co-inventor of patents on the medical use of 24-norursodeoxycholic acid filed by the Medical University of Graz. TR received grant support from Abbvie, Boehringer Ingelheim, Gilead, Intercept/Advanz Pharma, MSD, Myr Pharmaceuticals, Philips Healthcare, Pliant, Siemens, and W. L. Gore & Associates; speaking honoraria from Abbvie, Echosens, Gilead, Intercept/Advanz Pharma, Roche, MSD, and W. L. Gore & Associates; consulting/advisory board fee from Abbvie, Astra Zeneca, Bayer, Boehringer Ingelheim, Gilead, Intercept/Advanz Pharma, MSD, Resolution Therapeutics, and Siemens; and travel support from Abbvie, Boehringer Ingelheim, Dr. Falk Pharma, Gilead, and Roche. The remaining authors declare no conflict of interest.

Please refer to the accompanying ICMJE disclosure forms for further details.

Authors' contributions

Study concept and design: MS, RW, JH, BS, TR. Data acquisition: all authors. Data analysis: MS, RW, JH, BS, TR. Data interpretation: all authors. Drafted the manuscript: BS, MS. All authors of the manuscript: JS, MF, JC, SGR, MM, AB, MT, TR, JH, RW. Approved the final version of the manuscript: all authors.

Data availability

Data are available at reasonable request to the corresponding author.

Declaration of generative AI and AI-assisted technologies in the writing process

During the preparation of this work the authors used ChatGPT 5.0 (OpenAI) to maximize readability, eloquence and grammatical correctness. After using this tool, the authors reviewed and edited the content as needed and take full responsibility for the content of the publication.

Supplementary data

Supplementary data to this article can be found online at https://doi.org/10.1016/j.jhepr.2026.102037.

References

Author names in bold designate shared co-first authorship

[1] Albillos A, de Gottardi A, Rescigno M. The gut-liver axis in liver disease: pathophysiological basis for therapy. J Hepatol 2020;72:558–577.

[2] Fritz E, Hammer J. Gastrointestinal symptoms in patients with liver cirrhosis are linked to impaired quality of life and psychological distress. Eur J Gastroenterol Hepatol 2009;21:460–465.

[3] Pabst O, Hornef MW, Schaap FG, et al. Gut-liver axis: barriers and functional circuits. Nat Rev Gastroenterol Hepatol 2023;20:447–461.

[4] Simbrunner B, Trauner M, Reiberger T. Therapeutic aspects of bile acid signalling in the gut-liver axis. Aliment Pharmacol Ther 2021;54:1243–1262.

[5] Costa D, Trebicka J, Ripoll C, et al. Interaction of inflammation and portal hypertension in cirrhosis progression. Nat Rev Gastroenterol Hepatol 2025;22:846–865.

[6] **Simbrunner B**, **Caparrós E**, Neuwirth T, et al. Bacterial translocation occurs early in cirrhosis and triggers a selective inflammatory response. Hepatol Int 2023;17:1045–1046.

[7] Gómez-Hurtado I, Gallego-Durán R, Zapater P, et al. Bacterial antigen translocation and age as BMI-independent contributing factors on systemic inflammation in NAFLD patients. Liver Int 2020;40:2182–2193.

[8] Bellot P, García-Pagán JC, Francés R, et al. Bacterial DNA translocation is associated with systemic circulatory abnormalities and intrahepatic endothelial dysfunction in patients with cirrhosis. Hepatology 2010;52:2044–2052.

[9] Simbrunner B, Hofer BS, Schwabl P, et al. FXR-FGF19 signaling in the gut–liver axis is dysregulated in patients with cirrhosis and correlates with impaired intestinal defence. Hepatol Int 2024;18:929–942.

[10] Neumann H, Vieth M, Atreya R, et al. Assessment of Crohn's disease activity by confocal laser endomicroscopy. Inflamm Bowel Dis 2012;18:2261–2269.

[11] Moussata D, Goetz M, Gloeckner A, et al. Confocal laser endomicroscopy is a new imaging modality for recognition of intramucosal bacteria in inflammatory bowel disease *in vivo*. Gut 2011;60:26–33.

[12] Lim LG, Neumann J, Hansen T, et al. Confocal endomicroscopy identifies loss of local barrier function in the duodenum of patients with Crohn's disease and ulcerative colitis. Inflamm Bowel Dis 2014;20:892–900.

[13] Kiesslich R, Duckworth CA, Moussata D, et al. Local barrier dysfunction identified by confocal laser endomicroscopy predicts relapse in inflammatory bowel disease. Gut 2012;61:1146–1153.

[14] Mandorfer M, Aigner E, Cejna M, et al. Austrian consensus on the diagnosis and management of portal hypertension in advanced chronic liver disease (Billroth IV). Wiener klinische Wochenschrift 2023;135:493–523.

[15] European Association for the Study of the Liver. EASL Clinical Practice Guidelines for the management of patients with decompensated cirrhosis. J Hepatol 2018;69:406–460.

[16] de Franchis R, Bosch J, Garcia-Tsao G, et al. Baveno VII - Renewing consensus in portal hypertension. J Hepatol 2022;76:959–974.

[17] Chang J, Leong RW, Wasinger VC, et al. Impaired intestinal permeability contributes to ongoing bowel symptoms in patients with inflammatory bowel disease and mucosal healing. Gastroenterology 2017;153:723–731.e1.

[18] Turcotte JF, Kao D, Mah SJ, et al. Breaks in the wall: increased gaps in the intestinal epithelium of irritable bowel syndrome patients identified by confocal laser endomicroscopy (with videos). Gastrointest Endosc 2013;77:624–630.

[19] **Simbrunner B**, **Semmler G**, Bofill Roig M, et al. Stage-specific drivers of clinical progression in advanced chronic liver disease. Clin Gastroenterol Hepatol 2026. https://dx.doi.org/10.1016/j.cgh.2026.06.011. S1542-3565(26)00445-3. Online ahead of print.

[20] European Association for the Study of the Liver. EASL Clinical Practice Guidelines on acute-on-chronic liver failure. J Hepatol 2023;79:461–491.

[21] Fritscher-Ravens A, Schuppan D, Ellrichmann M, et al. Confocal endomicroscopy shows food-associated changes in the intestinal mucosa of patients with irritable bowel syndrome. Gastroenterology 2014;147:1012. 20.e4.
[22] Rusticeanu MA, Zimmer V. Alterations in intestinal mucosal barrier visualized by confocal laser endomicroscopy in liver cirrhosis: a pilot trial (AMBIC). Diagnostics 2024;14:1606.
[23] Wiest R, Albillos A, Trauner M, et al. Targeting the gut-liver axis in liver disease. J Hepatol 2017;67:1084–1103.
[24] Chopyk DM, Grakoui A. Contribution of the intestinal microbiome and gut barrier to hepatic disorders. Gastroenterology 2020;159:849–863.
[25] Tranah TH, Edwards LA, Schnabl B, et al. Targeting the gut-liver-immune axis to treat cirrhosis. Gut 2021;70:982–994.
[26] **Costa D**, **Simbrunner B**, Jachs M, et al. Systemic inflammation increases across distinct stages of advanced chronic liver disease and correlates with decompensation and mortality. J Hepatol 2021;74:819–828.
[27] Simbrunner B, Hartl L, Jachs M, et al. Dysregulated biomarkers of innate and adaptive immunity predict infections and disease progression in cirrhosis. JHEP Rep 2023;5:100712.
[28] Rodrigues SG, van der Merwe S, Krag A, et al. Gut-liver axis: pathophysiological concepts and medical perspective in chronic liver diseases. Semin Immunol 2024;71:101859.
[29] Kornfehl A, Tiede A, Hemetsberger P, et al. Decreasing interleukin-6 levels after TIPS predict outcomes in decompensated cirrhosis. JHEP Rep 2025;7:101308.
[30] Arroyo V, Angeli P, Moreau R, et al. The systemic inflammation hypothesis: towards a new paradigm of acute decompensation and multiorgan failure in cirrhosis. J Hepatol 2021;74:670–685.
[31] Sorribas M, Jakob MO, Yilmaz B, et al. FXR modulates the gut-vascular barrier by regulating the entry sites for bacterial translocation in experimental cirrhosis. J Hepatol 2019;71:1126–1140.
[32] Schwabl P, Hambruch E, Seeland BA, et al. The FXR agonist PX20606 ameliorates portal hypertension by targeting vascular remodelling and sinusoidal dysfunction. J Hepatol 2017;66:724–733.
[33] Gómez-Hurtado I, Moratalla A, Moya-Pérez Á, et al. Role of interleukin 10 in norfloxacin prevention of luminal free endotoxin translocation in mice with cirrhosis. J Hepatol 2014;61:799–808.
[34] Muñoz L, Borrero MJ, Úbeda M, et al. Intestinal immune dysregulation driven by dysbiosis promotes barrier disruption and bacterial translocation in rats with cirrhosis. Hepatology 2019;70:925–938.
[35] Verbeke L, Farre R, Verbinnen B, et al. The FXR agonist obeticholic acid prevents gut barrier dysfunction and bacterial translocation in cholestatic rats. Am J Pathol 2015;185:409–419.
[36] Teltschik Z, Wiest R, Beisner J, et al. Intestinal bacterial translocation in rats with cirrhosis is related to compromised Paneth cell antimicrobial host defense. Hepatology 2012;55:1154–1163.
[37] Hassan M, Moghadamrad S, Sorribas M, et al. Paneth cells promote angiogenesis and regulate portal hypertension in response to microbial signals. J Hepatol 2020;73:628–639.
[38] Mouries J, Brescia P, Silvestri A, et al. Microbiota-driven gut vascular barrier disruption is a prerequisite for non-alcoholic steatohepatitis development. J Hepatol 2019;71:1216–1228.
[39] Caro E, Francés R, Zapater P, et al. Grade of soluble inflammatory response is mainly affected by circulating bacterial DNA concentrations in cirrhosis. Liver Int 2016;36:1473–1480.
[40] Rodríguez-Laiz GP, Zapater P, Melgar P, et al. Bacterial DNA translocation contributes to systemic inflammation and to minor changes in the clinical outcome of liver transplantation. Sci Rep 2019;9:835.
[41] Reiberger T, Ferlitsch A, Payer BA, et al. Non-selective betablocker therapy decreases intestinal permeability and serum levels of LBP and IL-6 in patients with cirrhosis. J Hepatol 2013;58:911–921.
[42] Kalaitzakis E, Johansson JE, Bjarnason I, et al. Intestinal permeability in cirrhotic patients with and without ascites. Scand J Gastroenterol 2006;41:326–330.
[43] Wang L, Llorente C, Hartmann P, et al. Methods to determine intestinal permeability and bacterial translocation during liver disease. J Immunol Methods 2015;421:44–53.
[44] Bloom PP, Rao K, Bassis C, et al. Regional changes in intestinal permeability in cirrhosis are associated with mucosal bacteria. Hepatol Commun 2023;7:e0221.
[45] Smets L, Viola MF, Boesch M, et al. Intestinal blood vessel-associated macrophages and gut-vascular barrier dysfunction in cirrhosis. Gut 2026;75:1934–1949.
[46] **Fuchs CD**, **Simbrunner B**, Baumgartner M, et al. Bile acid metabolism and signaling in liver disease. J Hepatol 2025;82:134–153.
[47] Murthy SK, Feuerstein JD, Nguyen GC, et al. AGA clinical practice update on endoscopic surveillance and management of colorectal dysplasia in inflammatory bowel diseases: expert review. Gastroenterology 2021;161:1043. 51.e4.
[48] Olarte JA, Fernandez FJ, Sachetto JR, et al. Fecal excretion of I-131 labelled human serum albumin (RIHSA) in liver cirrhosis. Gastroenterologia 1964;102:159–162.
[49] Ubeda M, Lario M, Munoz L, et al. Obeticholic acid reduces bacterial translocation and inhibits intestinal inflammation in cirrhotic rats. J Hepatol 2016;64:1049–1057.
[50] Maccioni L, Gao B, Leclercq S, et al. Intestinal permeability, microbial translocation, changes in duodenal and fecal microbiota, and their associations with alcoholic liver disease progression in humans. Gut Microbes 2020;12:1782157.
[51] **Simbrunner B**, **Paternostro R**, Reiberger T, et al. Bile acid signaling in MASLD: from pathogenesis to therapeutic applications. Hepatology 2025. https://doi.org/10.1097/HEP.0000000000001539. Online ahead of print.

Keywords: Gut barrier; Cirrhosis; Gut–liver axis; Endoscopy; Confocal laser microendoscopy.
Received 12 June 2026; received in revised form 10 August 2026; accepted 5 September 2026; Available online 18 September 2026

Journal of Hepatology, Volume *8*

Supplemental information

Intestinal barrier dysfunction assessed by confocal laser endomicroscopy correlates with cirrhosis severity and clinical outcomes

Benedikt Simbrunner, Marcel Sorribas, Jessica Schaerer, Marco Felber, Jordi Codinach, Susana Gomes Rodrigues, Mattias Mandorfer, Annalisa Berzigotti, Michael Trauner, Thomas Reiberger, Johann Hammer, and Reiner Wiest

Intestinal barrier dysfunction assessed by confocal laser endomicroscopy correlates with cirrhosis severity and clinical outcomes

Benedikt Simbrunner, Marcel Sorribas, Jessica Schaerer, Marco Felber, Jordi Codinach, Susana Gomes Rodrigues, Mattias Mandorfer, Annalisa Berzigotti, Michael Trauner, Thomas Reiberger, Johann Hammer, Reiner Wiest

Table of contents

Supplementary methods......2
Supplementary results......4
Supplementary figures......5
Supplementary tables......18
Supplementary references......21

Supplementary methods

3-line method

Fluorescence intensity (FI, “grey value”) within the epithelial layer was measured at three predefined levels along the epithelial lining – base, middle, and apex - using Image J **(Figure-2B)**. For each line, pixel-wise grey values were averaged to obtain the mean fluorescence intensity (MFI). The relative fluorescence intensity (RFI) of each epithelial level was calculated as the proportion of its MFI relative to the sum of MFIs across all three lines which was defined as 100%. Ratios between the three epithelial levels were calculated based on the assumption that the amount of fluorescein determines fluorescent intensity as it permeates from the basal to the apical epithelial layers. Accordingly, a higher apex-to-base ratio was interpreted as an indirect marker of increased fluorescein permeation across the epithelium, reflecting epithelial permeability in the assessed segment. In addition, fluorescence intensity slopes (‘3-line slopes’) were calculated for each subject by fitting a linear regression across the three anatomical layers (base, middle, apex) using their respective MFIs. The resulting regression coefficient was used as a quantitative measure of the FI slope.

Confocal leak score (CLS)

CLS was assessed as previously described by Chang et al [1]. The CLS scale ranges from 0 (representing no intestinal permeability) to 100 (representing complete loss of barrier function). Accordingly, three features were assessed **(Fig. S12)**: **(A)** cell junction enhancement (demonstrating fluorescein enhancement between epithelial cells assumed to be due to loss of inter-cellular connectivity, e.g. diminished tight junctions (TJ)/function), **(B)** fluorescein leak (an outflow of fluorescein from the submucosal space into the lumen through epithelial breaks), and **(C)** cell drop out (active shedding of one or multiple enterocytes leading to an epithelial gap). Each image was evaluated for these features, and the CLS was determined by dividing the number of images containing one or more of the features by the total number of images per patient, then multiplying that number by 100. This resulted in a score ranging from 0 (representing no barrier dysfunction) to 100 (representing complete barrier dysfunction).

Gap score

Epithelial gap density was determined as previously described [2]. In brief, the gap density was derived by normalizing the total number of epithelial gaps (as exemplified in **Fig. S12D**) per 1000 epithelial cells counted. In this study the perimeter of the epithelial layer was used as a surrogate marker for the number of epithelial cells.

Statistical analysis

Categorical variables are reported as absolute (n) and relative (%) frequencies, while continuous variables are displayed as mean ± standard error of the mean or median with interquartile range (IQR), as appropriate. Group comparisons of categorical variables were performed using Chi squared or Fisher's exact test.

Principal component analysis (PCA) was performed using the 'FactoMineR' package, with centered and scaled variables. PCA biplots, scree plots, variable contribution plots, and individual factor maps were generated ('factoextra' package). Patients were additionally visualized according to disease stage using color-coded individual factor maps with confidence ellipses.

The level of significance was set at a two-sided p-value <0.05 for all analyses.

Supplementary results

CLE results - Bern cohort

To compare the new quantitative CLE readouts against previously established scores, **CLS** and **Gap Score (Fig. S3)** were assessed. The CLS demonstrated a stepwise numerical increase from healthy controls to patients with more advanced cirrhosis, with mean CLS values rising from 9.41 ± 8.54 in controls to 20.3 ± 8.97 in CTP-A, 20.0 ± 15.1 in CTP-B, and 25.6 ± 7.27 in CTP-C (p=0.184). The Gap Score remained within the reported normal range in healthy controls and showed few increased scores in patients with ACLD (p=0.595).

PCA was performed to assess the relative contribution of the different parameters to the overall variance. The analysis incorporated clinical and laboratory markers, CLS and Gap Score, as well as 3-line/-compartment CLE parameters. The PCA yielded two principal components explaining 59% of the total variance, with Dimensional Axis 1 (PC1) accounting for 39.9% and Dimensional Axis 2 (PC2) for 18.6% of the variability **(Figure-3C; Fig. S4)**. Healthy controls formed a clearly distinct cluster, indicating strong separation from patients with ACLD. In contrast, patients with ACLD exhibited closer spatial proximity but showed separation according to disease severity (CTP stages). Analysis of variable loadings revealed that novel CLE parameters were strong contributors to PC1 and PC2. Specifically, 5 of the 8 variables with the highest loadings on PC1 originated from 3-line/-compartment analyses, including the apex/base ratio. Similarly, 4 of the 5 top contributors to PC2 were derived from CLE readouts, underscoring their relevance in capturing variance related to intestinal barrier dysfunction in cirrhosis.

CLE results - Vienna cohort

PCA was performed in the Vienna cohort to explore multivariate patterns across clinical variables stratified by Baveno clinical stages **(Fig. S10)** [3]. The first two principal components explained 53.8% of the total variance, with PC1 accounting for 33.6% and PC2 for 20.2%. PC1 was predominantly driven by variables derived from the novel CLE-based measurement technique, with 6 of the 9 highest contributing variables originating from CLE parameters. PC2 was likewise strongly determined by CLE-derived variables, with 3 of the 5 strongest contributors originating from the new 3-line/-compartment approach. In the PCA score plot, patients demonstrated separation along PC1 and PC2 according to Baveno clinical stages, indicating that CLE-derived parameters capture clinically relevant variance associated with clinical disease stages.

Supplementary figures

Fig. S1. Quantitative epithelial 3-line analysis (Bern cohort): (A) comparison of base-middle-apex, (B/C) comparison between disease stages.

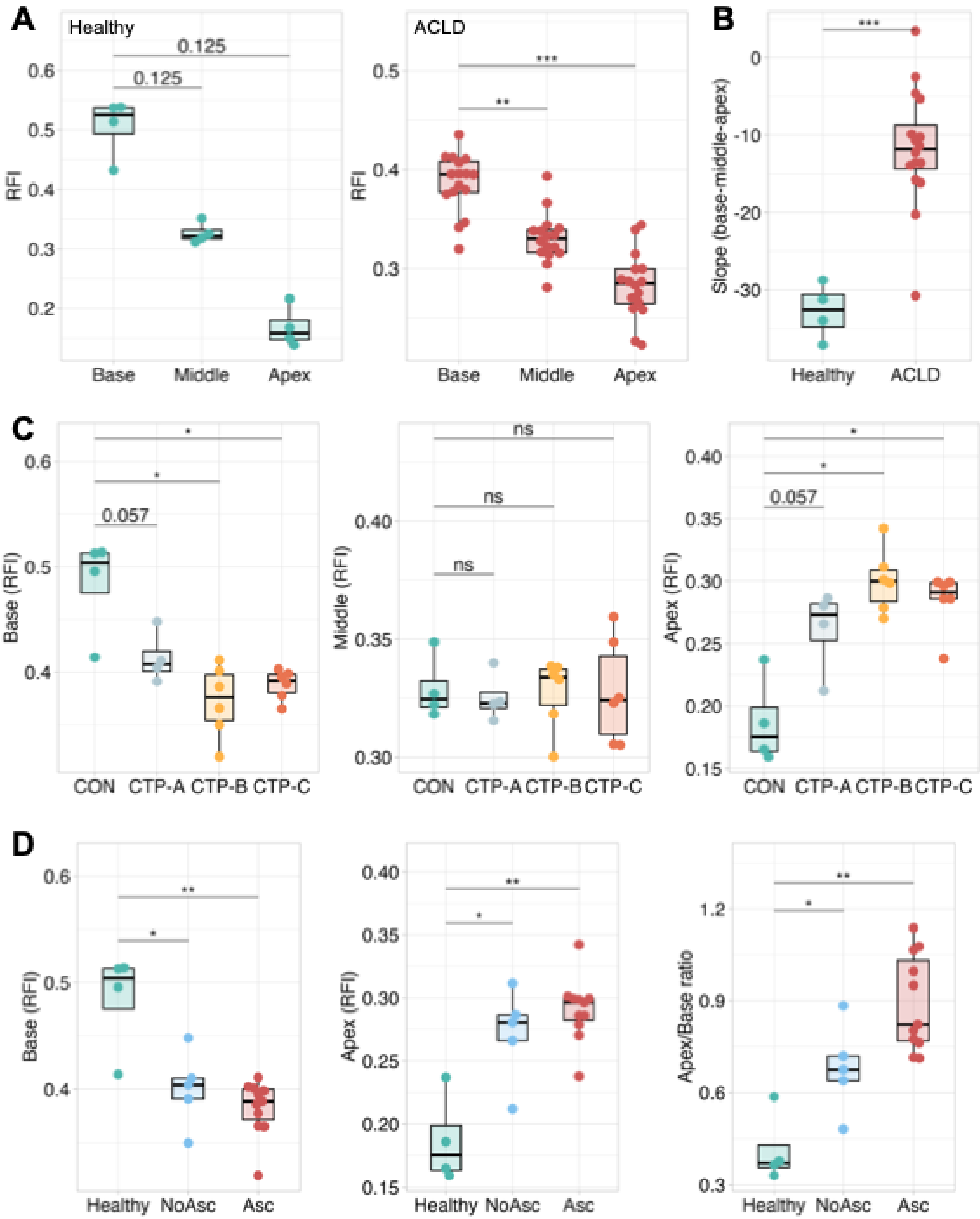

Statistics: Group comparisons were performed using pairwise Wilcoxon rank-sum tests versus the first group (Base; Healthy, CON) with Benjamini–Hochberg correction for multiple testing. Figure legend: (ns) not significant; (*) $p<0.05$; (**) $p<0.01$; (***) $p<0.001$. Abbreviations: (RFI) relative fluorescence intensity; (NoAsc) no ascites; (Asc) ascites; (CON) healthy controls, (CTP) Child-Turcotte-Pugh stage.

Fig. S2. Quantitative epithelial 3-compartment analysis (Bern cohort): (A) RFI between 3 compartments, (B/C) comparison between disease stages.

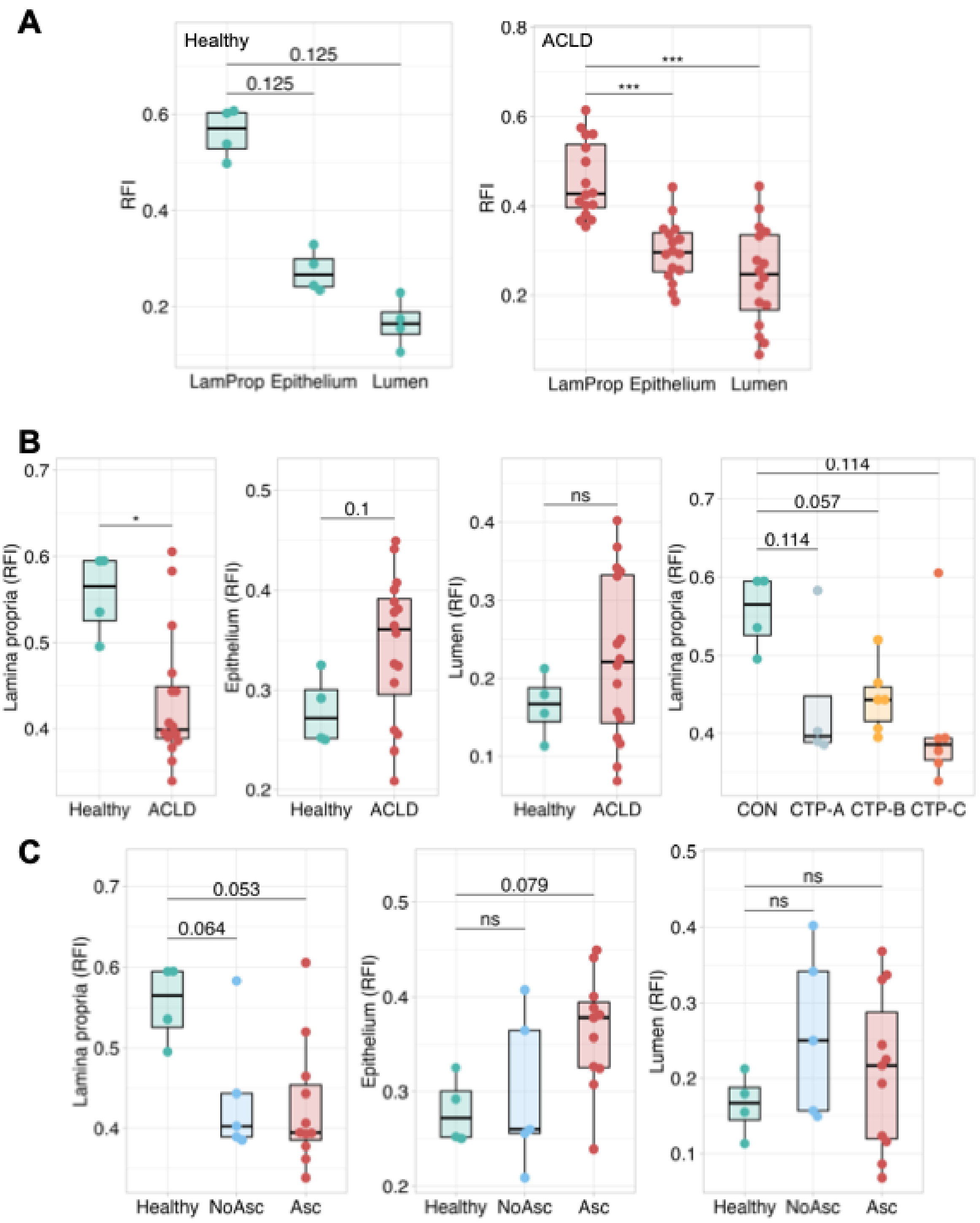

Statistics: Group comparisons were performed using pairwise Wilcoxon rank-sum tests versus the first group (Base; Healthy, CON) with Benjamini–Hochberg correction for multiple testing. Figure legend: (ns) not significant; (*) p<0.05; (**) p<0.01; (***) p<0.001. Abbreviations: (ACLD) advanced chronic liver disease; (RFI) relative fluorescence intensity; (NoAsc) no ascites; (Asc) ascites; (CON) healthy controls, (CTP) Child-Turcotte-Pugh stage.

Fig. S3. Gap and Confocal Leak Score (Bern cohort): comparison between healthy controls and patients with ACLD.

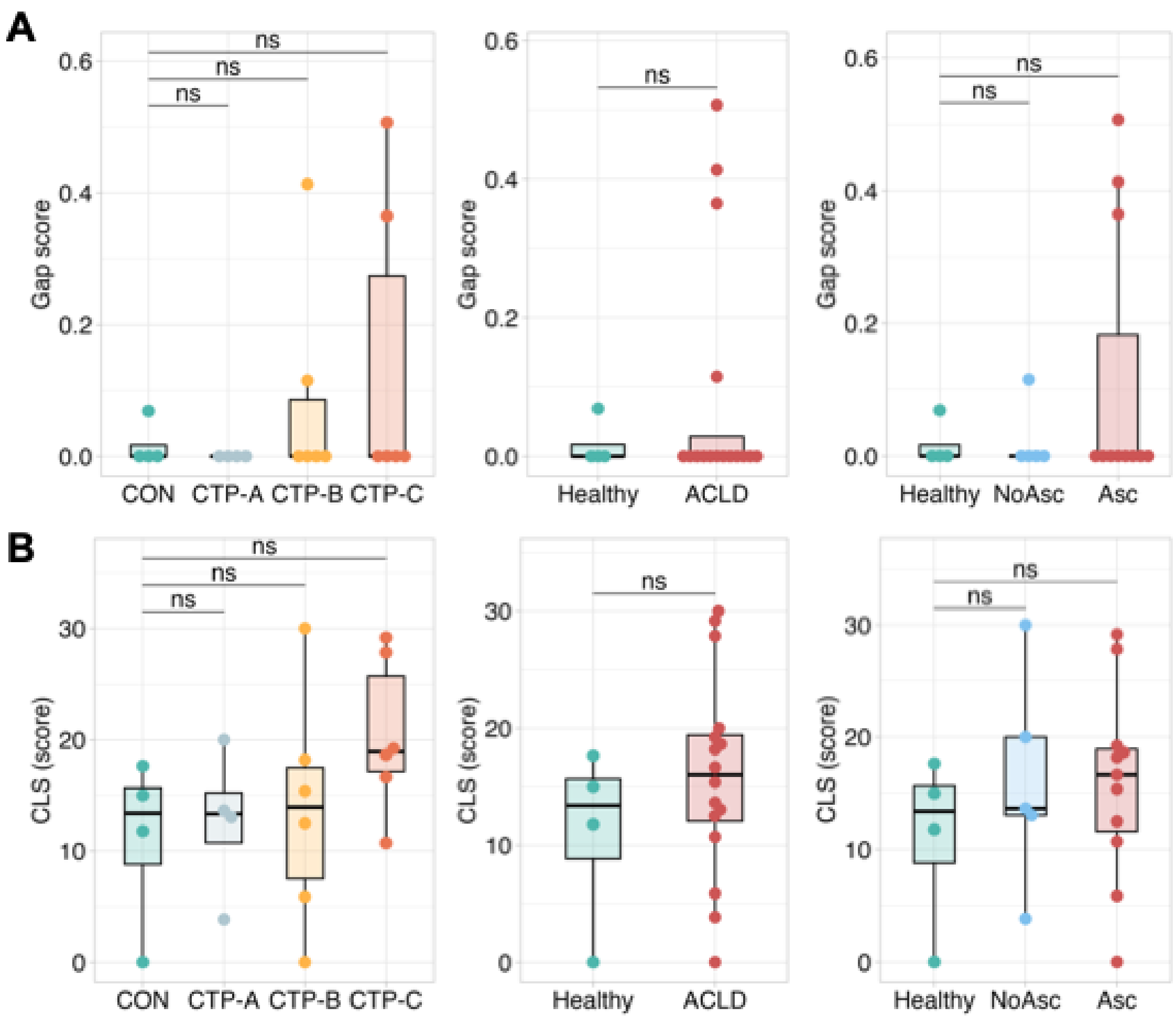

Statistics: Group comparisons were performed using pairwise Wilcoxon rank-sum tests versus the first group (Healthy controls) with Benjamini–Hochberg correction for multiple testing. Figure legend: (ns) not significant; (*) p<0.05; (**) p<0.01; (***) p<0.001. Abbreviations: (ACLD) advanced chronic liver disease; (CLS) confocal leak score; (CON) healthy controls, (CTP) Child-Turcotte-Pugh stage; (Asc) ascites.

Fig. S4. Principal component analysis: variance explained, biplot, and variable contributions (Bern cohort).

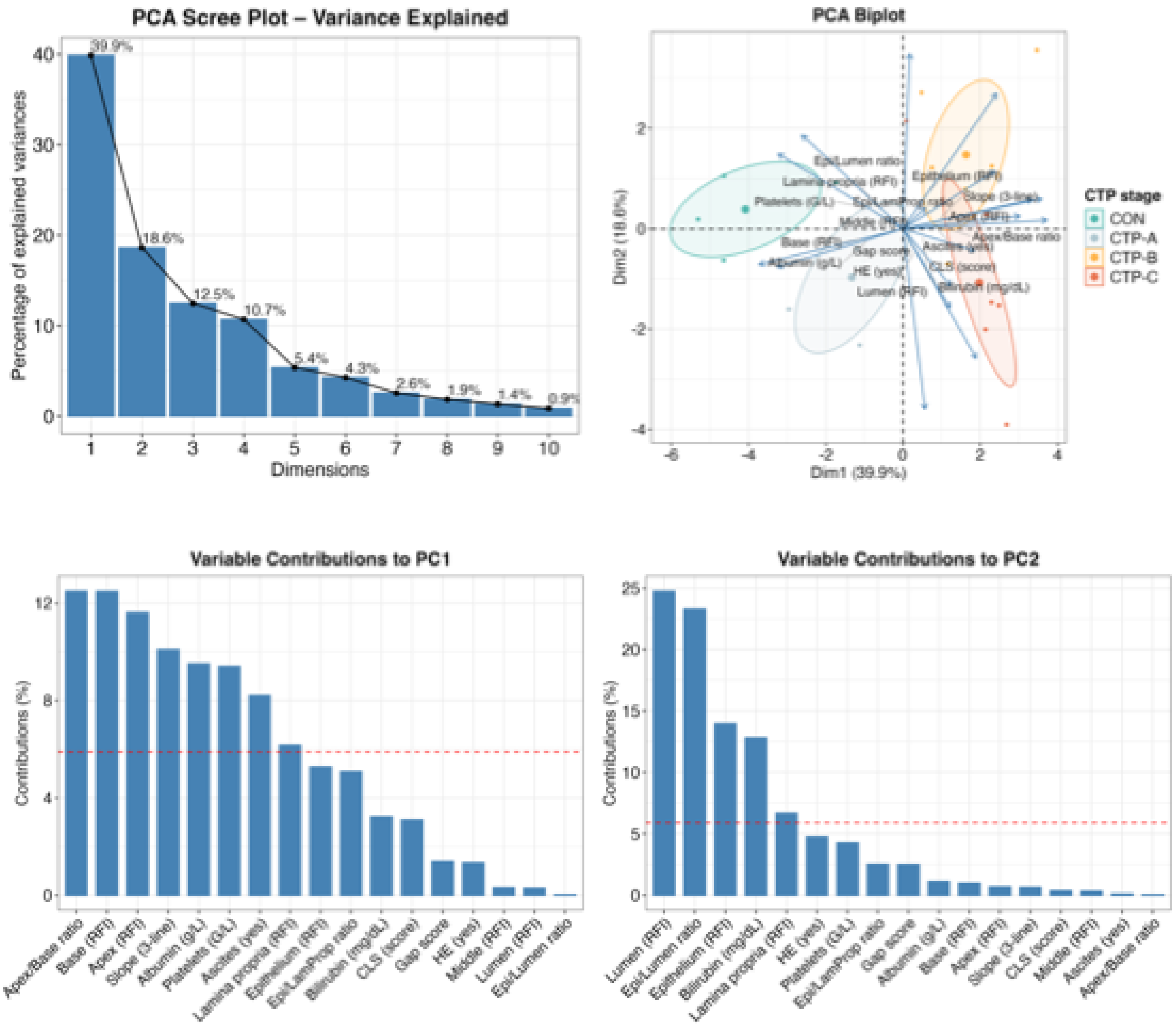

Statistics and Legend: PCA was conducted using a correlation matrix with unit variance scaling; all plots are based on complete-case observations only. Panel 1: Scree plot showing the proportion of variance explained by each principal component. Panel 2: PCA biplot illustrating the joint projection of individuals and variables onto the first two principal components. Individuals are colored by either healthy controls “CON” or patients “CTP stage” with 95% confidence ellipses, while arrows represent variable loadings. Panels 3-4: Bar plots depicting the relative contributions of individual variables to the first (PC1) and second (PC2) principal components. Abbreviations: (RFI) relative fluorescence intensity; (CLS) confocal leak score; (LamProp) lamina propria; (HE) hepatic encephalopathy; (CON) healthy controls, (CTP) Child-Turcotte-Pugh stage.

Fig. S5. Presence of fluorescein leakage in patients stratified by Baveno clinical stages: cACLD, first decompensation, further decompensation (Vienna cohort).

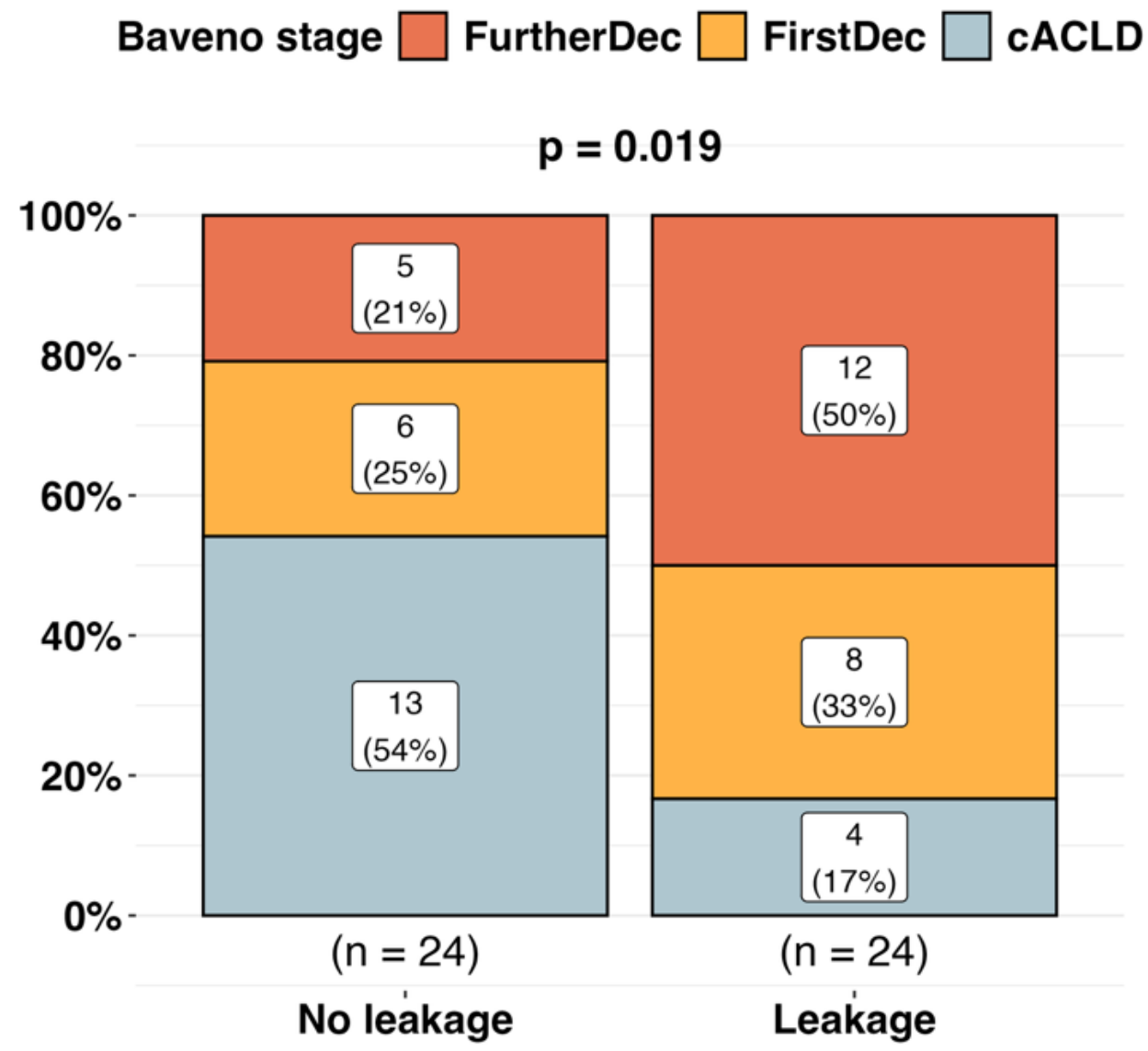

Statistics: Group comparison was performed by Chi squared test. Abbreviations: (cACLD) compensated advanced chronic liver disease, (FirstDec) first hepatic decompensation, (FurtherDec) further hepatic decompensation.

Fig. S6. CLE readouts in patients stratified by (A) CTP stages and (B) Baveno clinical stages: cACLD, first decompensation, further decompensation (Vienna cohort).

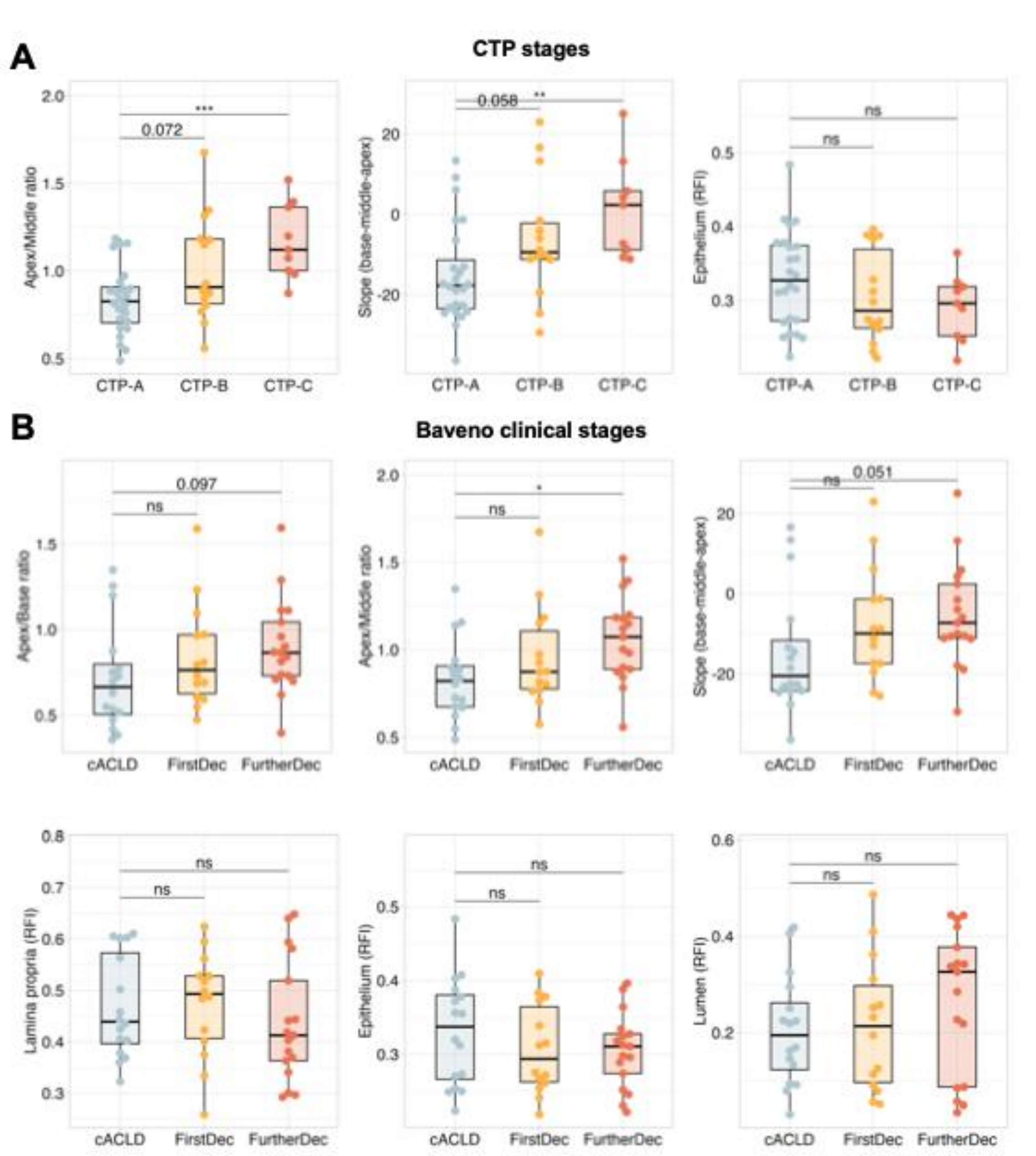

Statistics: Group comparisons were performed using pairwise Wilcoxon rank-sum tests versus the CTP-A/cACLD group with Benjamini–Hochberg correction for multiple testing. Figure legend: (ns) not significant; (*) p<0.05; (**) p<0.01; (***) p<0.001. Abbreviations: (CTP) Child-Turcotte-Pugh, (cACLD) compensated advanced chronic liver disease, (FirstDec) first hepatic decompensation, (FurtherDec) further hepatic decompensation, (RFI) relative fluorescence intensity, (CLE) confocal laser endomicroscopy.

Fig. S7. CLE readouts in patients stratified by the presence of ascites (Vienna cohort).

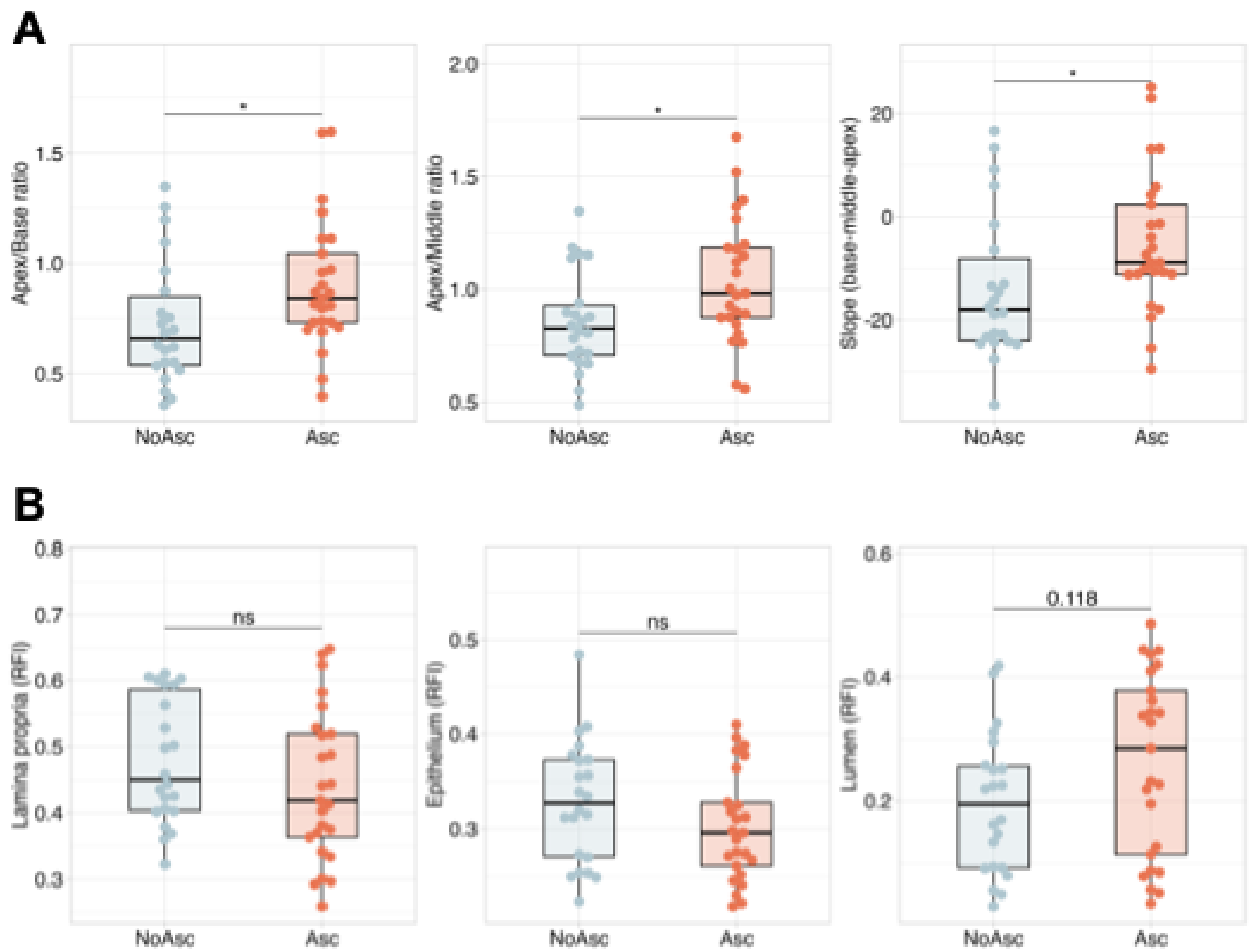

Statistics: Group comparisons were performed using two-sided Wilcoxon rank-sum tests. Figure legend: (ns) not significant; (*) p<0.05; (**) p<0.01; (***) p<0.001. Abbreviations: (Asc) Ascites, (RFI) relative fluorescence intensity, (CLE) confocal laser endomicroscopy.

Fig. S8. Gap score and CLS compared by (A) CTP stages and (B) presence of ascites (Vienna cohort).

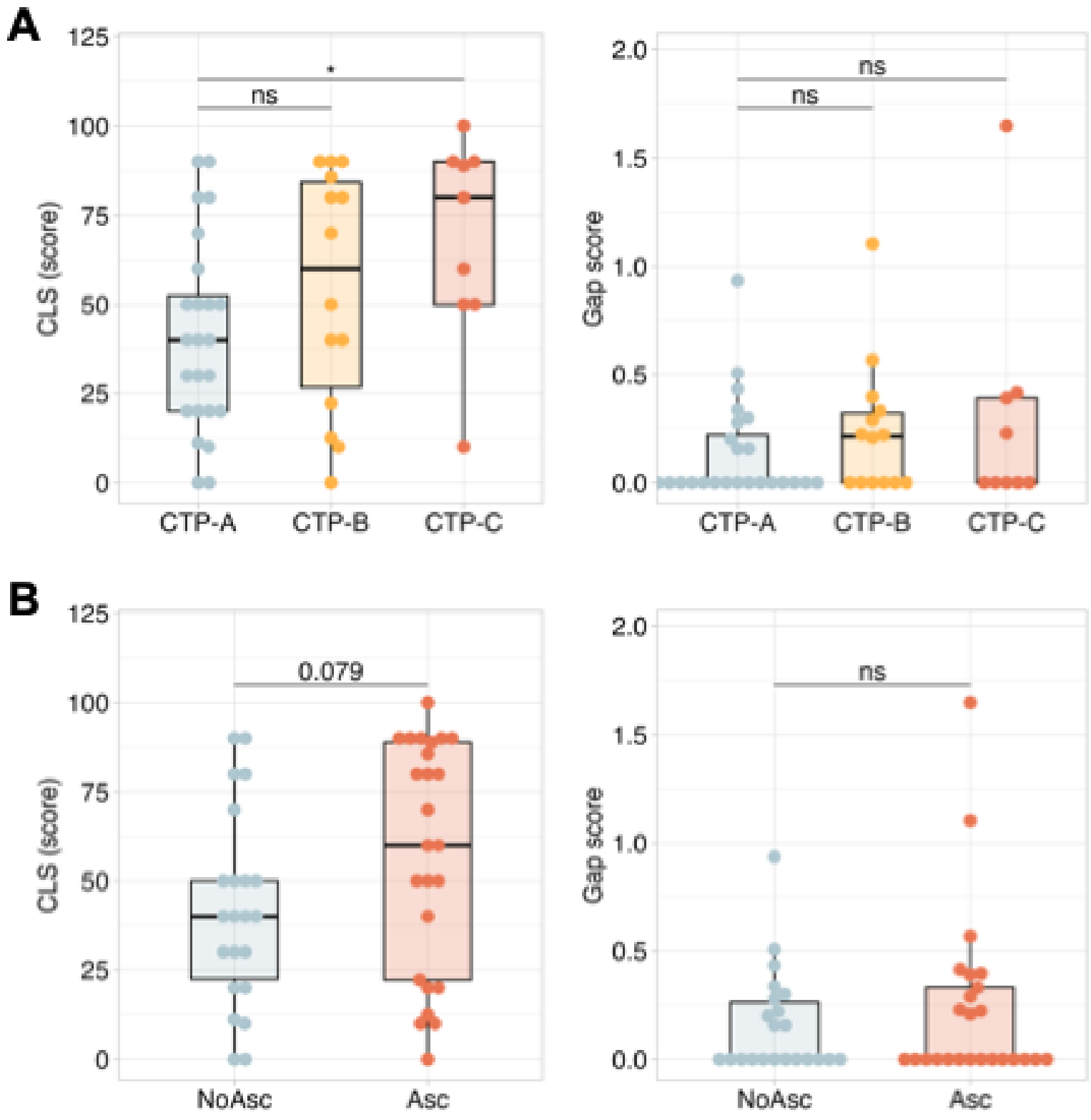

Statistics: Group comparisons were performed using pairwise Wilcoxon rank-sum tests with Benjamini–Hochberg correction for multiple testing. Figure legend: (ns) not significant; (*) p<0.05; (**) p<0.01; (***) p<0.001. Abbreviations: (Asc) ascites, (CTP) Child-Turcotte-Pugh stage, (CLS) Confocal Leak Score.

Fig. S9. Correlation between CLE readouts and markers of liver disease severity and inflammation (Vienna cohort).

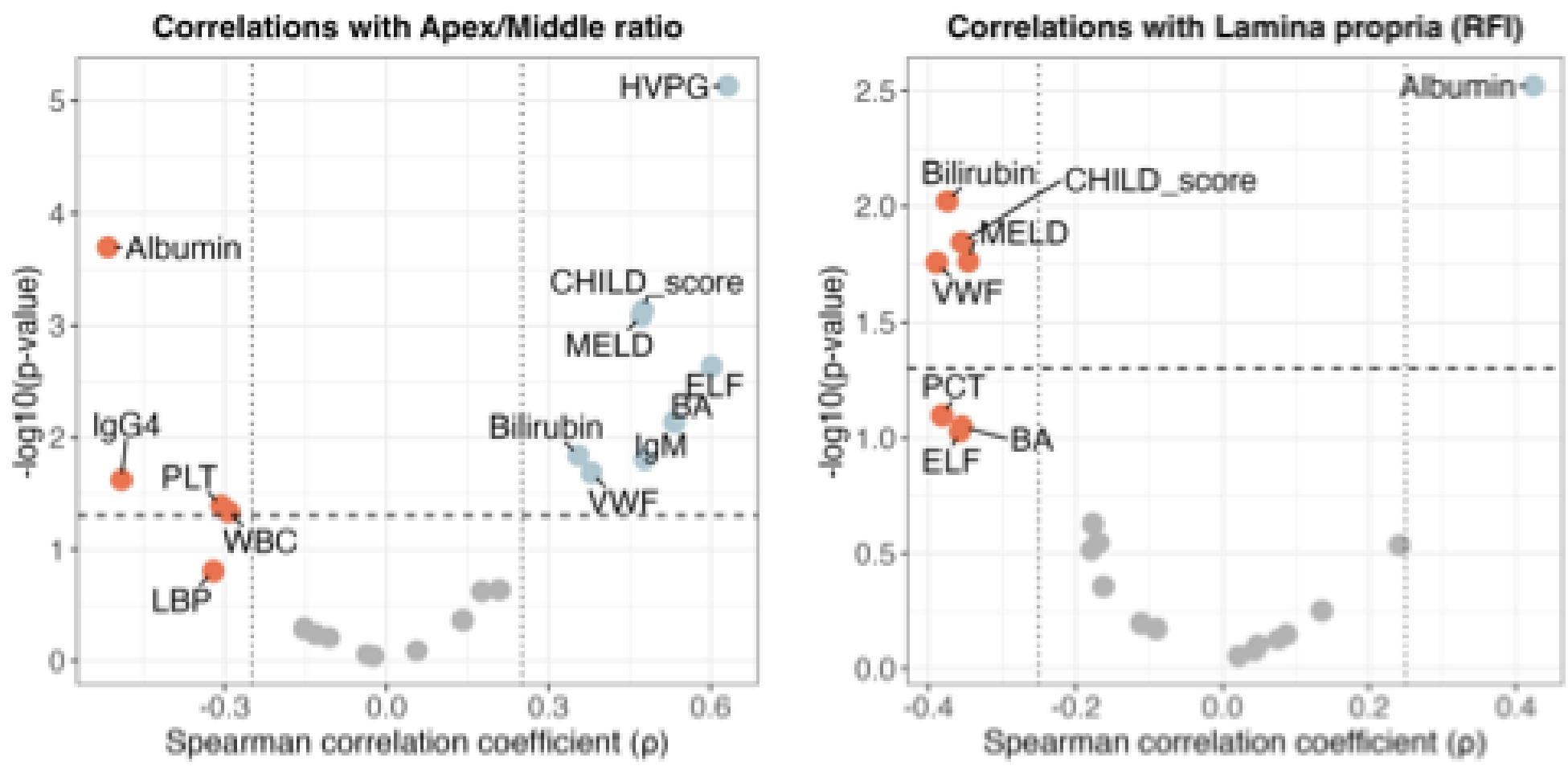

Statistics: Associations were assessed using two-sided Spearman rank correlation tests; p-values are shown without adjustment for multiple testing. Legend: Volcano plots depict Spearman rank correlations between imaging-derived metrics (dependent variables) and clinical and laboratory parameters. The x-axis shows the Spearman correlation coefficient (ρ), and the y-axis shows $-\log_{10}$(p-value). Horizontal dashed lines indicate nominal statistical significance ($p = 0.05$), and vertical dotted lines indicate effect size thresholds ($|\rho| = 0.25$). Points are color-coded by correlation strength and direction (positive correlations in blue, negative correlations in red, weak correlations in grey). Variables with $p < 0.05$ or $|\rho| > 0.25$ are labeled. Abbreviations: (CHILD) Child–Pugh score, (MELD) Model for End-Stage Liver Disease, (HVPG) Hepatic Venous Pressure Gradient, (PLT) platelet count, (VWF) von Willebrand factor, (CRP) C-Reactive Protein, (WBC) White Blood Cells, (PCT) Procalcitonin, (ELF) Enhanced Liver Fibrosis score, (BA) Bile acids, (Ig) Immunoglobulin.

Fig. S10. Principal component analysis: variance explained, biplot, and variable contributions (Vienna cohort).

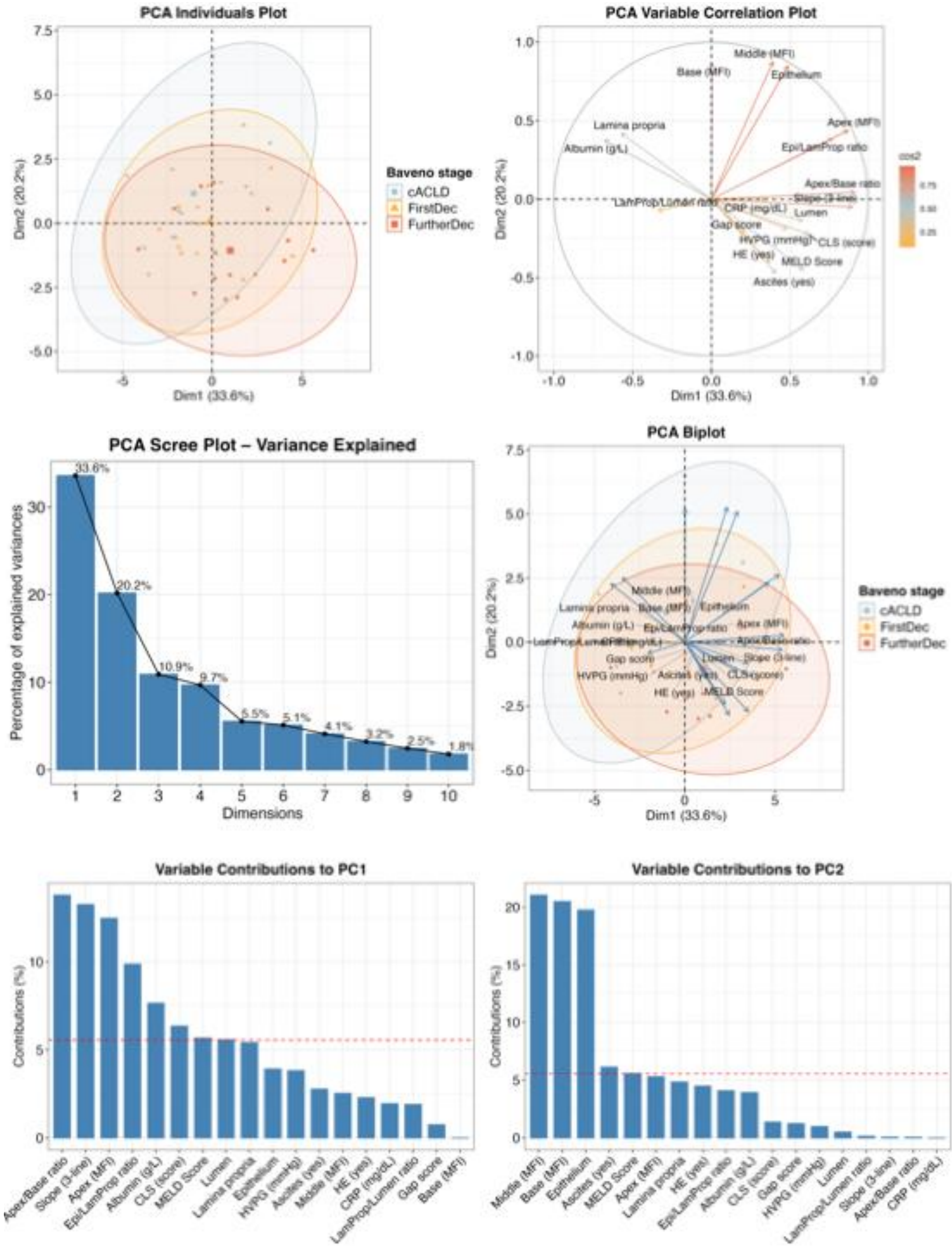

Statistics: PCA was conducted using a correlation matrix with unit variance scaling; all plots are based on complete-case observations. Panel 1: PCA plot illustrating the projection of individual patients onto the first two principal components, colored according to Baveno stage with 95% confidence ellipses. Panel 2: PCA variable correlation plot showing the relationships and loadings of variables across the first two principal components. Panel 3: Scree plot showing the proportion of variance explained by each principal component. Panel 4: PCA biplot illustrating the

joint projection of individuals and variables onto the first two principal components. Individuals are colored by Baveno stage with 95% confidence ellipses, while arrows represent variable loadings. Panels 5-6: Bar plots depicting the relative contributions of individual variables to the first (PC1) and second (PC2) principal components. Abbreviations: (MFI) mean fluorescence intensity, (MELD) Model for End-Stage Liver Disease, (HE) hepatic encephalopathy, (Epi/LamProp) epithelium-to-lamina propria ratio, (CLS) confocal leak score, (HVPG) hepatic venous pressure gradient, (CRP) C-reactive protein.

Fig. S11. Incidence of liver-related events during the follow-up period: Stratification by presence of intraluminal fluorescein leakage (Vienna cohort).

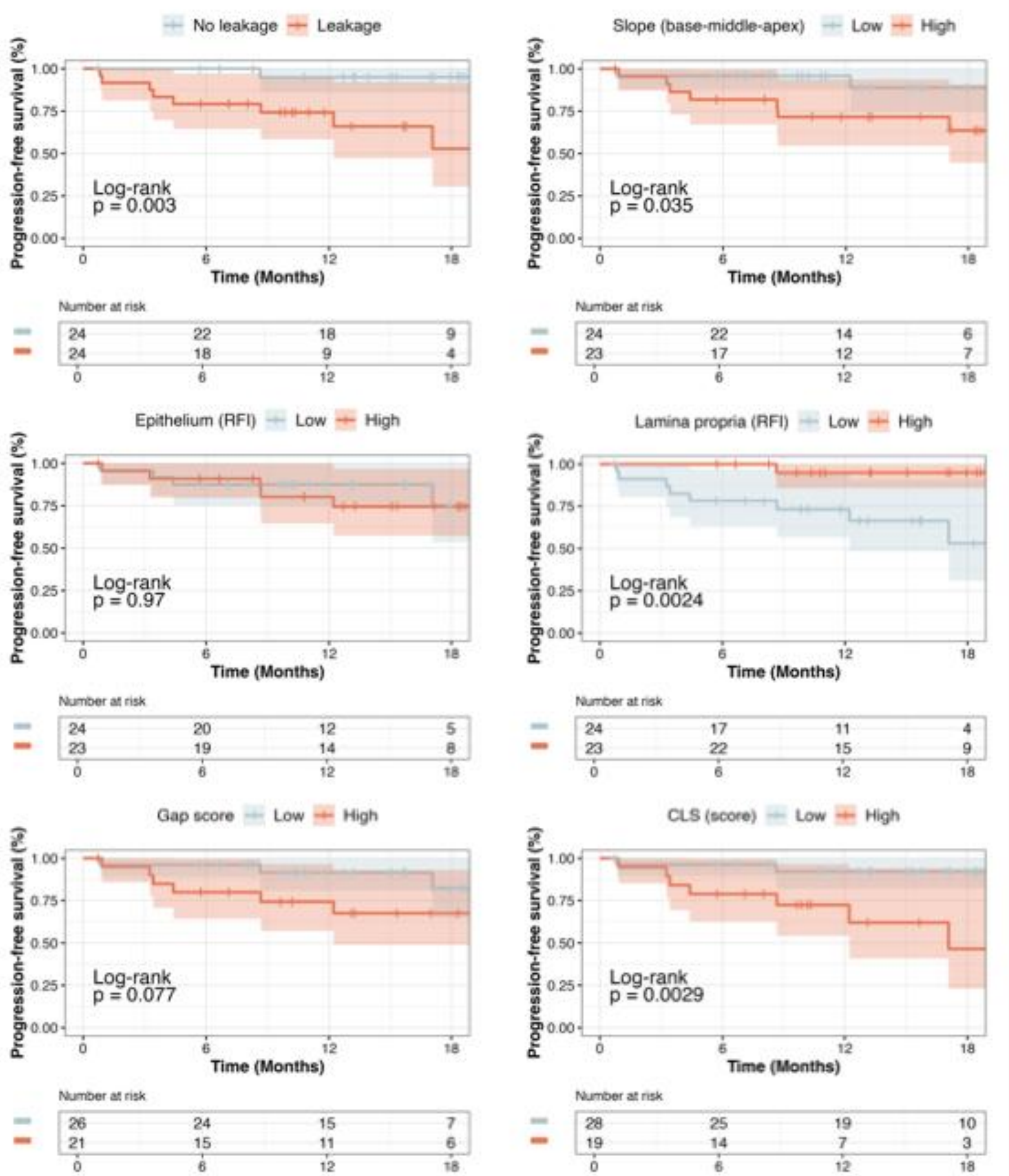

Statistical Analysis: Kaplan-Meier curves indicating disease progression-free follow-up (composite endpoint: first/further decompensation, ACLF, and liver related death) were compared by log-rank test. Panel 1: Groups are defined by presence or absence of intraluminal fluorescein leakage (binary classification). Panel 2-6: Groups are stratified by median CLE parameters (n=1 missing value due to technical issues with white balance of images). Abbreviations: (ACLF) Acute-on-Chronic Liver Failure, (CLE) Confocal Laser Endomicroscopy, (CLS) Confocal Leak Score, (RFI) Relative Fluorescence Intensity.

Fig. S12. Representative images for (A-C) Confocal leak score (CLS) and (D) Gap score.

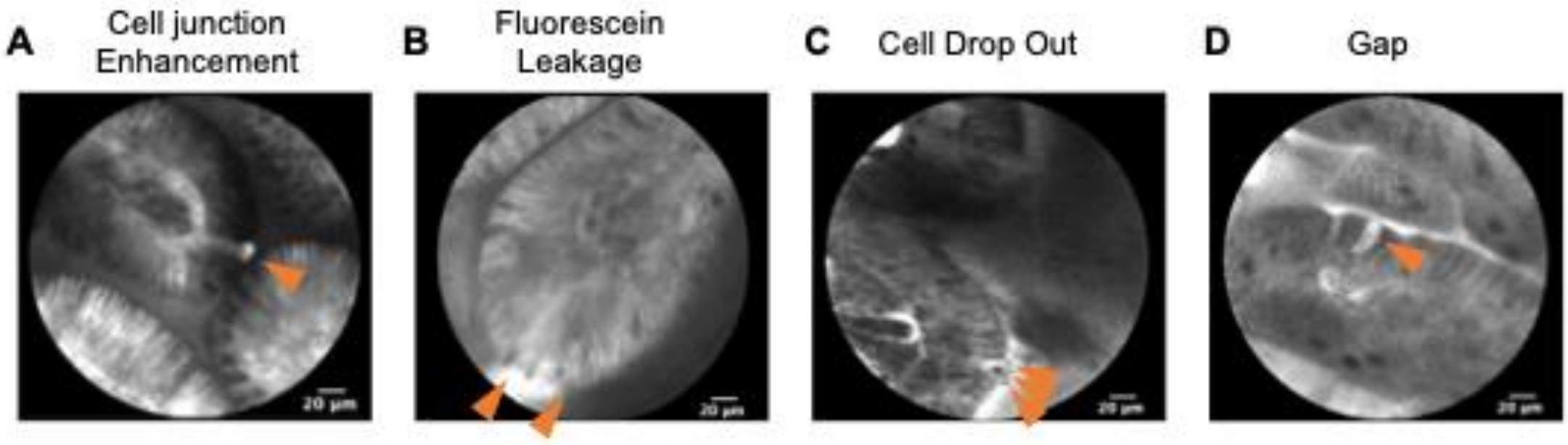

Supplementary tables

Table S1. Patient characteristics in patients stratified by CTP stage (Bern Cohort).

	Healthy<br>*N=4*	CTP-A<br>*N=4*	CTP-B<br>*N=6*	CTP-C<br>*N=6*	p-value
Age (Years)	44.3 (12.7)	61.2 (8.96)	55.5 (12.3)	53.2 (11.5)	0.250
Sex (n, %):					0.211
Female	0 (0.00%)	2 (50.0%)	1 (16.7%)	0 (0.00%)	
Male	4 (100%)	2 (50.0%)	5 (83.3%)	6 (100%)	
Etiology (n, %):					0.013
ALD	---	1 (25.0%)	3 (50.0%)	3 (50.0%)	
Viral	---	1 (25.0%)	0 (0.00%)	0 (0.00%)	
ALD&Viral	---	1 (25.0%)	1 (16.7%)	2 (33.3%)	
MASLD	---	1 (25.0%)	2 (33.3%)	0 (0.00%)	
Other	---	0 (0.00%)	0 (0.00%)	1 (16.7%)	
Ascites (n, %):					<0.001
Absent	---	4 (100%)	1 (16.7%)	0 (0.00%)	
Present	---	0 (0.00%)	5 (83.3%)	6 (100%)	
HE (n, %):					0.194
Absent	---	3 (75.0%)	3 (50.0%)	2 (33.3%)	
Present	---	1 (25.0%)	3 (50.0%)	4 (66.7%)	
CTP score	---	5.25 (0.50)	8.33 (0.82)	11.5 (1.22)	<0.001
MELD Score	---	9.00 [8.75;9.25]	11.0 [10.0;12.8]	24.0 [21.8;27.0]	0.018
Platelets (G/L)	255 (19.9)	142 (76.2)	98.0 (53.0)	68.2 (49.9)	<0.001
Bilirubin (µmol/L)	6.75 [4.80;8.60]	15.5 [11.8;20.2]	13.5 [12.0;21.0]	60.0 [33.5;96.2]	0.005
Albumin (g/L)	35.5 (4.80)	37.8 (3.30)	28.3 (3.83)	26.8 (2.71)	0.001
CRP (mg/L)	0.15 [0.08;0.28]	8.50 [7.62;9.38]	13.5 [10.5;23.2]	4.00 [3.25;10.8]	0.007

Statistical Analysis: Kruskal Wallis test or ANOVA were used to compare continuous variables. Group comparisons of categorical variables were performed using Chi squared or Fisher's Exact test. Abbreviations: (CTP) Child-Turcotte-Pugh; (ALD) Alcohol-Related Liver Disease, (CHOL) Cholestatic Liver Disease, (MASLD) Metabolic Dysfunction-Associated Liver Disease, (MELD) Model for End-Stage Liver Disease, (HE) Hepatic Encephalopathy, (CRP) C-Reactive Protein

Table S2. Patient characteristics across CTP stages (Vienna Cohort).

	A<br>*N=25*	B<br>*N=14*	C<br>*N=9*	p-value
MELD Score	10.0 [8.00;11.0]	13.0 [12.0;15.0]	25.0 [20.0;26.0]	<0.001
HVPG (mmHg)	16.4 (4.77)	18.2 (5.69)	19.0 (3.89)	0.358
Etiology (n, %):				0.290
ALD	13 (52.0%)	6 (42.9%)	7 (77.8%)	
Cholestatic	3 (12.0%)	3 (21.4%)	0 (0.00%)	
MASLD	2 (8.00%)	1 (7.14%)	0 (0.00%)	
ALD&Viral	3 (12.0%)	0 (0.00%)	2 (22.2%)	
Other	2 (8.00%)	4 (28.6%)	0 (0.00%)	
Viral	2 (8.00%)	0 (0.00%)	0 (0.00%)	
Sex (n, %):				0.917
F	7 (28.0%)	5 (35.7%)	3 (33.3%)	
M	18 (72.0%)	9 (64.3%)	6 (66.7%)	
Age (Years)	58.6 (11.6)	50.7 (17.7)	53.8 (10.9)	0.215
Varices (n, %):				0.645
No	7 (28.0%)	3 (21.4%)	1 (11.1%)	
Small	9 (36.0%)	3 (21.4%)	4 (44.4%)	
Large	9 (36.0%)	8 (57.1%)	4 (44.4%)	
Ascites (n, %):				<0.001
No	21 (84.0%)	2 (14.3%)	0 (0.00%)	
Mild	4 (16.0%)	10 (71.4%)	6 (66.7%)	
Severe	0 (0.00%)	2 (14.3%)	3 (33.3%)	
Hepatic Encephalopathy (n, %):				<0.001
No	25 (100%)	12 (85.7%)	3 (33.3%)	
Mild	0 (0.00%)	2 (14.3%)	5 (55.6%)	
Severe	0 (0.00%)	0 (0.00%)	1 (11.1%)	
Albumin (g/L)	40.0 (3.73)	34.0 (3.23)	28.5 (4.22)	<0.001
CRP (mg/dL)	0.27 [0.10;0.54]	0.44 [0.32;0.61]	0.71 [0.58;0.76]	0.044
Gap score	0.00 [0.00;0.22]	0.21 [0.00;0.32]	0.00 [0.00;0.39]	0.465
CLS (score)	40.0 [20.0;52.5]	60.0 [26.7;84.3]	80.0 [50.0;90.0]	0.055
Apex/Base ratio	0.69 [0.53;0.80]	0.83 [0.71;0.94]	1.04 [0.79;1.11]	0.008
Middle/Base ratio	0.79 [0.77;0.88]	0.84 [0.77;0.94]	0.91 [0.77;0.94]	0.597
Apex/Middle ratio	0.83 (0.19)	1.01 (0.30)	1.17 (0.22)	0.002
Epi/LamProp ratio	0.67 [0.57;0.79]	0.68 [0.58;0.77]	0.70 [0.63;0.90]	0.703
Epi/Lumen ratio	2.11 [1.23;3.53]	1.43 [0.66;3.53]	0.73 [0.61;1.05]	0.057
LamProp/Lumen ratio	2.68 [1.58;6.52]	1.71 [1.22;5.82]	1.01 [0.86;1.26]	0.021
Slope (base-middle-apex)	-14.97 (12.3)	-6.03 (15.0)	1.39 (12.3)	0.007
Epithelium (RFI)	0.33 (0.07)	0.30 (0.06)	0.29 (0.05)	0.231
Lamina propria (RFI)	0.49 (0.09)	0.45 (0.11)	0.39 (0.11)	0.058
Lumen (RFI)	0.17 [0.09;0.25]	0.23 [0.09;0.34]	0.36 [0.33;0.42]	0.027
Fluorescein leakage (n, %):				0.011
Absent	17 (68.0%)	6 (42.9%)	1 (11.1%)	
Present	8 (32.0%)	8 (57.1%)	8 (88.9%)	

Statistical Analysis: Kruskal Wallis test or ANOVA were used to compare continuous variables. Group comparisons of categorical variables were performed using Chi squared or Fisher's Exact test. Abbreviations: (CTP) Child-Turcotte-Pugh, (cACLD) compensated advanced chronic liver disease, (MELD) Model for End-Stage Liver Disease, (HVPG) Hepatic Venous Pressure Gradient, (ALD) Alcohol-Related Liver Disease, (MASLD) Metabolic Dysfunction-Associated Liver Disease, (HE) Hepatic Encephalopathy, (CRP) C-Reactive Protein, (CLS) Confocal Leak Score, (RFI) Relative Fluorescence Intensity.

Table S3. Availability of blood biomarkers (Vienna Cohort).

Parameter	N available (total: 48)
WBC (G/L)	48
Bilirubin (mg/dL)	48
Albumin (g/L)	48
CRP (mg/dL)	48
VWF-Ag (%)	38
PCT (ng/mL)	23
ELF score	24
BA (µmol/L)	25
IgG (g/L)	26
IgA (g/L)	26
IgM (g/L)	26
IgG1 (g/L)	22
IgG2 (g/L)	22
IgG3 (g/L)	22
IgG4 (g/L)	22

Abbreviations: (WBC) White Blood Cells, (CRP) C-Reactive Protein, (VWF-Ag) von Willebrand factor antigen, (PCT) Procalcitonin, (ELF) Enhanced Liver Fibrosis score, (BA) Bile acids, (Ig) Immunoglobulin.

Supplementary references

1. Chang, J., et al., *Impaired Intestinal Permeability Contributes to Ongoing Bowel Symptoms in Patients With Inflammatory Bowel Disease and Mucosal Healing.* Gastroenterology, 2017. **153**(3): p. 723-731.e1.
2. Shavrov, A., et al., *A Pilot Study of Confocal Laser Endomicroscopy to Predict Barrier Dysfunction and Relapse in Pediatric Inflammatory Bowel Disease.* J Pediatr Gastroenterol Nutr, 2016. **62**(6): p. 873-8.
3. de Franchis, R., et al., *Baveno VII - Renewing consensus in portal hypertension.* J Hepatol, 2022. **76**(4): p. 959-974.
